# MesKit: a tool kit for dissecting cancer evolution of multi-region tumor biopsies through somatic alterations

**DOI:** 10.1093/gigascience/giab036

**Published:** 2021-05-21

**Authors:** Mengni Liu, Jianyu Chen, Xin Wang, Chengwei Wang, Xiaolong Zhang, Yubin Xie, Zhixiang Zuo, Jian Ren, Qi Zhao

**Affiliations:** School of Life Sciences, Sun Yat-sen University, Guangzhou, Guangdong 510275, China; State Key Laboratory of Oncology in South China, Collaborative Innovation Center for Cancer Medicine, Sun Yat-sen University Cancer Center, 651 E Dongfeng Road, Guangzhou, Guangdong 510060, China; School of Life Sciences, Sun Yat-sen University, Guangzhou, Guangdong 510275, China; School of Life Sciences, Sun Yat-sen University, Guangzhou, Guangdong 510275, China; School of Life Sciences, Sun Yat-sen University, Guangzhou, Guangdong 510275, China; State Key Laboratory of Oncology in South China, Collaborative Innovation Center for Cancer Medicine, Sun Yat-sen University Cancer Center, 651 E Dongfeng Road, Guangzhou, Guangdong 510060, China; School of Life Sciences, Sun Yat-sen University, Guangzhou, Guangdong 510275, China; State Key Laboratory of Oncology in South China, Collaborative Innovation Center for Cancer Medicine, Sun Yat-sen University Cancer Center, 651 E Dongfeng Road, Guangzhou, Guangdong 510060, China; School of Life Sciences, Sun Yat-sen University, Guangzhou, Guangdong 510275, China; State Key Laboratory of Oncology in South China, Collaborative Innovation Center for Cancer Medicine, Sun Yat-sen University Cancer Center, 651 E Dongfeng Road, Guangzhou, Guangdong 510060, China; State Key Laboratory of Oncology in South China, Collaborative Innovation Center for Cancer Medicine, Sun Yat-sen University Cancer Center, 651 E Dongfeng Road, Guangzhou, Guangdong 510060, China

**Keywords:** multi-region sequencing, somatic alterations, intra-tumor heterogeneity, metastatic routes, phylogenetic tree

## Abstract

**Background:**

Multi-region sequencing (MRS) has been widely used to analyze intra-tumor heterogeneity (ITH) and cancer evolution. However, comprehensive analysis of mutational data from MRS is still challenging, necessitating complicated integration of a plethora of computational and statistical approaches.

**Findings:**

Here, we present MesKit, an R/Bioconductor package that can assist in characterizing genetic ITH and tracing the evolutionary history of tumors based on somatic alterations detected by MRS. MesKit provides a wide range of analysis and visualization modules, including ITH evaluation, metastatic route inference, and mutational signature identification. In addition, MesKit implements an auto-layout algorithm to generate phylogenetic trees based on somatic mutations. The application of MesKit for 2 reported MRS datasets of hepatocellular carcinoma and colorectal cancer identified known heterogeneous features and evolutionary patterns, together with potential driver events during cancer evolution.

**Conclusions:**

In summary, MesKit is useful for interpreting ITH and tracing evolutionary trajectory based on MRS data. MesKit is implemented in R and available at https://bioconductor.org/packages/MesKit under the GPL v3 license.

## Introduction

Cancer evolves through a process of somatic alterations [1], of which spatial and/or temporal changes can be detected by multi-region sequencing (MRS). Currently, MRS has become an effective and affordable way to trace the evolutionary history of carcinogenesis and metastasis. Cancer evolution research is focused on the identification and estimation of intra-tumor heterogeneity (ITH), phylogenetic reconstruction, mutational signature analysis, and so forth. Numerous MRS studies have identified extensive ITH among many solid tumors originating in the liver, prostate, esophagus, breast, and lung [2–7]. In addition, increased ITH has been implicated in dismal cancer prognosis [8–10]. While recent studies have largely generated descriptive summaries of ITH, a quantitative understanding of the heterogeneity within and between tumors from the same patient is more informative for personal therapeutics.

Recently, plenty of MRS studies have used phylogenetic trees to show the temporal sequence and heterogeneous divergence between samples [2, 11, 12]. There are also increasing efforts to reconstruct subclonal phylogenies via a “clone tree,” which summarizes lineage relationships between cellular subpopulations [13–15]. Phylogenetic reconstruction over the cancer cell fraction (CCF) estimates has identified both monoclonal and multiclonal seeding patterns in several cancers [3, 16–18]. The distinction between these 2 patterns may have important clinical implications [19]; it is thus necessary to infer metastatic routes and to explore potential metastasis drivers.

Moreover, MRS provides insights into the dynamics of mutational processes during tumor progression. A previous study indicated that DNA damage repair dysfunction might be crucial for mutation accumulation during osteosarcoma evolution [20]. Recently, Yan et al. [21] performed MRS of tumors from 39 patients with esophageal squamous cell carcinoma and identified several potential actionable targets, such as *EGFR* and *FGFR1*. They also showed that APOBEC mutations and aging predominated in the early stage of tumorigenesis of esophageal squamous cell carcinoma. These findings suggest that the MRS strategy has the potential to reveal mutational mechanisms and thereby could improve both diagnosis and treatment.

The downstream analysis of MRS data focuses on somatic alterations, including somatic single-nucleotide variants (sSNVs), small insertions and deletions (INDELs), and copy number alterations (CNAs). At present, many tools are available to analyze somatic alterations, which has greatly promoted the development of cancer genomics. For example, Maftools [22] provides multiple functions for pathway annotation and *de novo* signature and enrichment analysis. MutationalPatterns [23] and deconstructSigs [24] are powerful tools for exploring mutational patterns and identifying mutational signatures of a single tumor sample. Besides, Palimpsest [25] enables the identification of different mutational signatures between clonal and subclonal mutations. In addition, lots of methods infer tumor heterogeneity by assessing the complex subclonal structure of tumors. Approaches such as SciClone [26], PhyloSub [27], and PyClone [28] are based solely on point mutations (sSNVs or INDELs), while SCHISM [29], DPClust [30], and PhyloWGS [31] adjust for CNAs in their models in different ways. In general, high-depth sequencing improves the accuracy of subclonal reconstruction and resolution [32]. However, performing integrated mutational analysis of MRS using these tools is inconvenient because different preprocessing steps and inconsistent input formats are required. On the other hand, it is laborious and time-consuming to generate publication-quality images such as mutational profiles and phylogenetic trees, which necessitates manual modifications using extra graphic editors.

To address these concerns, we present MesKit, an R/Bioconductor package that provides commonly used analysis and visualization modules for MRS studies. MesKit was designed as an easy-to-use R package that only requires a MAF file and a clinical file as inputs, enabling researchers to evaluate the contribution of point mutations to heterogeneity within/between tumors from the same patient. MesKit can also be used to depict mutational profiles, track evolutionary dynamics, and characterize mutational patterns at different levels. Notably, we implemented an auto-layout algorithm to visualize rooted phylogenetic trees with annotations. In addition, MesKit enables easy integration and analysis of segmentation data and CCF data and a Shiny application is provided to facilitate interactive analysis. Finally, we applied MesKit on 2 high-quality MRS datasets of hepatocellular carcinoma (HCC) [2] and colorectal cancer (CRC) [12] (Supplementary Table S1). We reproduced well-known heterogeneous features and evolutionary patterns, together with potential driver events of HCC and CRC, demonstrating the robustness of MesKit in interpreting ITH and for inferring evolutionary trajectories based on MRS data.

## Materials and Methods

### Data collection and preprocessing

We used 2 cohorts in our analysis. The HCC cohort included tumor tissue (n = 52) and matched blood (germline, n = 11) samples from 11 patients, which were collected before treatment [2]. All samples were sequenced using whole-exome sequencing (mean depth of 158×) and re-analyzed with a uniform pipeline described below. In brief, we performed sSNV calling for each tumor/normal pair with Mutect (version 1.1.7) [33], while INDELs were detected with Strelka v2.7.1 [34]. Additionally, we adopted the “force calling” method [35] to rescue potential real mutations for each sample based on the aggregate set of somatic events in each patient using Samtools mpileup (version 1.2) [36]. Both sSNVs and INDELs were annotated through ANNOVAR (v.20191024) [37]. The following filters were further applied to identify the sSNVs and INDELs: (i) Mutations with <15 total reads or 5 variant reads were discarded. (ii) Mutations listed in dbSNP147 were removed unless they were documented in the Catalogue of Somatic Mutations in Cancer (COSMIC) database. (iii) Mutations listed in the National Heart, Lung, and Blood Institute Exome Sequencing Project were removed. Copy number analysis based on exome sequencing was performed using Sequenza v3.0.0 [38]. Segments smaller than 500 kb were filtered and only autosomes were used in copy number analysis. CCFs of mutations were estimated by PyClone (v0.13.0) [28], which adjusted the variant allele frequencies (VAFs) of somatic mutations on the basis of local copy numbers of the mutated loci and tumor purity (Sequenza v3.0.0) [38]. The second cohort (the CRC cohort) comprised 6 patients processed with MRS for paired primary tumors and metastases (3–5 regions each) [12]. We obtained somatic mutation calls for sSNVs and INDELs, copy number segment data, and CCF estimates of mutations from the original study. Driver genes of HCC and CRC were defined by IntOGen (v.2020.2). The GISTIC2 results of The Cancer Genome Atlas (TCGA) HCC and TCGA CRC projects were obtained from the Broad Genome Data Analysis Center (GDAC, http://gdac.broadinstitute.org) repository (analysis stamp: 2016_01_28).

### Clonal status of somatic mutations

Because multiple samples collected from a single tumor collectively reflect its clonal composition, these regions should be considered as a whole to capture the overall tumor dynamics. Here, we assume that each tumor follows neutral exponential growth in a well-mixed population [39, 40]. When MRS data are available, the merged CCF (CCF_merged_) of each mutation is computed by integrating multiple regions as previously described [12, 41, 42]:

(1) \begin{eqnarray*}
\mathrm{CCF}_{\mathrm{merged}} = \left \{ \begin{array}{@{}*{1}{c}@{}} {\frac{{\mathop \sum \nolimits_{i = 1}^k \mathrm{CCF}_i\,\, \times \,\,{d_i}}}{{\mathop \sum \nolimits_{i = 1}^k \,\,{d_i}}}\,\,\,\,\mathrm{CCF} < 1}\\ {\,\,\,\,\,\,\,\,\,\,\,\,\,\,\,\,\,\,1\,\,\,\,\,\,\,\,\,\,\,\,\,\,\,\,\,\,\,\,\,\,\mathrm{CCF} \ge 1} \end{array}\,\,\right. \end{eqnarray*}

where ${d_i}$ and ${\mathrm{CC}}{{\mathrm{F}}_i}$ refer to the sequencing depth and CCF estimation in region *i*, respectively. The clonal status of sSNVs/INDELs are determined based on CCFs. A CCF value of 1 indicates that the mutation is present in 100% of the cancer cells in a sample, while a CCF value <1 indicates that the mutation is present in a subset of the cancer cells in a sample and thus is subclonal. In each sample, a mutation is classified as clonal when the upper bound of the 95% confidence interval (CI) of the CCF is ≥1 and subclonal otherwise [43]. For MRS data, a mutation is considered subclonal when all of the following criteria are satisfied: (i) ≥1 region with upper bound of 95% CI of the CCF <1, (2) ≥1 region with CCF < 0.5, and (iii) CCF_merged_ of mutation *m* < 0.5 (the cut-off was chosen for its good performance in defining subclonality in simulated virtual tumors [44, 45]).

### Estimation of ITH

MesKit includes several measures of ITH defined by recent genomic studies. For a single region/tumor, it is common to infer subpopulations of tumor cells by clustering VAFs or CCFs [26, 30]. To implement this process, we used Gaussian finite mixture models for 1D clustering of VAFs or CCFs using mclust R package [46]. Because copy number gains and losses can alter the fraction of reads bearing a mutation, we only focused on heterozygous mutations within copy number–neutral and loss-of-heterozygosity–free regions when clustering VAFs. More recently, Mroz et al. [47] developed the mutant-allele tumor heterogeneity (MATH) index, which corresponds to the ratio of the median absolute deviation (MAD) and the median of the VAF values among tumor-specific mutated loci. Generally, a more heterogeneous tumor with a higher MATH score tends to have a wider distribution of VAFs among all mutation loci and centers at a lower fraction.

(2) \begin{eqnarray*}
\mathrm{MATH}\,\, = \,\,1.4826 \times \frac{{\mathrm{MAD}\left( {\mathrm{VAF}} \right)}}{{\mathrm{Median}\left( {\mathrm{VAF}} \right)}}. \end{eqnarray*}

Another approach to estimate ITH is calculating the area under the curve (AUC) of the cumulative density function based on the CCFs per tumor, and tumors with higher AUC values are considered to be more heterogeneous [48]. Moreover, to quantify the genetic divergence of ITH between regions or tumors, we introduced 2 classical metrics derived from population genetics, Wright fixation index (*F*_ST_) [49] and Nei genetic distance [50]. Calculations of between-region genetic heterogeneity within tumors only consider subclonal mutations because clonal mutations present in all regions do not contribute to ITH. For pairwise comparisons of heterogeneity between tumors, both clonal and subclonal mutations were taken into consideration. The *F*_ST_ index estimating between-region ITH for *k* regions was computed as described previously [44]:

(3) \begin{eqnarray*}
{F_{{\mathrm{ST}}}} = \,\,\frac{1}{r}\,\, \times \,\,\mathop \sum \nolimits_{j\,\, = \,\,1}^r {F_{\mathrm{ST}}}_j^{\mathrm{Hudson}},\,\,r\,\, = \left( {\begin{array}{@{}*{1}{c}@{}} k\\ 2 \end{array}} \right),\,\, \end{eqnarray*}

 

(4) \begin{eqnarray*}
{F_{\mathrm{ST}}}_j^{\mathrm{Hudson}}\,\, = \,\,\frac{{\mathop \sum \nolimits_{m\,\, = \,\,1}^{{m_t}} \,\,{{\left( {f_a^m\,\, - \,\,f_b^m} \right)}^2}\,\, - \,\,\frac{{f_a^m\,\, \times \,\,\left( {1\,\, - \,\,f_a^m} \right)}}{{d_a^m\,\, - \,\,1}}\,\, - \,\,\frac{{f_b^m\,\, \times \,\,\left( {1\,\, - \,\,f_b^m} \right)}}{{d_b^m\,\, - \,\,1}}}}{{\mathop \sum \nolimits_{m\,\, = \,\,1}^{{m_t}} f_a^m\,\, \times \,\,\left( {1 - \,\,f_b^m} \right)\,\, + \,\,f_b^{m\,\,} \times \,\,\left( {1\,\, - \,\,f_a^m} \right)}}, \end{eqnarray*}

where ${m_t}$ represents the total number of sSNVs in regions *a* and *b*, $f_a^m$ denotes the VAF for sSNV *m*, and $d_a^m$ denotes the sequencing depth for sSNV *m* in region *a*.

The Nei genetic distance for *k* regions within the same tumor was defined as follows [50]:

(5) \begin{eqnarray*}
{D_{\mathrm{Nei}}} = \,\,\frac{1}{r}\,\, \times \,\,\mathop \sum \nolimits_{j\,\, = \,\,1}^r {D_{\mathrm{Ne}{\mathrm{i}_j}}},\,\,r\,\, = \left( {\begin{array}{@{}*{1}{c}@{}} k\\ 2 \end{array}} \right),\,\, \end{eqnarray*}

 

(6) \begin{eqnarray*}
{D_{\mathrm{Ne}{\mathrm{i}_j}}} = \,\, - \log \frac{{\mathop \sum \nolimits_{m\,\, = \,\,1}^{{m^t}} \mathrm{ccf}_a^m\,\, \times \,\,\mathrm{ccf}_b^m + \left( {1 - \,\,\mathrm{ccf}_a^m} \right)(1\,\, - \,\,\mathrm{ccf}_b^m)}}{{\sqrt {\left[\mathop \sum \nolimits_{m\,\, = \,\,1}^{{m^t}} \mathrm{ccf}{{_a^m}^2} + {{\left( {1 - \,\,\mathrm{ccf}_a^m} \right)}^2}\right]\,\, \times \left[\mathop \sum \nolimits_{m\,\, = \,\,1}^{{m^t}} \mathrm{ccf}{{_b^m}^2} + {{\left( {1 - \,\,\mathrm{ccf}_b^m} \right)}^2}\right]} }}, \end{eqnarray*}

where ${m_t}$ represents the total number of sSNVs in regions *a* and *b*. $\mathrm{ccf}_a^m$ and $\mathrm{ccf}_b^m$ represent the CCF values in region *a* and region *b* for mutation *m*, respectively.

### Inference of metastatic routes

For spatially separated lesions from the same patient, the potential metastatic route can be determined by comparing subclonal architecture between paired lesions. Here, MesKit integrated a Jaccard similarity index (JSI)-based method to identify seeding patterns based on the CCFs of sSNVs for paired lesions [42]. The Jaccard coefficient for a lesion pair $( {a,\,\,b} )$ is calculated as follows:

(7) \begin{eqnarray*}
\mathrm{JSI}\,\, = \,\,\frac{{\mathrm{S}{\mathrm{S}_{\mathrm{ab}}}}}{{\mathrm{P}{\mathrm{C}_a} + \mathrm{P}{\mathrm{C}_b} + \mathrm{S}{\mathrm{S}_{\mathrm{ab}}}}}, \end{eqnarray*}

where SS_ab_ and PC_*a*_/PC_*b*_ represent shared subclonal sSNVs of lesion pair $( {a,\,\,b} )$ and private clonal sSNVs of lesion $a/b$, respectively. The mean SS_ab_, and PC_*a*_/PC_*b*_ of all sample pairs from lesion *a* and lesion *b* are used to compute the JSI for lesions with MRS data.

### Construction and visualization of phylogenetic trees

MesKit reconstructs the phylogeny of multiple specimens from individual patients on the basis of the presence or absence of somatic mutations. This process is implemented in getPhyloTree function via utilization R implementations of several standard phylogenetic approaches from the APE [51] and PHANGORN [52] R packages, including distance-based methods (neighbor-joining [NJ] [53] and minimum evolution [54]), as well as character-based methods (maximum parsimony [MP] [55] and maximum likelihood [ML] [56]). Notably, we implemented an auto-layout algorithm via the plotPhyloTree function to generate customizable images of phylogenetic trees with annotations (Supplementary File S1). Furthermore, by using the treedist function from the PHANGORN [52] R package, MesKit enables the comparison of phylogenetic trees constructed by different methods via the compareTree function.

### Mutational signature analysis

To illustrate the dynamic mutational spectrum during tumor progression, we implemented mutational signature analysis based on phylogenetic trees. The process starts with the construction of a mutation matrix accounting for 96 trinucleotide changes, where the sequence context of the base substitutions can be retrieved from the corresponding reference genome using the BSgenome R package. Six types of base substitution types are distinguished by convention: C>A, C>G, C>T, T>A, T>C, and T>G. As methylated cytosine at CpG sites with the attendant risk of spontaneous deamination are mutagenic hot spots in the human genome [57], C>T mutations can be divided into C>T at CpG sites and other sites [23]. Genomic mutations are temporally dissected into truncal (shared among all samples from the same patient) and branch mutations of phylogenetic trees. For each mutational type, the Fisher exact test is implemented to assess the difference between the truncal and branch mutations. Once the signature matrix is provided, the fitSignatures function estimates the optimal contributions of known signatures to reconstruct a mutational profile, which minimizes the residual sum of squares (RSS) between the original and reconstructed mutational profiles. This process was implemented by integrating a non-negative least-squares algorithm using the pracma R package [77], as previously described in MutationalPatterns [23]. For convenience, we included known signature matrices (published by Alexandrov et al. [73] and Cosmic version 2, 3) along with the proposed etiology in MesKit. The similarity between mutational profiles A and B is calculated by cosine similarity as follows:

(8) \begin{eqnarray*}
\mathrm{sim}\left( {A,B} \right)\,\, = \,\,\frac{{\mathop \sum \nolimits_{i\,\, = \,\,1}^n {A_i}{B_i}}}{{\sqrt {\mathop \sum \nolimits_{i\,\, = \,\,1}^n A_i^2} \sqrt {\mathop \sum \nolimits_{i\,\, = \,\,1}^n B_i^2} }}, \end{eqnarray*}

where mutational profiles *A* and *B* are non-zero vectors with *n* mutational types. Cosine similarity value can be used to test how well each mutational profile can be explained by the provided mutational signatures. Two mutational profiles are identical when the cosine similarity is 1 and are independent when the cosine similarity is 0.

## Results

### Overview of MesKit functions and implementation

**Figure 1:**
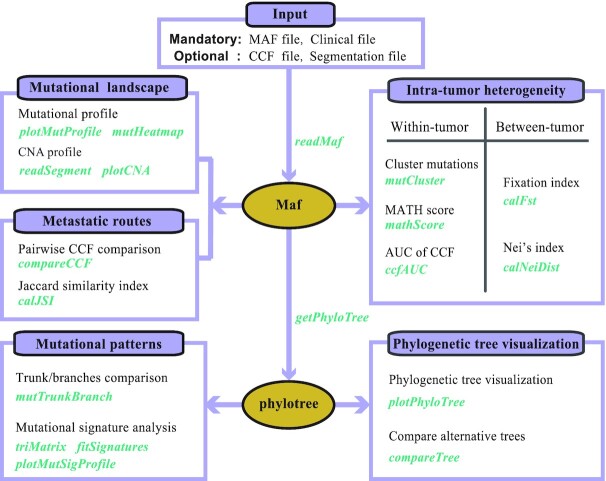
Overview of the MesKit package **A**. Overview of MesKit. MesKit consists of 5 major modules: characterizing mutational landscape, estimating ITH, inferring metastatic routes, exploring mutational patterns, and visualizing phylogenetic trees automatically. Corresponding functions for each module are displayed separately.

MesKit was implemented as an open source R/Bioconductor package. With a MAF file and a clinical data file as standard inputs, MesKit provides a series of analysis and visualization functions to interpret mutational data from MRS experiments (Fig. 1). In addition, we implemented a Shiny application to facilitate the use of the package. Moreover, we built a Docker image that enables the deployment of the Shiny-based MesKit GUI in a C/S mode.

### Mutational landscape of MRS studies

**Figure 2:**
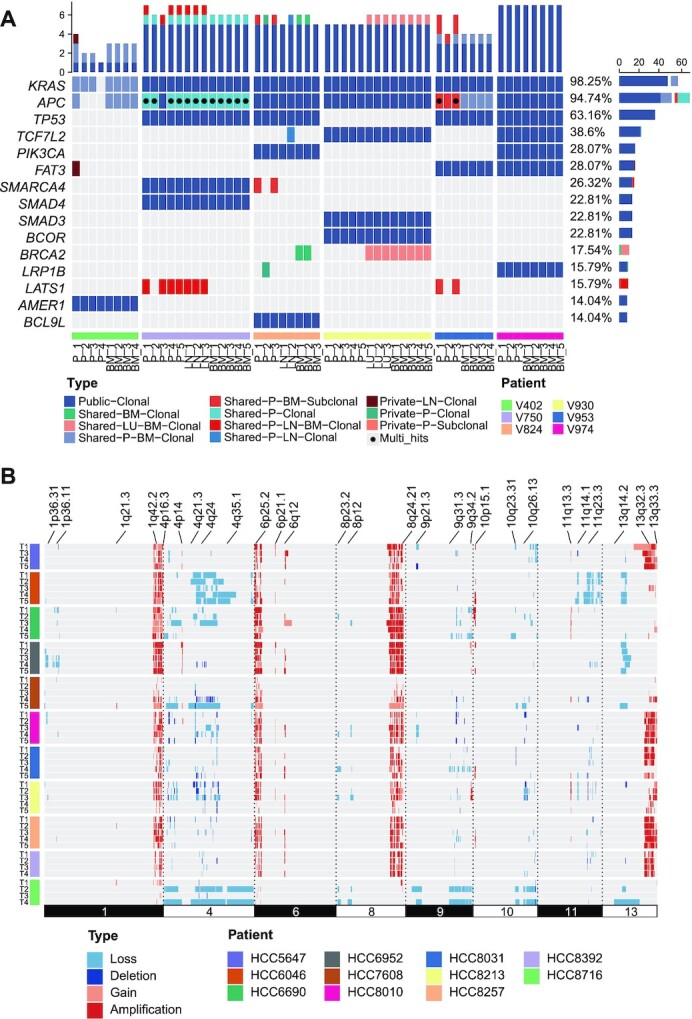
Mutational landscape of the HCC and CRC cohorts **A**. Mutational profile of the CRC cohort. The oncoprint of the top 15 most frequently mutated driver genes of CRC grouped by public, shared, or private mutations, including both clonal and subclonal drivers. Genes were sorted by mutational frequency, and those with multiple mutations were annotated as Multi_Hit. Samples were split by patients as indicated by the annotation bar (bottom). BM: brain metastasis; LN: lymph node metastasis; LU: lung metastasis; P: primary tumor. The stacked bar charts on the top and right show the number of different types of mutations per sample and per driver gene, respectively. **B**. The consistent CNAs of the HCC cohort with significant recurring CNAs were identified from the TCGA hepatocellular carcinoma project by GISTIC2.0 (obtained from the Broad GDAC website). Each track represents 1 tumor sample. Dark red indicates amplifications (CN  ≥  4), light red indicates gains (2  <  CN  <  4), dark blue indicates deletions (CN  =  0), and light blue indicates losses (0  <  CN  <  2).

Generally, somatic mutations identified from MRS in a single tumor are classified as “public mutations” (existing in all regions of the tumor), “shared mutations” (existing in part of all regions), or “private mutations” (existing in a single region) [20, 44, 58]. Such spatial-mutation categories largely correspond to the temporal order of mutation genesis during tumor evolution: most public mutations occur early in tumor-initiating cells and are inherited by their offspring, whereas private mutations accumulate sporadically and markedly increase the ITH among different patients [59]. In MesKit, we implemented the classifyMut function to help categorize somatic mutations based on regional distribution, and/or to identify clonal and subclonal mutations according to their estimated CCFs (Methods). Analysis of the HCC and CRC cohorts showed significant inter-individual heterogeneity but much less intra-individual heterogeneity (Fig. 2 and Supplementary Fig. S1). In line with previous findings [13, 60, 61], the primary tumors and metastases of the CRC cohort exhibited high genomic concordance (Fig. 2A). As expected, public mutations harbor higher CCFs than private mutations (Supplementary Fig. S2), which were more likely to be clonal events. Recurrent mutations in putative driver genes of CRCs (defined by IntOGen v.2020.2), such as *KRAS* and *APC*, were clonal and shared between paired primary tumors and metastases, indicating their early occurrence in colorectal carcinogenesis (Fig. 2A and Supplementary Fig. S2). Interestingly, heterozygous *BRCA2* mutations were private to distant metastases, including the lung metastases (LU) and brain metastases (BM) of 2 patients (V824 and V930), while there is currently no strong evidence that shows that *BRCA2* mutations are associated with CRC metastasis. In addition, the plotCNA function of MesKit can be used to characterize the CNA landscape across samples on the basis of copy number data. Consistent with TCGA projects and other previous studies of HCC [62, 63], a number of copy number alterations were observed in our HCC cohort, such as gains of 1q, 6p, 8q, and 13q, as well as losses of 1p, 4q, 9q, and 11q (Fig. 2B). Taken together, these data suggest that MesKit can easily characterize the mutational landscape and potential driver genes during cancer evolution.

### ITH estimation

**Figure 3:**
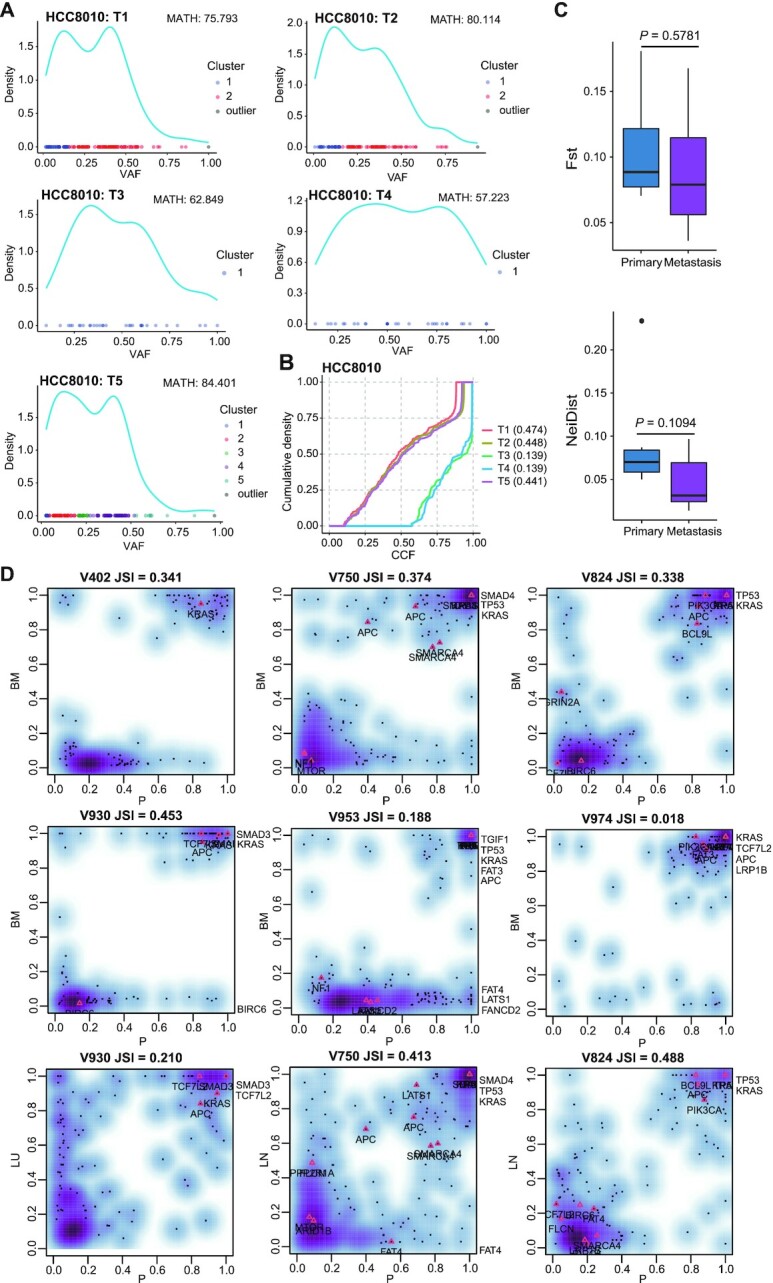
ITH estimation and the clonality of metastatic seeding **A**. Clustering mutations by VAFs of each tumor sample from HCC8010 based on a Gaussian finite mixture model. MATH scores are indicated above. **B**. CCF density plot of tumor samples from HCC8010. **C**. *F*_ST_- and Nei distance–based quantification of ITH in paired primary tumors and metastases of the CRC cohort (n = 7). *P*-value, Wilcoxon rank-sum test (2-sided). **D**. Density plots of merged CCF values in paired primary tumors and metastases of the CRC cohort. For each pair, the JSI was computed according to equation (7). Putative CRC driver genes are indicated on the plot. BM: brain metastasis; LN: lymph node metastasis; LU: lung metastasis; P: primary tumor.

Understanding the degree and development of ITH is clinically important because ITH has been associated with treatment resistance and the prognosis of patients with cancer [64]. MesKit integrates several approaches to estimate ITH within and between regions/tumors from the same patient. In MesKit, the mutCluster function deduces distinct subpopulations of a sample/tumor by clustering VAFs/CCFs in 1 dimension based on Gaussian finite mixture models [46]. It should be noted that low-frequency clusters might be a mixture of subclones that contain mutations coming from numerous parallel lineages growing neutrally [32, 65]. Another approach is calculating MATH score, which is positively correlated with tumor heterogeneity and metastatic potential [66, 67]. Besides, we integrated an index described by Charoentong et al. [48], to assess ITH by calculating the AUC of the cumulative density function from all CCFs per sample/tumor. Samples/tumors with higher AUCs are considered to be more heterogeneous than those with lower AUCs. Applying these measures on HCC8010 showed that samples with wider distributions of VAFs tended to have higher MATH scores, and VAF-based ITH was comparable to that calculated by CCFs (Fig. 3A and B). Moreover, we introduced 2 measures from population genetics [44, 49, 50], named *F*_ST_ and Nei genetic distance, to enable pairwise comparisons between regions/lesions. Comparison of ITH between primary tumors and paired metastases in the CRC cohort showed no significant difference using these 2 indices (Wilcoxon signed-rank test, *F*_ST_: *P* = 0.5781, Nei distance: *P* = 0.1094, Fig. 3C). Similarly, this observation supports the conclusion that primary and metastatic tumors of CRC exhibit a high degree of mutational discordance.

### Inferring the clonality of metastatic seeding

Because metastasis is the major cause of cancer-related death, it is particularly important to gain a systematic understanding of how tumor cells disseminate and the scale of ongoing parallel evolution in metastatic and primary sites [68]. Given that mutations with similar CCFs tend to cluster into the same subpopulation [69, 70], many studies inferred the potential metastatic routes between different lesions from the same patient by plotting CCFs of mutations [12, 42, 71]. By this means, Xue et al. [71] identified both monoclonal and multiclonal origins of separate type combined hepatocellular and intrahepatic cholangiocarcinoma (cHCC-ICC). Here, we developed the compareCCF function to calculate the merged CCFs of distinct lesions with MRS data. To visualize the seeding patterns between lesions in a more intuitive way, the results of this function can be further used to plot CCF plots, where the clusters at (1, 1) correspond to the clonal mutations present in all cells in both lesions (CCF = 1), while those on axes refer to lesion-private subclones. In addition, MesKit integrated a JSI-based method to calculate mutational similarity between lesions [42]. Pairs following polyclonal seeding generally achieve higher JSI values because of their higher proportion of shared subclonal sSNVs and fewer lesion-private sSNVs (Methods). Analysis of the CRC cohort with these functions revealed that all BMs exhibited enrichment of metastasis-private clonal sSNVs and shared clonal sSNVs but lacked shared subclonal sSNVs (Fig. 3D). Moreover, all BMs comprised a single phylogenetic clade in the phylogenetic trees (Supplementary Fig. S4). These observations jointly indicated that the BMs of this CRC cohort followed a monoclonal seeding manner, consistent with the original study [12]. Besides, in both paired primaries and metastases of most CRCs, the merged CCFs of mutations in CRC driver genes including *APC, KRAS*, and *TP53* were >0.6, suggesting that they may contribute to CRC tumorigenesis and metastasis. Notably, lymph nodes showed higher JSI values than distant metastases in V750 and V824, indicating that polyclonal seeding was more prevalent in lymph node metastases (Fig. 3D). In summary, these results demonstrated the ability and efficiency of MesKit to identify distinct patterns of seeding between paired lesions.

### Construction and visualization of phylogenetic trees

**Figure 4:**
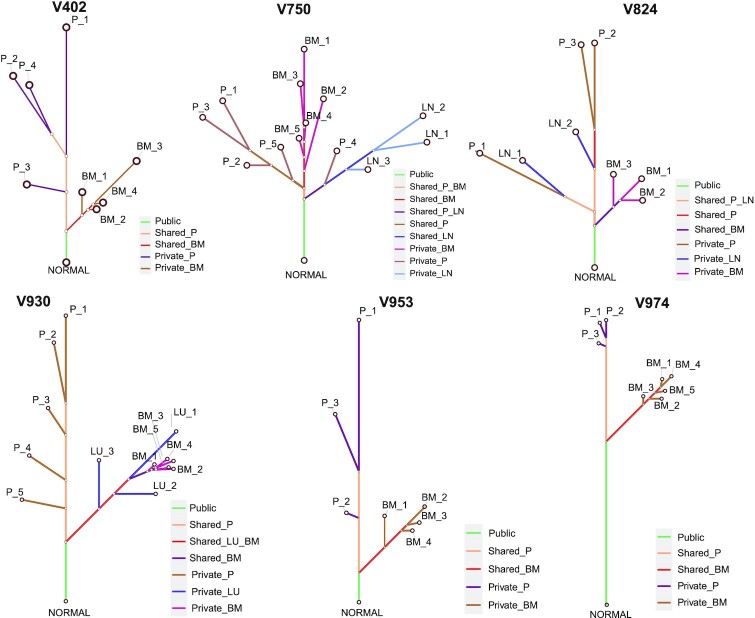
Phylogenetic trees of the CRC cohort Phylogenetic trees of the CRC cohort were constructed from all sSNVs and INDELs using the MP algorithm. Branches were colored according to the regional distribution of mutations. The branch lengths are proportional to the number of mutations.

A systematic understanding of the evolutionary relationships among tumor regions from a single patient plays a fundamental role in MRS studies, with the phylogenetic tree being a primary tool for delineating the relationship between tumor regions and interpreting ITH [2, 11, 44]. Consistent with original studies, we applied the MP method to reconstruct the tumor phylogeny of the CRC cohort using the getPhyloTree function in MesKit. Phylogenetic trees were further visualized with the function plotPhyloTree, which provides options to color the branches according to the classification of mutations or putative known signatures. We consistently reproduced tree structures of most CRCs from the original study [12], in which the primary regions and metastatic regions were clearly separated (Fig. 4). Inspection of the phylogeny indicated early divergence of the metastatic lineage in V402, V824, V930, V953, and V974, whereas divergence occurred during diversification of the primary tumor in V750. Moreover, we compared the MP-based phylogenetic trees with those constructed by the NJ method and ML method for each patient with CRC. Phylogenetic trees inferred through the 3 methods shared the same topology and clades for V402, V924, and V953 (Supplementary Fig. S3). When considering branch lengths, the MP-based trees were more similar to the NJ-based trees than the ML-based trees according to KF-branch distance [72] and weighted RF distance [74] (Supplementary Table S2). Collectively, these results demonstrate the functionality and efficiency of MesKit for analyzing and visualizing tumor phylogeny.

### Temporal dissection of mutational signatures

**Figure 5:**
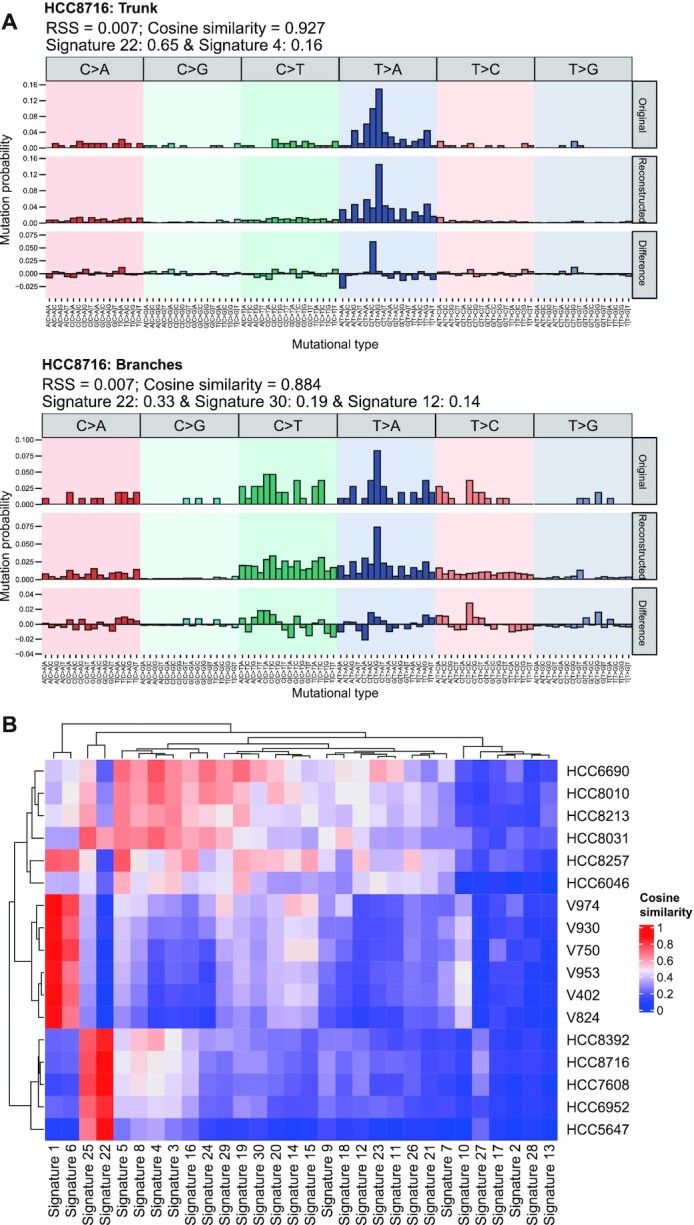
Temporal dissection of mutational signatures. **A**. Relative contribution of the 96 trinucleotide changes to the original mutational profile (upper panel), the reconstructed mutational profile (middle panel), and the difference between these profiles for truncal mutations and branch mutations from HCC patient HCC8716. The RSS, cosine similarity between the original and the reconstructed mutational profile and proposed etiology for mutational processes underlying the signature are indicated on the top. **B**. Heat map of cosine similarities between the 30 COSMIC signatures and the mutational profiles of the HCC and CRC cohorts. Patients were hierarchically clustered between the vectors of cosine similarities of signatures using the Euclidean distance methods. The signatures were ordered according to hierarchical clustering based on the cosine similarity between signatures.

Analysis of mutational signatures can be used to understand the mechanisms of transformation of normal cells to malignant cells and to identify underlying risk factors for tumor development. First, Alexandrov et al. [73] utilized >7,000 cancer genomes and exomes to identify 21 signatures across 30 tumor types. More recently, the Wellcome Trust Sanger Institute [78] published 30 mutational signatures (Version 2) in primary cancer and an expanded 67 single-base substitution signatures (Version 3). Considering the limited number of tumor samples assessed by MRS and thus the limited number of identified mutations, it is not amenable to conduct *de novo* signature extraction. Therefore, we developed the fitSignatures function to calculate the contribution of well-established signatures to mutations at different levels. By reconstructing the mutational profiles of the HCC and CRC cohorts using 30 COSMIC mutational signatures, we demonstrated that the signature contributions estimated by fitSignatures function were highly similar to those calculated by 3 other signature deconvolution tools (average Pearson correlation: 1, MutationalPatterns [23]; 0.997, SignatureEstimation [74]; 0.948, deconstructSigs [24]) (Supplementary Fig. S4A and Table S3). The similarities (indicated by cosine similarity) and discrepancies (indicated by RSS) between the original and reconstructed mutational profiles generated with MesKit were also comparable to those generated from other tools (Supplementary Figure S4B). As shown in Fig. 5B, hierarchical clustering via Euclidean distance of the patients based on their cosine similarity values clearly separated the HCCs from the CRCs. These results demonstrate the ability of MesKit to reliably estimate signature contributions. We further applied the fitSignatures function with 30 COSMIC signatures to truncal and branch sSNVs of HCC5647, HCC7608, and HCC8716 (other HCCs were excluded because their truncal/branch sSNVs were <50). All 3 HCCs exhibited a prominent decrease of the contribution of Signature 22 (exposures to aristolochic acid) in branch mutations compared with truncal mutations (Fig. 5A and Supplementary Table S4) Among them, HCC5647 and HCC8716 showed significantly higher percentages of T>A (*P* < 0.01) in truncal mutations than branch mutations (Supplementary Fig. S5), which is consistent with the characteristic patterns of Signature 22 (characterized by T>A). Considering these observations, we hypothesized that exposure to aristolochic acid contributed significantly to mutagenic process in the early stage of tumorigenesis for these HCCs. This analysis suggests the utility of MesKit to reveal the dynamic mutational processes.

## Discussion

Multi-region sequencing has become an affordable and effective way to investigate genetic heterogeneity and trace tumor evolutionary trajectory. Multiple spatial snapshots of tumors can help reduce sampling bias and detect minor subclones. Despite these advantages, there are few tools available to systematically analyze mutational data of multi-region samples from a single patient so far. In this regard, we present MesKit, an R/Bioconductor package, which incorporates a diversity of essential analysis and visualization functions for MRS studies. MesKit quantifies ITH based on somatic mutations by integrating several approaches described in recent cancer genome studies [47–50]. Besides, MesKit can be used to infer metastatic routes, characterize mutational patterns at different levels, and generate publication-quality images such as mutational profiles and phylogenetic trees. Via implementation of the Shiny application, MesKit enables researchers with minimal informatics skills to effortlessly interpret and visualize the intricate mutational data from MRS. Furthermore, we demonstrated the utility and efficiency of MesKit in interpreting ITH and inferring evolutionary trajectory using 2 published MRS datasets of HCC and CRC. Collectively, we believe that MesKit is a handy and feature-rich tool, which will greatly facilitate the exploration of mutational data from MRS experiments.

Because MesKit takes a MAF file and a clinical data file as standard inputs, it primarily evaluates ITH based on somatic mutations, and its assessment of contributions of CNAs is still limited. At present, several subclonal reconstruction methods are available to infer the relative order of occurrence between an SNV and its associated CNA. In future updates, we plan to implement the integration of results from these methods to provide insights into the clonality and temporal dynamics of ITH. On the other hand, as ITH arises through various mechanisms, it is invaluable to perform investigations at the genetic, transcriptomic, phenotypic, and cellular levels.

## Availability of Source Code and Requirements

Project name: MesKit

Project home page: https://github.com/Niinleslie/MesKit

Operating system(s): Platform independent

Programming language: R

Other requirements: R ≥4.0

License: GPL-3

RRID: SCR_020959

biotools: meskit

## Data Availability

The code for creating the figures in this article can be found and re-executed in a Code Ocean capsule [75]. Supporting data and an archival copy of the code are also available via the *GigaScience* database, GigaDB [76].

## Additional Files

**Figure S1**: Mutational landscape of HCC and CRC cohorts **A**. Mutational profile of HCC cohort. Oncoprint of top 15 most frequently mutated driver genes of HCC were grouped by public, shared, or private mutations including both clonal and subclonal drivers. Stacked bar charts on the top and right show the number of mutations for different types per sample and per driver gene, respectively. Genes were sorted by mutational frequency and samples were split by patients as indicated by the annotation bar (bottom). **B**. The consistent CNAs of CRC cohort with significant recurring CNAs identified from TCGA Colorectal Adenocarcinoma project by GISTIC2.0 (obtained from Broad GDAC website). Each track represents 1 tumor sample. BM: brain metastasis; LN: lymph node metastasis; LU: lung metastasis; P: primary tumor. Dark red indicates amplifications (CN ≥  4); light red, gains (2  <  CN  <  4); dark blue, deletions (CN  =  0); and light blue, losses (0  <  CN  <  2).

**Figure S2**: CCF heat maps of CRC cohort The heat maps of CCF values of tumor samples from the same patient. The color bar next to the heat map indicates the classification of mutations shared amongst different samples. The proportion of each classification is indicated in the legend. Putative CRC driver genes are labeled on the right.

**Figure S3**: Comparison of phylogenetic trees constructed by different methods of the CRC cohort. Comparison of the MP-based phylogenetic trees against those constructed by NJ method and ML method for each patient with CRC. For each pair, the different clades between 2 phylogenetic trees are highlighted in red (the first tree) or blue (the second tree).

**Figure S4**: Comparison of signature contributions measured by MesKit, MutationalPatterns, SignatureEstimation, and deconstructSigs. **A**. Relative contributions of all 30 COSMIC signatures for each patient in the HCC and CRC cohorts. **B**. Cosine similarity and RSS between the original and the reconstructed mutational profiles.

**Figure S5**: Mutation spectra of truncal and branch mutations of HCC5647, HCC7608, and HCC8716. Stacked bar plots show the proportions of truncal and branch mutations accounted for by each of the 6 mutation types in HCC5647, HCC7608, and HCC8716. The number of analyzed mutations is displayed on top of each bar. A Fisher exact test was used to compare truncal and branch mutations for each mutation type (2-sided test: *P < 0.01).

**Figure S6**: Schematic diagram of visualizing phylogenetic trees. Node *N* refers to a non-mutated normal sample: node 0 represents the starting node. In tree *T*_0_: *K* = {node 0, node 2, node 4, node 5, node 8}, ${K^{[ 1 ]}}$ is node 0; *B* = {node 1, node 3, node 6, node 7}, ${B^{[ 1 ]{\mathrm{\,\,}}}}$ is node 1; *R* = {node 1, node 7}, ${R^{[ 1 ]}}$ is node 1; L = {node 3, node 6}, ${L^{[ 1 ]}}$ is node 3.

**Table S1**: Clinical features of the HCC and CRC cohorts

**Table S2**: Distance between the MP-based phylogenetic tree and the NJ-/ML-based phylogenetic tree for each patient in CRC cohort

**Table S3**: Relative contributions of all 30 COSMIC signatures for each patient in HCC and CRC cohorts, as measured by MesKit, MutationalPatterns, SignatureEstimation, and deconstructSigs

**Table S4**: Signature contributions of truncal and branch mutations of HCC5647, HCC7608, and HCC8716

**Supplementary File S1**: Phylogenetic visualization auto-layout algorithm

## Abbreviations

AUC: area under the curve; BM: brain metastasis; CCF: cancer cell fraction; CI: confidence interval; CNAs: copy number alterations; COSMIC: Catalogue of Somatic Mutations in Cancer; CRC: colorectal cancer; *F*_ST_: fixation index; GDAC: Genome Data Analysis Center; GUI: graphical user interface; HCC: hepatocellular carcinoma; INDELs: small insertions and deletions; ITH: intra-tumor heterogeneity; JSI: Jaccard similarity index; LN: lymph node; LU: lung metastasis; MAD: median absolution deviation; MAF: mutation annotation format; MATH: mutant-allele tumor heterogeneity; ML: maximum likelihood; MP: maximum parsimony; MRS: multi-region sequencing; NJ: neighbor-joining; RSS: residual sum of squares; sSNV: somatic single-nucleotide variant; TCGA: The Cancer Genome Atlas; VAF: variant allele frequency; WES: whole-exome sequencing.

## Competing Interests

The authors declare that they have no competing interests.

## Authors’ Contributions

Q.Z. and J.R. conceived the project. M.L., J.C., X.W. and C.W. developed the methodology and implemented the method. X.Z., Z.Z. and Y.X. helped test the software. M.L., Q.Z., and J.R. wrote the manuscript. All authors read and approved the final manuscript.

## Supplementary Material

giab036_GIGA-D-21-00007_Original_Submission

giab036_GIGA-D-21-00007_Revision_1

giab036_Response_to_Reviewer_Comments_Original_Submission

giab036_Reviewer_1_Report_Original_Submission Roland Schwarz -- 1/31/2021 Reviewed

giab036_Reviewer_1_Report_Revision_1 Roland Schwarz -- 4/14/2021 Reviewed

giab036_Reviewer_2_Report_Original_Submission Theo Z Hirsch, Ph.D -- 2/2/2021 Reviewed

giab036_Reviewer_2_Report_Revision_1 Theo Z Hirsch, Ph.D -- 4/8/2021 Reviewed

giab036_Reviewer_3_Report_Original_Submission Marc Williams -- 2/4/2021 Reviewed

giab036_Reviewer_3_Report_Revision_1 Marc Williams -- 4/8/2021 Reviewed

giab036_Supplemental_Files
