# Supplementary material for: MesKit: a tool kit for dissecting cancer evolution of multi-region tumor biopsies through somatic alterations
Source: Gigascience. 2021 May 21;10(5):giab036. doi: 10.1093/gigascience/giab036 (PMC8138830; doi:10.1093/gigascience/giab036)
Supplement: giab036_GIGA-D-21-00007_Original_Submission [file giab036_giga-d-21-00007_original_submission.pdf]

GigaScience

MesKit: a tool kit for dissecting cancer evolution of multi-region tumor biopsies through somatic alterations

--Manuscript Draft--

**Manuscript Number:**	GIGA-D-21-00007
**Full Title:**	MesKit: a tool kit for dissecting cancer evolution of multi-region tumor biopsies through somatic alterations
**Article Type:**	Technical Note
**Funding Information:**	National Key R&D Program of China (2017YFA0106700) — Dr Jian Ren<br>Fundamental Research Funds for the Central Universities (SYSU: 19ykpy184) — Dr Qi Zhao
**Abstract:**	Background<br>Multi-region sequencing (MRS) has been widely used to analyze intra-tumor heterogeneity (ITH) and cancer evolution. However, comprehensive analysis of mutational data from MRS is still challenging, which requires complicated integration of a plethora of computational and statistical approaches.<br>Findings<br>Here, we present MesKit, an R/Bioconductor package, to assist in characterizing genetic ITH and tracing the evolutionary history of tumors based on somatic alterations detected by MRS. MesKit provides a wide range of analysis and visualization modules, including ITH evaluation, metastatic routes inference, and mutational signature identification. In addition, MesKit implements an auto-layout algorithm to generate phylogenetic trees based on somatic mutations. The application of MesKit on two reported MRS datasets of hepatocellular carcinoma (HCC) and colorectal cancer (CRC) reproduced known heterogeneous features, evolutionary patterns, together with potential driver events during cancer evolution.<br>Conclusions<br>In a word, MesKit is useful in interpreting ITH and tracing evolutionary trajectory based on MRS. MesKit is implemented in R and available at https://bioconductor.org/packages/MesKit under the GPL v3 License
**Corresponding Author:**	Qi Zhao<br>Sun Yat-Sen University Cancer Center<br>Guangzhou, Guangdong CHINA
**Corresponding Author Secondary Information:**	
**Corresponding Author's Institution:**	Sun Yat-Sen University Cancer Center
**Corresponding Author's Secondary Institution:**	
**First Author:**	Mengni Liu
**First Author Secondary Information:**	
**Order of Authors:**	Mengni Liu<br>Jianyu Chen<br>Xin Wang<br>Chengwei Wang<br>Xiaolong Zhang<br>Yubin Xie<br>Zhixiang Zuo<br>Jian Ren

Powered by Editorial Manager® and ProduXion Manager® from Aries Systems Corporation

	Qi Zhao
**Order of Authors Secondary Information:**	
**Additional Information:**	
**Question**	**Response**
Are you submitting this manuscript to a special series or article collection?	No
**Experimental design and statistics**<br><br>Full details of the experimental design and statistical methods used should be given in the Methods section, as detailed in our Minimum Standards Reporting Checklist. Information essential to interpreting the data presented should be made available in the figure legends.<br><br>Have you included all the information requested in your manuscript?	Yes
**Resources**<br><br>A description of all resources used, including antibodies, cell lines, animals and software tools, with enough information to allow them to be uniquely identified, should be included in the Methods section. Authors are strongly encouraged to cite Research Resource Identifiers (RRIDs) for antibodies, model organisms and tools, where possible.<br><br>Have you included the information requested as detailed in our Minimum Standards Reporting Checklist?	Yes
**Availability of data and materials**<br><br>All datasets and code on which the conclusions of the paper rely must be either included in your submission or deposited in publicly available repositories (where available and ethically appropriate), referencing such data using a unique identifier in the references and in	Yes

Powered by Editorial Manager® and ProduXion Manager® from Aries Systems Corporation

the "Availability of Data and Materials" section of your manuscript.<br><br>Have you have met the above requirement as detailed in our Minimum Standards Reporting Checklist?	

Powered by Editorial Manager® and ProduXion Manager® from Aries Systems Corporation

Manuscript

Click here to access/download;Manuscript;MesKit_manuscript_Gigasciense.

MesKit: a tool kit for dissecting cancer evolution of multi-region tumor biopsies through somatic alterations

Mengni Liu[1,#], Jianyu Chen[1,#], Xin Wang[1], Chengwei Wang[1], Xiaolong Zhang[1], Yubin Xie[1], Zhixiang Zuo[1], Jian Ren1[1,*], Qi Zhao[1,*]

[1]*State Key Laboratory of Oncology in South China, Collaborative Innovation Center for Cancer Medicine, Sun Yat-sen University Cancer Center, 651 E Dongfeng Road, Guangzhou, Guangdong, 510060, China*

[#] Equal contribution.

[*] Corresponding authors.

E-mail: zhaoqi@sysucc.org.cn (Qi Z) or renjian@sysucc.org.cn (Jian R)

Abstract

Background

Multi-region sequencing (MRS) has been widely used to analyze intra-tumor heterogeneity (ITH) and cancer evolution. However, comprehensive analysis of mutational data from MRS is still challenging, which requires complicated integration of a plethora of computational and statistical approaches.

Findings

Here, we present MesKit, an R/Bioconductor package, to assist in characterizing genetic ITH and tracing the evolutionary history of tumors based on somatic alterations detected by MRS. MesKit provides a wide range of analysis and visualization modules, including ITH evaluation, metastatic routes inference, and mutational signature identification. In addition, MesKit implements an auto-layout algorithm to generate phylogenetic trees based on somatic mutations. The application of MesKit on two reported MRS datasets of hepatocellular carcinoma (HCC) and colorectal cancer (CRC) reproduced known heterogeneous features, evolutionary patterns, together with potential driver events during cancer evolution.

Conclusions

In a word, MesKit is useful in interpreting ITH and tracing evolutionary trajectory based on MRS. MesKit is implemented in R and available at https://bioconductor.org/packages/MesKit under the GPL v3 License.

Keywords: multi-region sequencing; somatic alterations; intra-tumor heterogeneity; metastatic routes; phylogenetic tree

Introduction

Cancer evolves through a process of somatic alterations [1], of which the spatially and/or temporally changes could be detected by multi-region sequencing (MRS). Nowadays, MRS has become an effective and affordable way to trace evolutionary history of carcinogenesis and metastasis. The cancer evolution research has focused on identification and estimation of ITH, phylogeny reconstruction, and mutational signature analysis, etc. Numerous MRS studies have identified extensive ITH among many solid tumors originated in liver, prostate, esophagus, breast, and lung [2-7]. In addition, higher ITH has been implicated in dismal cancer prognosis [8-10]. While recent studies largely generated descriptive summaries on ITH, quantitative interpretation of heterogeneity within and between tumors from the same patient is more informative in personal therapeutics.

And recently, plenty of MRS studies have employed phylogenetic trees to show the temporal sequence and heterogeneous divergence between the samples [2, 11, 12]. There are also increasing efforts to reconstruct subclonal phylogenies via 'clone tree', which summarizes lineage relationships between cellular subpopulations[13-15]. Phylogeny reconstruction over the cancer cell fraction (CCF) estimates has identified both monoclonal and multiclonal seeding patterns in several cancers [3, 16-18]. The distinction between these two patterns may have important clinical implications [19], thus, it is necessary to infer metastatic routes and explore the potential metastasis drivers.

Moreover, MRS provides insight into the dynamics of mutational processes during tumor progression. A previous study has indicated that DNA damage repair dysfunction might be crucial for mutation accumulation during osteosarcoma evolution [20]. Recently, Yan et al. [21] applied MRS on 39 esophageal squamous cell carcinoma (ESCC) patients and identified several potential actionable targets such as *EGFR* and *FGFR1*. They also shown that APOBEC mutations and aging predominated in early stage of tumorigenesis of ESCC. These findings suggest that MRS strategy has the potential to reveal mutational mechanisms and thereby could improve both diagnosis

and treatment.

Several tools are available for mutational analysis. For example, Maftools [22] provides multiple functions involving pathway annotation, *de novo* signature, and enrichment analysis, etc. MutationalPatterns [23] and deconstructSigs [24] are powerful tools to explore mutational patterns and identify mutational signatures of a single tumor sample. And LICHeE [25] and SCHISM [26] were developed to reconstruct multi-sample tumor phylogenetic trees and infer subclonal composition within samples. However, performing integrated mutational analysis with these tools require inconsistent input formats. Besides, it can be time-consuming and tedious to generate publication-quality images such as mutational profiles and phylogenetic trees, which requires manual modification using other graphic editors. To our knowledge, no one-stop platform is available that offers comprehensive analysis and visualization for MRS dataset yet.

Accordingly, we present MesKit, an R/Bioconductor package, to provide commonly used analysis and visualization modules for MRS studies. MesKit is capable of depicting mutational profiles, measuring heterogeneity within or between tumors from the same patient, tracking evolutionary dynamics, as well as characterizing mutational patterns on different levels. In addition, we implemented an auto-layout algorithm to visualize rooted phylogenetic trees with annotations. To demonstrate the application and performance of MesKit, we utilized two high-quality MRS datasets of hepatocellular carcinoma (HCC) [2] and colorectal cancer (CRC) [12], both of which contain somatic mutations as well as CNAs (Table S1). We reproduced well-known heterogeneous features, evolutionary patterns, together with potential driver events of HCC and CRC, demonstrating the robustness of MesKit to interpret ITH and infer evolutionary trajectory based on MRS.

Materials and methods

Data collection and preprocessing

The cohort of HCC includes tumor tissues (n = 52) and matched blood (Germline, n = 11) samples from 11 patients, which were collected before treatment [2]. Each sample was sequenced using whole exome sequencing (WES) to high coverage (range, 72X–232X) and re-analyzed with the same workflow described as below. In brief, we performed variant calling by Mutect (version 1.1.7) [27] and filtered the detected mutations with normal depth < 8 or tumor depth < 8 for each sample. Additionally, we adopt "force calling" method [28] to rescue potential real mutations for each sample based on the aggregate set of somatic events in each patient using *samtools mpileup* (version 1.2) [29]. Mutations with variant allele frequencies (VAFs) less than 0.04 were discarded. The CCFs were estimated by PyClone (version 0.13.0) [30], which adjusted the VAFs of somatic mutations based on local copy numbers of the mutated loci (FreeC v11.0) [31] and tumor purity (FACETS v0.5.14) [32]. CRC cohort consisted of six patients who were performed with MRS of paired primary tumors and metastases (3–5 regions each) [12]. Somatic alterations including somatic single-nucleotide variants (sSNVs), small insertions and deletions (indels) or copy-number alterations (CNAs) and mutational CCF of CRCs were obtained from the original study. Driver genes of HCC and CRC were defined by IntOGen (v.2020.2), and GISTIC2 results of TCGA HCC project and TCGA CRC project were obtained from Broad GDAC website (analysis stamp: 2016_01_28).

Clonal status of somatic mutations

For tumors with MRS data, the merged allele frequency and CCF of each mutation was computed from multiple regions as previously used [12, 33, 34]:

$$VAF_{merged} = \frac{\sum_{i=1}^{k} VAF_i \times d_i}{\sum_{i=1}^{k} d_i} \quad (1)$$

$$CCF_{merged} = \begin{cases} \frac{\sum_{i=1}^{k} CCF_i \times d_i}{\sum_{i=1}^{k} d_i} & CCF < 1 \\ 1 & CCF \geq 1 \end{cases} \quad (2)$$

where d_i, VAF_i and CCF_i refer to the sequencing depth, VAF estimation and CCF estimation in region i, respectively. The clonal and subclonal sSNVs/indels were determined based on CCF. A CCF value of 1 corresponds to a mutation present in 100% of the cancer cells in a sample, while a CCF value < 1 indicates that the mutation is present in a subset of the cancer cells in a sample and thus is subclonal. In each sample, a mutation m was classified as clonal if the upper bound of the 95% confidence interval (CI) of $CCF_m \geq 1$, and otherwise subclonal [35]. When MRS data is available, a mutation m is considered as subclonal when all of the following criteria are satisfied: (1) At least one region with the upper bound of 95% CI of $CCF_m < 1$; (2) At least one region with $CCF_m < 0.5$; (3) The CCF_{merged} of mutation m < 0.5, by which the cut-off is used for its good performance in defining subclonality based on simulated virtual tumors [36, 37];

Estimation of ITH

To quantify the genetic divergence of ITH between regions or tumors, we introduced two classical metrics derived from population genetics, which were Wright's fixation index (Fst) and Nei's genetic distance [38, 39]. Between-region genetic heterogeneity within tumors only keeps subclonal mutations in calculation, as clonal mutations present in all regions do not contribute to ITH. For pairwise comparison of heterogeneity between tumors, both clonal and subclonal mutations are taken into consideration. Fst index estimating between-region ITH for k regions was computed as follows:

$$FST = \frac{1}{r} \times \sum_{j=1}^{r} FST_j^{Hudson}, \ r = \binom{2}{k} \tag{3}$$

$$Fst_j^{Hudson} = \frac{\sum_{m=1}^{mt} (f_a^m - f_b^m)^2 \frac{f_a^m \times (1 - f_a^m)}{d_a^m - 1} - \frac{f_b^m \times (1 - f_b^m)}{d_b^m - 1}}{\sum_{m=1}^{mt} f_a^m \times (1 - f_b^m) + f_b^m \times (1 - f_a^m)} \tag{4}$$

where f_a^m denotes the VAF for sSNV m and d_a^m denotes the sequencing depth for sSNV m in region a.

And Nei's genetic distance for k regions within the same tumor was defined as follows [39]:

$$D_{Nei} = \frac{1}{r} \times \sum_{j=1}^{r} D_{Nei_j}, \; r = \binom{2}{k} \tag{5}$$

$$D_{Nei_j} = -log \frac{\sum_{m=1}^{mt} ccf_a^m \times ccf_b^m + (1- ccf_a^m)(1 - ccf_b^m)}{\sqrt{(\sum_{m=1}^{mt} ccf_a^{m2} + (1- ccf_a^m)^2) \times (\sum_{m=1}^{mt} ccf_b^{m2} + (1- ccf_b^m)^2)}} \tag{6}$$

where ccf_a^m and ccf_b^m represent the CCF values in region a and region b for mutation m.

MesKit also includes several measures of ITH defined by recent genomic researches. Mroz et al. [40] developed the mutant-allele tumor heterogeneity (MATH) index, which corresponds to the ratio of the median absolution deviation (MAD) and the median of the VAF values among tumor-specific mutated loci. Generally, a more heterogeneous tumor with a higher MATH score would tend to have a wider distribution of VAF among all mutation loci, and centered at a lower fraction.

$$MATH = 1.4268 \times \frac{MAD(VAF_{avg})}{Median(VAF_{avg})} \tag{7}$$

Another approach to estimates ITH is calculating the area under the curve (AUC) of the cumulative density function based on CCF per tumor, and tumors with higher AUC are considered to be more heterogeneous [41].

Inference of metastatic routes

For spatially separated regions from the same patient, the potential metastatic route can be determined by comparing subclonal architecture between paired regions. Given that mutations with similar CCF across samples tend to cluster into the same subpopulation [42, 43], we developed the *compareCCF* function, which identifies subclones shared between different regions via CCF plots [12, 34, 44]. The clusters at (1, 1) correspond to the clonal mutations present in all the cells in both regions or tumors (CCF = 1), while those on axes refer to region-specific subclones. MesKit also integrated a Jaccard similarity index (JSI) based method to identify metastatic seeding patterns of distant metastases or lymph node metastases [34]. The Jaccard coefficient for a sample pair (a, b) was calculated as follows:

$$JSI = \frac{SS_{ab}}{PC_a + PC_b + SS_{ab}} \tag{7}$$

where SS_{ab}, PC_a/PC_b represent shared subclonal sSNVs for sample pair (a, b), and private clonal sSNVs of sample a/b, respectively. The mean SS_{ab}, PC_a/PC_b of all sample pairs from tumor a and tumor b were used to compute the JSI for tumors with MRS data. Additionally, the JSI value for each individual is calculated by the mean SS_{ab}, PC_a and PC_b, which is another measurement of the intra-tumor heterogeneity between different tumor regions.

Construction and visualization of phylogenetic trees

With MesKit, phylogeny of multiple specimens from individual patients were reconstructed based on the presence or absence of somatic mutations. This process was implemented in function *getPhyloTree* by utilizing R implementations of several standard phylogenetic approaches from APE[45] and PHANGORN [46] R packages, including distance-based methods, such as neighbor-joining[47] and minimum evolution[48], character-based methods like maximum parsimony[49] and maximum likelihood[50]. Furthermore, MesKit measures similarity between two phylogenetic trees using *treedist* function from PHANGORN [46] R package. Notably, we implemented an auto-layout algorithm via function *plotPhyloTree* to generate customizable images of phylogenetic trees with annotations (File S1).

Mutational signature analysis

To illustrate the dynamic mutational spectrum during tumor progression, we implemented mutational signature analysis based on phylogenetic trees. The process starts with constructing a mutation matrix accounts for 96 trinucleotide changes, where the sequence context of the base substitutions can be retrieved from the corresponding reference genome using BSgenome R package. In the meantime, 6 types of base substitution types are distinguished by convention: C>A, C>G, C>T, T>A, T>C, T>G. As methylated cytosine at CpG sites with the attendant risk of spontaneous deamination are mutagenic hotspots in human genome[51], C>T mutations can be divided into C>T at CpG sites and other sites [23]. Genomic mutations were temporally dissected into truncal (shared among all samples from the same patient) and branch mutations of

phylogenetic trees. For each mutational type, Fisher's exact test was implemented to assess the difference between the truncal and branch mutations. Once the signature matrix was provided, we can calculate the contribution of known signatures to the mutational profile of different groups of mutations via *fitSignatures* function. For convenience, we included known signature matrix (published by Alexandrov et al. in 2013 and Cosmic version 2, 3) along with proposed etiology in MesKit. As previously described [23], we considered it as a non-negative least-squares (NNLS) constraints problem and assigned weight to each signature in order to best reconstruct the mutational profile using pracma R package (https://CRAN-project.rg/package=-pracma). We measured similarity between mutational profiles A and B by calculating cosine similarity as follows:

$$\mathrm{sim}(A,B) = \alpha = \frac{\sum_{i=1}^{n} A_i B_i}{\sqrt{\sum_{i=1}^{n} A_i^2}\sqrt{\sum_{i=1}^{n} B_i^2}} \tag{8}$$

where mutational profiles A, B are non-zero vectors with n mutational types. Two mutational profiles are identical when the cosine similarity is 1, which are independent when the cosine similarity is 0 conversely.

Results

Overview of MesKit functions and implementation

MesKit was implemented as an open source R/Bioconductor package, which integrates commonly used analysis and visualization methods to interpret mutational data from MRS experiments. It takes Mutation Annotation Format (MAF) file of somatic mutations as input, with cancer cell fraction estimates as an optional dataset. Briefly, MesKit provides a series of analysis and visualization functions, including characterizing mutational landscape, estimating ITH, inferring metastatic routes, exploring mutational patterns, as well as visualizing phylogenetic trees automatically (**Figure 1**). In addition, to facilitate the usage of the package, we implemented a Shiny application embedded in the package for a need of interactive analysis. Moreover, we

built a Docker image that enables the deployment Shiny-based MesKit GUI in a C/S mode.

Mutational landscape from MRS studies

Generally, somatic mutations identified from MRS in a single tumor were usually classified into "public mutations" (exist in all regions of the tumor), "shared mutations" (exist in part of all regions), and "private mutations" (exist in a single region) [20, 37, 52]. Such spatial-mutation categories largely correspond to the temporal order of their genesis during the tumor evolution: most public mutations occur early in tumor-initiating cells and are inherited by their offsprings, whereas private mutations accumulate sporadically and markedly increase the ITH among different patients [53]. In Meskit, we implemented the function *classifyMut* to help categorize somatic mutations based on regional distribution, or to identify clonal and subclonal mutations according to their estimated CCFs (Methods). Analysis of HCC and CRC cohorts showed significant inter-individual heterogeneity but much less intra-individual heterogeneity (**Figure 2A, B** and Figure S1A, B). In line with previous findings [13, 54, 55], primary tumors and metastases of CRC cohort exhibited high genomic concordance (Figure 2A). As expected, public mutations harbored higher CCF compared to private mutations (Figure S2), which were more likely to be clonal events. The recurrent mutations in putative driver genes of CRCs (defined by IntOGen v.2020.2), such as *KRAS, APC*, were clonal and shared between paired primary tumors and metastases, indicating their early occurrence in colorectal carcinogenesis (Figure S1, S2). Interestingly, *BRCA2* mutation was private to distant metastases, including lung metastasis (LU) and brain metastasis (BM) of two patients (V824 and V930), suggesting its metastasis-promoting ability in CRC cells. Besides, the *plotCNA* function of MesKit characterizes the CNA landscape across samples based on copy number data. Consistent with TCGA projects and other previous studies of hepatocellular carcinoma [56, 57], a number of copy number changes were observed in our HCC cohort, such as gains on 1q, 6p, 8q and losses on 4q, 9q, 11q, 16q (Figure 2B). Taken together, MesKit

can easily characterize the mutational landscape and potential driver genes during cancer evolution.

ITH estimation

Understanding the degree and development of ITH is clinically important, which has been associated with treatment resistance and prognosis of cancer patients [58]. MesKit integrated several metrics to estimate ITH within or between regions/tumors from the same patient. It is common to leverage the VAFs of somatic mutations to distinguish their origin of subpopulations. In MesKit, the *vafCluster* function deduces the number and proportion of distinct subpopulations by clustering VAFs in samples based on a Gaussian finite mixture model [59]. We also introduced an ITH indice by calculating the AUC of the cumulative density function from all CCFs per sample/tumor as previous described [41], where samples/tumors with higher AUC were considered to be more heterogeneous. Another approach of measuring heterogeneity, MATH score, is positively correlated with tumor heterogeneity and metastatic potential [60, 61]. Analysis of HCC patient HCC8010 showed that samples with a wider distribution of VAF tended to have higher MATH scores and AUC values of CCFs (**Figure 3A**, B). Moreover, we introduced two measures from population genetics [37-39], named Fst and Nei's genetic distance, enabling pairwise comparisons between regions or tumors. Utilizing these two indices to quantify the ITH within tumors of CRCs separately, no significantly differences of ITH were observed between primary tumors and paired metastases (Wilcoxon signed-rank test, Fst: P = 0.5781, Nei's distance: P = 0.1094, Figure 3C), which was consistent with rapid genetic diversification.

The clonality of metastatic seeding

Since metastasis is the major cause of cancer-related death, it is particularly important to gain a systematic understanding of how tumor cells disseminate and the scale of ongoing parallel evolution in metastatic and primary site [62]. Several studies have reported that metastases (including lymph node metastases and distant metastases) were seeded in a monoclonal [16, 17] or polyclonal manner [3, 4] from primary tumor or

other metastases. To deduce the potential metastatic route between separate regions, CCFs of mutations were plotted between paired regions. In the reanalysis of CRC cohort, we observed the patterns of seeding based on merged CCF between pairs of primary tumor and metastases. We found that all brain metastases exhibited enrichment of metastasis-private clonal mutations and shared clonal sSNVs, but lacked shared subclonal sSNVs (Figure 3D). These results demonstrated that the brain metastases of this CRC cohort followed a monoclonal seeding manner, consistent with the original study [12]. Moreover, all brain metastases of CRCs comprised a single phylogenetic clade in phylogenetic trees (Figure S3), which is associated with monoclonal seeding. The merged CCFs of *APC*, *KRAS* and *TP53* mutations were > 0.6 in both paired primaries and metastases of most CRCs, suggesting they may contribute to CRC tumorigenesis and metastasis. In addition, Meskit integrated a JSI-based method to calculate mutational similarity between primary tumor/metastasis pairs [34]. Those pairs which follows polyclonal seeding generally gains a higher JSI value, due to their higher proportion of shared subclonal sSNVs and fewer metastasis- or primary tumor-private sSNVs (Methods). Notably, lymph nodes showed higher JSI than distant metastases in f V750 and V824, indicating polyclonal seeding was more prevalent in lymph node metastases (Figure 3E).

Temporal dissection of mutational signatures

The analysis of mutational signatures could be used to understand the mechanisms of transformation of normal cells into malignant cells and to identify underlying risk factors for tumour development. Firstly, Alexandrov et al. [63] utilized over 7, 000 cancer genomes and exomes to identify 21 signatures across 30 tumor types. More recently, the Wellcome Trust Sanger Institute (http://cancer.sanger.ac.uk/cosmic/signatures) published 30 mutational signatures (version 2) in primary cancer and an expanded 67 single base substitution signatures (version 3). Considering the limited number of tumor samples and sSNVs of MRS, it is not amenable to conduct *de novo* signature extraction. Therefore, we developed the *fitSignatures* function to calculate the contribution of well-established signatures to individual tumors. By fitting the mutational profile of public sSNVs of HCCs to

COSMIC version 2 signatures, we found that signature 22 (exposures to aristolochic acid) and signature 4 (associated with tobacco mutagens) weighted relatively high in the reconstructed mutational profiles. And we identified dominant signature 1 (associated with age) and signature 12 in private sSNVs of 8 HCCs (Figure 4A, File S2). Signature 12 was so far described exclusively in liver cancers. Considering these observations, we hypothesized that exposure to aristolochic acid and tobacco consumption jointly contribute to mutagenic process in the early stage of tumorigenesis for this HCC cohort. Cosine similarity value can be utilized to test how well each mutational profile can be explained by the provided mutational signatures (Figure 4A) or to compare different mutational profiles. As shown in Figure 4B, hierarchical clustering of the patients between their cosine similarity values based on the Euclidean distance clearly separates the HCCs from CRCs. This analysis demonstrates the utility of MesKit to reveal the dynamic mutational processes, enabling comparisons of distinct mutational profiles.

Construction and visualization of phylogenetic trees

Systematic understanding of evolutionary relationship among tumor samples from a single patient plays a fundamental role in MRS study, where phylogenetic tree is a primary tool for delineating the relationship between tumor samples and interpreting ITH [2, 11, 37]. Consistent with the original studies, we applied maximum parsimony method to reconstruct tumor phylogeny of HCC and CRC cohorts using the *getPhyloTree* function in MesKit. The visualization of phylogenetic trees was implemented in the function *plotPhyloTree,* which provides options to color the branches according to classification of mutations or putative known signatures. Applying these functions on CRC cohort, we reproduced the consistent tree structures of most CRCs with the original study [12], in which the primary regions and metastatic regions of were clearly separated (**Figure 5A**, S3). Inspection of the phylogeny indicated early divergence of metastatic lineage in V402, V824, V930, V953, V974, whereas divergence occurred during diversification of the primary tumor in V750. Furthermore, a significant higher percentage of T>A ($P < 0.05$) of trunk mutations was

observed in 3 HCCs (HCC5647, HCC7608, HCC8716), which is consistent with the characteristic pattern of the dominated signature 22 identified by cosine similarity estimates (Methods, Figure 5B). Collectively, these results demonstrate the functionality and efficiency of MesKit to analyze and visualize the tumor phylogeny.

Discussion

Multi-region sequencing has become an affordable and effective way to investigate genetic heterogeneity and subclonal construction, as well as to trace tumor evolutionary trajectory. Multiple temporal and spatial snapshots of tumors can help reduce sampling bias and detect minor subclones which may result in unexpected therapeutic resistance. Despite these advantages, few software is available to systematically analyze mutational data of multi-region samples from a single patient so far. In this regard, we present MesKit, an R/Bioconductor package, which incorporates a diversity of essential analysis and visualization functions for MRS studies. In addition, via implementation of Shiny application, MesKit enables researchers with minimal informatics skills to interpret and visualize the intricate mutational data from MRS effortlessly. Here, we reproduced cancer evolutionary analysis using two published mutational datasets of HCC and CRC. Practically, ITH arises through various mechanisms, therefore, it is necessary to perform investigation at the genetic, transcriptomic, phenotypic, and cellular levels. In future updates, we will include gene expression as well as DNA methylation data for integrative multi-omics analysis.

Availability of source code and requirements

Project name: MesKit

Project home page: https://github.com/Niinleslie/MesKit

Operating system(s): Platform independent

Programming language: R

Other requirements: R ≥ 4.0

License: GPL-3

The code for creating the figures in this article can be found and re-executed in a Code Ocean capsule[64].

Abbreviations

AUC: area under the curve; BM: brain metastasis; CCF: cancer cell fraction; CI: confidence interval; CNAs: copy-number alterations; COSMIC: catalogue of somatic mutations in cancer; CRC: colorectal cancer; ESCC: esophageal squamous cell carcinoma; Fst: fixation index; HCC: hepatocellular carcinoma; indels: small insertions and deletions; JSI: jaccard similarity index; LN: lymph node; LU: lung metastasis; MAD: median absolution deviation; MAF: mutation annotation format; MATH: mutant-allele tumor heterogeneity; MRS: multi-region sequencing; NNLS: non-negative least squares; RSS: residual sum of squares; sSNVs: somatic single-nucleotide variants; VAF: variant allele frequency; WES: whole exome sequencing

Authors' contributions

QZ and JR conceived the project. ML, JC, XW, and CW developed the methodology and implemented the method. LZ and YX helped test the software. ML, QZ and JR wrote the paper. All authors read and approved the final manuscript.

Competing interests

The authors have declared no competing interests.

Acknowledgements

This work was supported by grants from the National Natural Science Foundation of China (Grant Nos. 91753137, 31471252, 31771462, 81772614, U1611261 and 31801105); National Key R&D Program of China (Grant No. 2017YFA0106700); Program for Guangdong Introducing Innovative and Entrepreneurial Teams (Grant No. 2017ZT07S096); Guangdong Natural Science Foundation (Grant No. 2018A030313323); and Fundamental Research Funds for the Central Universities (SYSU: 19ykpy184).

Figure legends

Figure 1 Overview of MesKit package

A. Overview of MesKit. MesKit consists of five major modules, including characterizing mutational landscape, estimating ITH, inferring metastatic routes, exploring mutational patterns, as well as visualizing phylogenetic trees automatically. We display the corresponding functions for each module separately. The whole workflow is built based on Maf objects and phyloTree objects, which are taken as inputs for most functions.

Figure 2 Mutational landscape of HCC and CRC cohorts

A. Mutational profile of CRC cohort. Oncoprint of top 15 most frequently mutated driver genes of CRC are grouped by public, shared or private mutations including both clonal and subclonal drivers. Genes are sorted by mutational frequency and those with multiple mutations are annotated as Multi_Hit. Samples are split according to patients as indicated by the annotation bar (bottom). P, primary tumor; BM, brain metastasis; LN, lymph node metastasis; LU, lung metastasis. Stacked bar charts on the top and right show the number of mutations for different types per sample and per driver gene, respectively. **B**. The consistent CNAs of HCC cohort with significant recurring CNAs identified from TCGA hepatocellular carcinoma project by GISTIC2.0 (obtained from Broad GDAC website). Each track represents one tumor sample. Dark red for amplifications ($CN \geq 4$), light red for gains ($2 < CN < 4$), dark blue for deletions ($CN = 0$), and light blue for losses ($0 < CN < 2$).

Figure 3 ITH estimation and the clonality of metastatic seeding

A. Cluster VAFs of each tumor sample and merged VAFs of the whole tumor from HCC8010 based on a Gaussian finite mixture model. MATH scores are indicated above. **B**. CCF density plot of tumor samples from HCC8010. **C**. Fst- and Nei's distance-based quantification of ITH in paired primary tumors and metastases of CRC cohort (n = 7). P-value, Wilcoxon Rank-Sum Test (two-sided). **D**. Density plot of

merged CCF estimates in paired primary CRCs and metastases. For each primary tumor/metastasis sample pair, the JSI was computed according to equation (7). Putative CRC driver genes are indicated on the plot. P, primary tumor; BM, brain metastasis; LN, lymph node metastasis; LU, lung metastasis.

Figure 4 Temporal dissection of mutational signatures

Relative contribution of the 96 trinucleotide changes to the original mutational profile (upper panel), the reconstructed mutational profile (middle panel), and the difference between these profiles for public mutations (**A**) and private mutations (**B**) from HCC patient HCC6952. The residual sum of squares (RSS), the cosine similarity between the original and the reconstructed mutational profile and proposed etiology for the mutational processes underlying the signature are indicated on the top. **C**. Heatmap of cosine similarities between the mutational profile of each patient in HCC and CRC cohorts and COSMIC signatures. The patients are hierarchically clustered between the vectors of cosine similarities of signatures using the Euclidean distance. The signatures are ordered according to hierarchical clustering based on the cosine similarity between signatures.

Figure 5 Visualization of Phylogenetic trees

A. The phylogenetic tree of CRC patient V402. The phylogenetic tree is constructed from all sSNVs and indels using maximum parsimony algorithm. The branch lengths are proportional to the number of mutations. Branches are colored according to regional distribution of mutations, which are public (green), primary-shared (orange), BM-shared (red), primary-private (purple) and BM-private (brown). **B**. Phylogenetic trees of HCC patient HCC5647, HCC7608 and HCC8716 are constructed from sSNVs using maximum parsimony algorithm. For each branch, the length is proportional to the number of mutations, and the color indicates the best possible matched COSMIC mutational signatures of its mutations by calculating cosine similarity values. Branches with mutations fewer than 15 are marked as “Unknown”. For each patient, comparison of mutation spectrums between truncal mutations versus branch mutations is displayed

on the right of the tree. P-values are derived by Fisher’s exact test (Two-sided: *P < 0.05, **P < 0.01). The number of mutations is indicated on top of the bar.

Supplementary material

Figure S1 Mutational landscape of HCC and CRC cohorts

A. Mutational profile of HCC cohort. Oncoprint of top 15 most frequently mutated driver genes of HCC are grouped by public, shared or private mutations including both clonal and subclonal drivers. Stacked bar charts on the top and right show the number of mutations for different types per sample and per driver gene, respectively. Genes are sorted by mutational frequency and samples are split according to patients as indicated by the annotation bar (bottom). **B**. The consistent CNAs of CRC cohort with significant recurring CNAs identified from TCGA Colorectal Adenocarcinoma project by GISTIC2.0 (obtained from Broad GDAC website). Each track represents one tumor sample. P, primary tumor; BM, brain metastasis; LN, lymph node metastasis; LU, lung metastasis. Dark red for amplifications ($CN \geq 4$), light red for gains ($2 < CN < 4$), dark blue for deletions ($CN = 0$), and light blue for losses ($0 < CN < 2$).

Figure S2 CCF heatmaps of CRC cohort

The heat map shows the CCF values of mutations in tumor samples from the same patient. The color bar next to the heatmap indicates the classification of mutations shared amongst different samples. The proportion of each classification is indicated in the legend. Putative CRC driver genes are labelled on the right.

Figure S3 Phylogenetic trees of CRC cohort

Phylogenetic trees of CRCs are constructed from all sSNVs and indels using maximum parsimony algorithm. The branch lengths are proportional to the number of mutations. And branches are colored according to regional distribution of mutations.

Figure S4 Schematic diagram of visualizing phylogenetic trees

Node N refers to a non-mutated normal sample: node 0 represents the starting node. In tree T_0: $K = \{node\ 0, node\ 2, node\ 4, node\ 5, node\ 8\}$, $K^{[1]}$ is $node\ 0$;

$B = \{node\ 1,\ node\ 3,\ node\ 6,\ node\ 7\}$, $B^{[1]}$ is $node\ 1$; $R = \{node\ 1,\ node\ 7\}$, $R^{[1]}$ is $node\ 1$; $L = \{node\ 3, node\ 6\}$, $L^{[1]}$ is $node\ 3$

Table S1 Clinical features of the HCC cohort and CRC cohort
Table S2 Mutational signatures of HCC cohort

References

1. Hanahan D and Weinberg RA. Hallmarks of cancer: the next generation. Cell. 2011;144 5:646-74. doi:10.1016/j.cell.2011.02.013.
2. Lin DC, Mayakonda A, Dinh HQ, Huang P, Lin L, Liu X, et al. Genomic and Epigenomic Heterogeneity of Hepatocellular Carcinoma. Cancer Res. 2017;77 9:2255-65. doi:10.1158/0008-5472.CAN-16-2822.
3. Gundem G, Van Loo P, Kremeyer B, Alexandrov LB, Tubio JMC, Papaemmanuil E, et al. The evolutionary history of lethal metastatic prostate cancer. Nature. 2015;520 7547:353-7. doi:10.1038/nature14347.
4. Hong MK, Macintyre G, Wedge DC, Van Loo P, Patel K, Lunke S, et al. Tracking the origins and drivers of subclonal metastatic expansion in prostate cancer. Nat Commun. 2015;6:6605. doi:10.1038/ncomms7605.
5. Hao JJ, Lin DC, Dinh HQ, Mayakonda A, Jiang YY, Chang C, et al. Spatial intratumoral heterogeneity and temporal clonal evolution in esophageal squamous cell carcinoma. 1546-1718 (Electronic).
6. Yates LR, Gerstung M, Knappskog S, Desmedt C, Gundem G, Van Loo P, et al. Subclonal diversification of primary breast cancer revealed by multiregion sequencing. Nat Med. 2015;21 7:751-9. doi:10.1038/nm.3886.
7. de Bruin EC, McGranahan N, Mitter R, Salm M, Wedge DC, Yates L, et al. Spatial and temporal diversity in genomic instability processes defines lung cancer evolution. Science. 2014;346 6206:251-6. doi:10.1126/science.1253462.
8. Zhang J, Fujimoto J, Zhang J, Wedge DC, Song X, Zhang J, et al. Intratumor heterogeneity in localized lung adenocarcinomas delineated by multiregion sequencing. Science. 2014;346 6206:256-9. doi:10.1126/science.1256930.
9. Patel AP, Tirosh I, Trombetta JJ, Shalek AK, Gillespie SM, Wakimoto H, et al. Single-cell RNA-seq highlights intratumoral heterogeneity in primary glioblastoma. Science. 2014;344 6190:1396-401. doi:10.1126/science.1254257.
10. Jamal-Hanjani M, Wilson GA, McGranahan N, Birkbak NJ, Watkins TBK, Veeriah S, et al. Tracking the Evolution of Non–Small-Cell Lung Cancer. New Engl J Med. 2017;376 22:2109-21. doi:10.1056/NEJMoa1616288.
11. Gerlinger M, Rowan AJ, Horswell S, Math M, Larkin J, Endesfelder D, et al. Intratumor heterogeneity and branched evolution revealed by multiregion sequencing. N Engl J Med. 2012;366 10:883-92. doi:10.1056/NEJMoa1113205.

12. Hu Z, Ding J, Ma Z, Sun R, Seoane JA, Scott Shaffer J, et al. Quantitative evidence for early metastatic seeding in colorectal cancer. Nat Genet. 2019;51 7:1113-22. doi:10.1038/s41588-019-0423-x.
13. Kim TM, Jung SH, An CH, Lee SH, Baek IP, Kim MS, et al. Subclonal Genomic Architectures of Primary and Metastatic Colorectal Cancer Based on Intratumoral Genetic Heterogeneity. Clin Cancer Res. 2015;21 19:4461-72. doi:10.1158/1078-0432.CCR-14-2413.
14. El-Kebir M, Oesper L, Acheson-Field H and Raphael BJ. Reconstruction of clonal trees and tumor composition from multi-sample sequencing data. Bioinformatics. 2015;31 12:i62-70. doi:10.1093/bioinformatics/btv261.
15. Gerlinger M, Horswell S, Larkin J, Rowan AJ, Salm MP, Varela I, et al. Genomic architecture and evolution of clear cell renal cell carcinomas defined by multiregion sequencing. Nat Genet. 2014;46 3:225-33. doi:10.1038/ng.2891.
16. Liu W, Laitinen S, Khan S, Vihinen M, Kowalski J, Yu G, et al. Copy number analysis indicates monoclonal origin of lethal metastatic prostate cancer. Nat Med. 2009;15 5:559-65. doi:10.1038/nm.1944.
17. Huang Y, Gao S, Wu S, Song P, Sun X, Hu X, et al. Multilayered molecular profiling supported the monoclonal origin of metastatic renal cell carcinoma. Int J Cancer. 2014;135 1:78-87. doi:10.1002/ijc.28654.
18. Cheung KJ, Padmanaban V, Silvestri V, Schipper K, Cohen JD, Fairchild AN, et al. Polyclonal breast cancer metastases arise from collective dissemination of keratin 14-expressing tumor cell clusters. Proc Natl Acad Sci U S A. 2016;113 7:E854-63. doi:10.1073/pnas.1508541113.
19. Beltran H and Demichelis F. Prostate cancer: Intrapatient heterogeneity in prostate cancer. Nat Rev Urol. 2015;12 8:430-1. doi:10.1038/nrurol.2015.182.
20. Wang D, Niu X, Wang Z, Song CL, Huang Z, Chen KN, et al. Multiregion Sequencing Reveals the Genetic Heterogeneity and Evolutionary History of Osteosarcoma and Matched Pulmonary Metastases. Cancer Res. 2019;79 1:7-20. doi:10.1158/0008-5472.CAN-18-1086.
21. Yan T, Cui H, Zhou Y, Yang B, Kong P, Zhang Y, et al. Multi-region sequencing unveils novel actionable targets and spatial heterogeneity in esophageal squamous cell carcinoma. Nat Commun. 2019;10 1:1670. doi:10.1038/s41467-019-09255-1.
22. Mayakonda A, Lin D-C, Assenov Y, Plass C and Koeffler HP. Maftools: efficient and comprehensive analysis of somatic variants in cancer. Genome research. 2018;28 11:1747-56. doi:10.1101/gr.239244.118.
23. Blokzijl F, Janssen R, van Boxtel R and Cuppen E. MutationalPatterns: comprehensive genome-wide analysis of mutational processes. Genome Med. 2018;10 1:33. doi:10.1186/s13073-018-0539-0.
24. Rosenthal R, McGranahan N, Herrero J, Taylor BS and Swanton C. DeconstructSigs: delineating mutational processes in single tumors distinguishes DNA repair deficiencies and patterns of carcinoma evolution. Genome Biol. 2016;17:31. doi:10.1186/s13059-016-0893-4.

25. Popic V, Salari R, Hajirasouliha I, Kashef-Haghighi D, West RB and Batzoglou S. Fast and scalable inference of multi-sample cancer lineages. Genome Biol. 2015;16:91. doi:10.1186/s13059-015-0647-8.
26. Niknafs N, Beleva-Guthrie V, Naiman DQ and Karchin R. SubClonal Hierarchy Inference from Somatic Mutations: Automatic Reconstruction of Cancer Evolutionary Trees from Multi-region Next Generation Sequencing. PLoS Comput Biol. 2015;11 10:e1004416. doi:10.1371/journal.pcbi.1004416.
27. Cibulskis K, Lawrence MS, Carter SL, Sivachenko A, Jaffe D, Sougnez C, et al. Sensitive detection of somatic point mutations in impure and heterogeneous cancer samples. Nat Biotechnol. 2013;31 3:213-9. doi:10.1038/nbt.2514.
28. Stachler MD, Taylor-Weiner A, Peng S, McKenna A, Agoston AT, Odze RD, et al. Paired exome analysis of Barrett's esophagus and adenocarcinoma. Nat Genet. 2015;47 9:1047-55. doi:10.1038/ng.3343.
29. Li H, Handsaker B, Wysoker A, Fennell T, Ruan J, Homer N, et al. The Sequence Alignment/Map format and SAMtools. Bioinformatics. 2009;25 16:2078-9. doi:10.1093/bioinformatics/btp352.
30. Roth A, Khattra J, Yap D, Wan A, Laks E, Biele J, et al. PyClone: statistical inference of clonal population structure in cancer. Nat Methods. 2014;11 4:396-8. doi:10.1038/nmeth.2883.
31. Boeva V, Popova T, Bleakley K, Chiche P, Cappo J, Schleiermacher G, et al. Control-FREEC: a tool for assessing copy number and allelic content using next-generation sequencing data. Bioinformatics. 2012;28 3:423-5. doi:10.1093/bioinformatics/btr670.
32. Shen R and Seshan VE. FACETS: allele-specific copy number and clonal heterogeneity analysis tool for high-throughput DNA sequencing. Nucleic Acids Res. 2016;44 16:e131. doi:10.1093/nar/gkw520.
33. Zhang C, Zhang L, Xu T, Xue R, Yu L, Zhu Y, et al. Mapping the spreading routes of lymphatic metastases in human colorectal cancer. Nat Commun. 2020;11 1:1993. doi:10.1038/s41467-020-15886-6.
34. Hu Z, Li Z, Ma Z and Curtis C. Multi-cancer analysis of clonality and the timing of systemic spread in paired primary tumors and metastases. Nat Genet. 2020; doi:10.1038/s41588-020-0628-z.
35. McGranahan N, Favero F, de Bruin EC, Birkbak NJ, Szallasi Z and Swanton C. Clonal status of actionable driver events and the timing of mutational processes in cancer evolution. Sci Transl Med. 2015;7 283:283ra54. doi:10.1126/scitranslmed.aaa1408.
36. Ullah I, Karthik GM, Alkodsi A, Kjallquist U, Stalhammar G, Lovrot J, et al. Evolutionary history of metastatic breast cancer reveals minimal seeding from axillary lymph nodes. J Clin Invest. 2018;128 4:1355-70. doi:10.1172/JCI96149.
37. Sun R, Hu Z, Sottoriva A, Graham TA, Harpak A, Ma Z, et al. Between-region genetic divergence reflects the mode and tempo of tumor evolution. Nat Genet. 2017;49 7:1015-24. doi:10.1038/ng.3891.

38. Bhatia G, Patterson N, Sankararaman S and Price AL. Estimating and interpreting FST: the impact of rare variants. Genome Res. 2013;23 9:1514-21. doi:10.1101/gr.154831.113.
39. Lee JK, Wang J, Sa JK, Ladewig E, Lee HO, Lee IH, et al. Spatiotemporal genomic architecture informs precision oncology in glioblastoma. Nat Genet. 2017;49 4:594-9. doi:10.1038/ng.3806.
40. Mroz EA, Tward AD, Pickering CR, Myers JN, Ferris RL and Rocco JW. High intratumor genetic heterogeneity is related to worse outcome in patients with head and neck squamous cell carcinoma. Cancer-Am Cancer Soc. 2013;119 16:3034-42. doi:10.1002/cncr.28150.
41. Charoentong P, Finotello F, Angelova M, Mayer C, Efremova M, Rieder D, et al. Pan-cancer Immunogenomic Analyses Reveal Genotype-Immunophenotype Relationships and Predictors of Response to Checkpoint Blockade. Cell reports. 2017;18 1:248-62. doi:10.1016/j.celrep.2016.12.019.
42. Ding L, Ley TJ, Larson DE, Miller CA, Koboldt DC, Welch JS, et al. Clonal evolution in relapsed acute myeloid leukaemia revealed by whole-genome sequencing. Nature. 2012;481 7382:506-10. doi:10.1038/nature10738.
43. Griffith M, Miller CA, Griffith OL, Krysiak K, Skidmore ZL, Ramu A, et al. Optimizing cancer genome sequencing and analysis. Cell Syst. 2015;1 3:210-23. doi:10.1016/j.cels.2015.08.015.
44. Xue R, Chen L, Zhang C, Fujita M, Li R, Yan SM, et al. Genomic and Transcriptomic Profiling of Combined Hepatocellular and Intrahepatic Cholangiocarcinoma Reveals Distinct Molecular Subtypes. 1878-3686 (Electronic).
45. Paradis E, Claude J and Strimmer K. APE: Analyses of Phylogenetics and Evolution in R language. Bioinformatics. 2004;20 2:289-90. doi:10.1093/bioinformatics/btg412.
46. Schliep KP. phangorn: phylogenetic analysis in R. Bioinformatics. 2011;27 4:592-3. doi:10.1093/bioinformatics/btq706.
47. Saitou N and Nei M. The neighbor-joining method: a new method for reconstructing phylogenetic trees. Mol Biol Evol. 1987;4 4:406-25. doi:10.1093/oxfordjournals.molbev.a040454.
48. Desper R and Gascuel O. Fast and accurate phylogeny reconstruction algorithms based on the minimum-evolution principle. J Comput Biol. 2002;9 5:687-705. doi:10.1089/106652702761034136.
49. Yang Z. Phylogenetic analysis using parsimony and likelihood methods. J Mol Evol. 1996;42 2:294-307.
50. Felsenstein J. Evolutionary trees from DNA sequences: a maximum likelihood approach. J Mol Evol. 1981;17 6:368-76. doi:10.1007/bf01734359.
51. Youssoufian H, Kazazian HH, Jr., Phillips DG, Aronis S, Tsiftis G, Brown VA, et al. Recurrent mutations in haemophilia A give evidence for CpG mutation hotspots. Nature. 1986;324 6095:380-2. doi:10.1038/324380a0.

52. Hu Z, Sun R and Curtis C. A population genetics perspective on the determinants of intra-tumor heterogeneity. Biochim Biophys Acta Rev Cancer. 2017;1867 2:109-26. doi:10.1016/j.bbcan.2017.03.001.
53. Liu M, Liu Y, Di J, Su Z, Yang H, Jiang B, et al. Multi-region and single-cell sequencing reveal variable genomic heterogeneity in rectal cancer. BMC Cancer. 2017;17 1:787. doi:10.1186/s12885-017-3777-4.
54. Brannon AR, Vakiani E, Sylvester BE, Scott SN, McDermott G, Shah RH, et al. Comparative sequencing analysis reveals high genomic concordance between matched primary and metastatic colorectal cancer lesions. Genome Biol. 2014;15 8:454. doi:10.1186/s13059-014-0454-7.
55. Tan IB, Malik S, Ramnarayanan K, McPherson JR, Ho DL, Suzuki Y, et al. High-depth sequencing of over 750 genes supports linear progression of primary tumors and metastases in most patients with liver-limited metastatic colorectal cancer. Genome Biol. 2015;16:32. doi:10.1186/s13059-015-0589-1.
56. Wang K, Lim HY, Shi S, Lee J, Deng S, Xie T, et al. Genomic landscape of copy number aberrations enables the identification of oncogenic drivers in hepatocellular carcinoma. Hepatology. 2013;58 2:706-17. doi:10.1002/hep.26402.
57. Guichard C, Amaddeo G, Imbeaud S, Ladeiro Y, Pelletier L, Maad IB, et al. Integrated analysis of somatic mutations and focal copy-number changes identifies key genes and pathways in hepatocellular carcinoma. Nat Genet. 2012;44 6:694-8. doi:10.1038/ng.2256.
58. Dagogo-Jack I and Shaw AT. Tumour heterogeneity and resistance to cancer therapies. Nat Rev Clin Oncol. 2018;15 2:81-94. doi:10.1038/nrclinonc.2017.166.
59. Scrucca L, Fop M, Murphy TB and Raftery AE. mclust 5: Clustering, Classification and Density Estimation Using Gaussian Finite Mixture Models. R J. 2016;8 1:289-317.
60. Rajput A, Bocklage T, Greenbaum A, Lee JH and Ness SA. Mutant-Allele Tumor Heterogeneity Scores Correlate With Risk of Metastases in Colon Cancer. Clin Colorectal Cancer. 2017;16 3:e165-e70. doi:10.1016/j.clcc.2016.11.004.
61. Mroz EA and Rocco JW. MATH, a novel measure of intratumor genetic heterogeneity, is high in poor-outcome classes of head and neck squamous cell carcinoma. Oral Oncol. 2013;49 3:211-5. doi:10.1016/j.oraloncology.2012.09.007.
62. Campbell PJ, Yachida S, Mudie LJ, Stephens PJ, Pleasance ED, Stebbings LA, et al. The patterns and dynamics of genomic instability in metastatic pancreatic cancer. Nature. 2010;467 7319:1109-13. doi:10.1038/nature09460.
63. Alexandrov LB, Nik-Zainal S, Wedge DC, Aparicio SA, Behjati S, Biankin AV, et al. Signatures of mutational processes in human cancer. Nature. 2013;500 7463:415-21. doi:10.1038/nature12477.

64. Mengni Liu (2021) Supporting code and data for "MesKit: a tool kit for dissecting cancer evolution of multi-region tumor biopsies through somatic alterations" [Source Code]. https://doi.org/10.24433/CO.6811520.v1

Figure 1

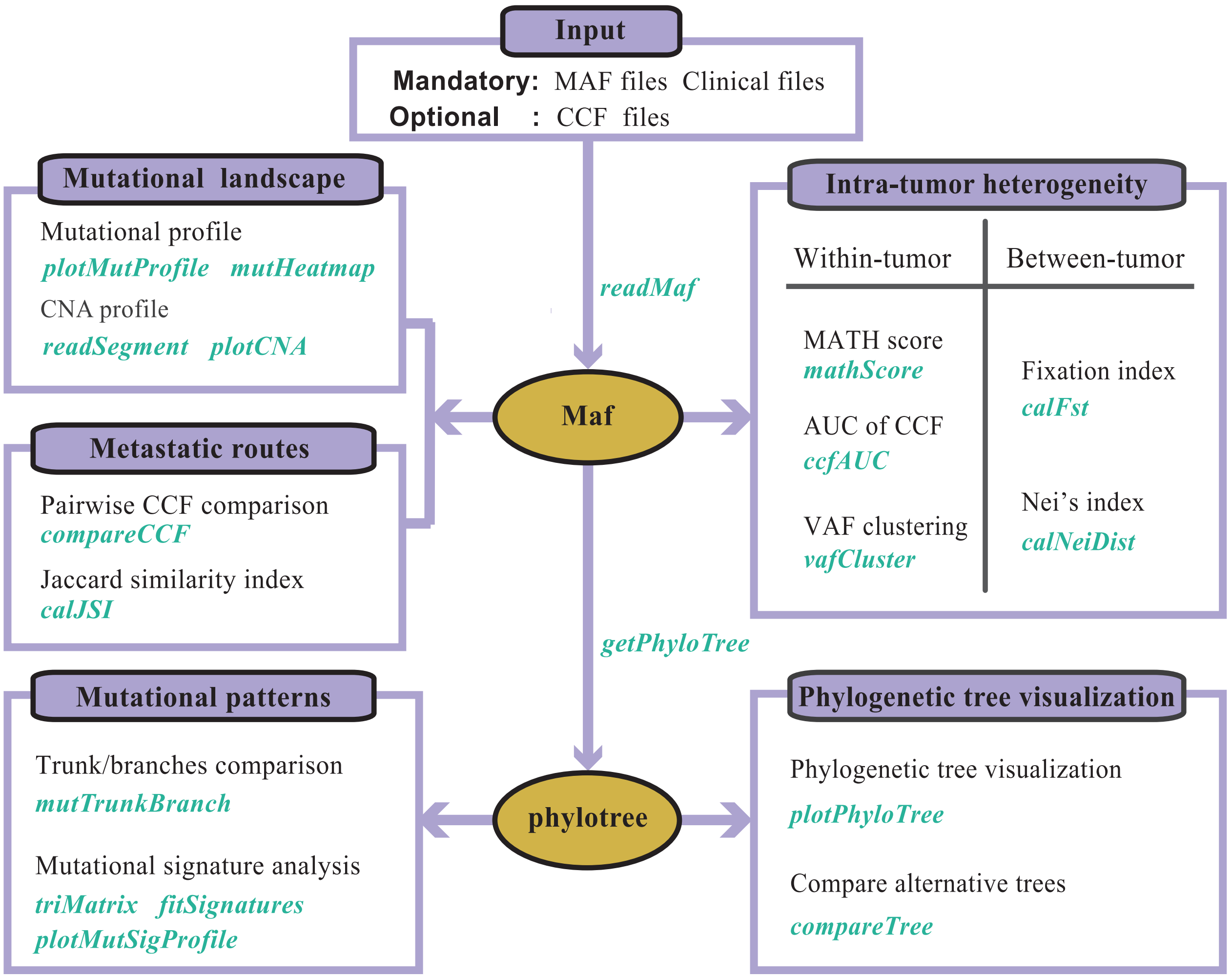
Input
Mandatory: MAF files Clinical files
Optional : CCF files
readMaf
Maf
getPhyloTree
phylotree
Mutational landscape
Mutational profile
plotMutProfile mutHeatmap
CNA profile
readSegment plotCNA
Metastatic routes
Pairwise CCF comparison
compareCCF
Jaccard similarity index
calJSI
Intra-tumor heterogeneity
Within-tumor
Between-tumor
MATH score
mathScore
AUC of CCF
ccfAUC
VAF clustering
vafCluster
Fixation index
calFst
Nei's index
calNeiDist
Mutational patterns
Trunk/branches comparison
mutTrunkBranch
Mutational signature analysis
triMatrix fitSignatures
plotMutSigProfile
Phylogenetic tree visualization
Phylogenetic tree visualization
plotPhyloTree
Compare alternative trees
compareTree

Figure 2

Click here to access/download;Figure;Figure2.pdf

Figure 2

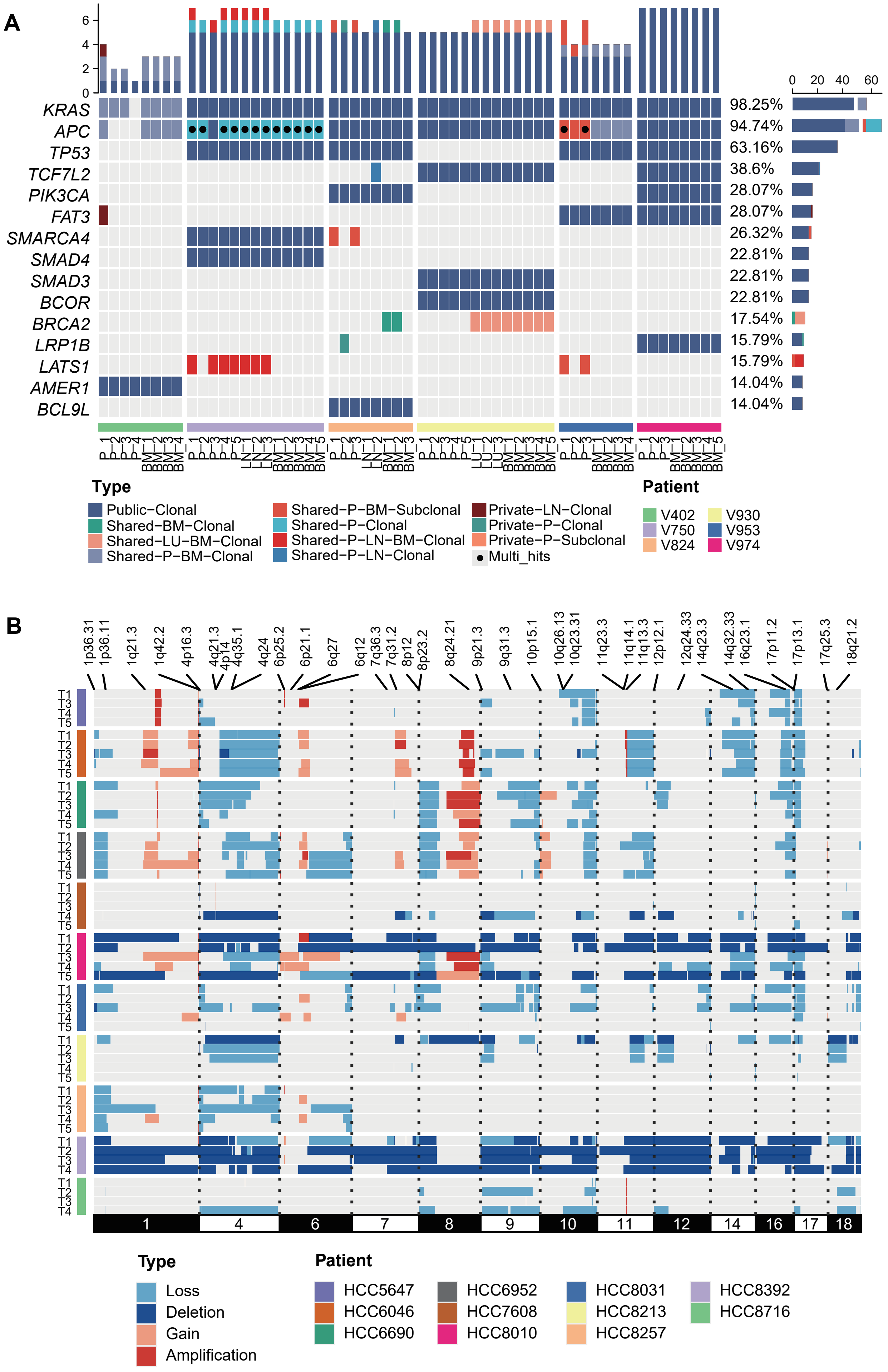

Figure 3

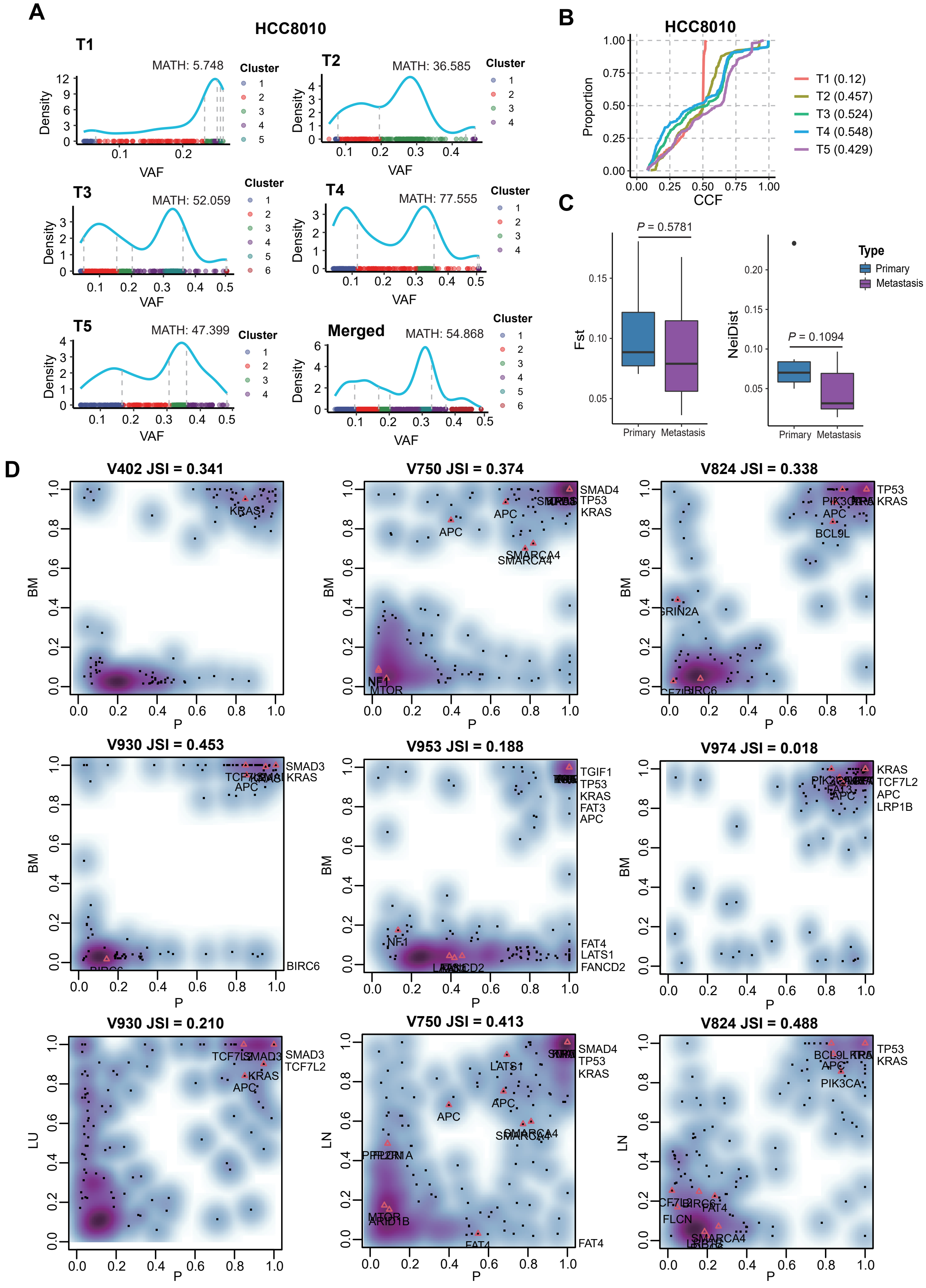
A
HCC8010
T1
MATH: 5.748
T2
MATH: 36.585
T3
MATH: 52.059
T4
MATH: 77.555
T5
MATH: 47.399
Merged
MATH: 54.868
Cluster
Density
VAF
B
HCC8010
Proportion
CCF
T1 (0.12)
T2 (0.457)
T3 (0.524)
T4 (0.548)
T5 (0.429)
C
P = 0.5781
Fst
Primary Metastasis
NeiDist
P = 0.1094
Type
Primary
Metastasis
D
V402 JSI = 0.341
V750 JSI = 0.374
V824 JSI = 0.338
V930 JSI = 0.453
V953 JSI = 0.188
V974 JSI = 0.018
V930 JSI = 0.210
V750 JSI = 0.413
V824 JSI = 0.488
BM
LU
LN
P

Figure 4 Click here to access/download;Figure;Figure4.pdf

Figure 4

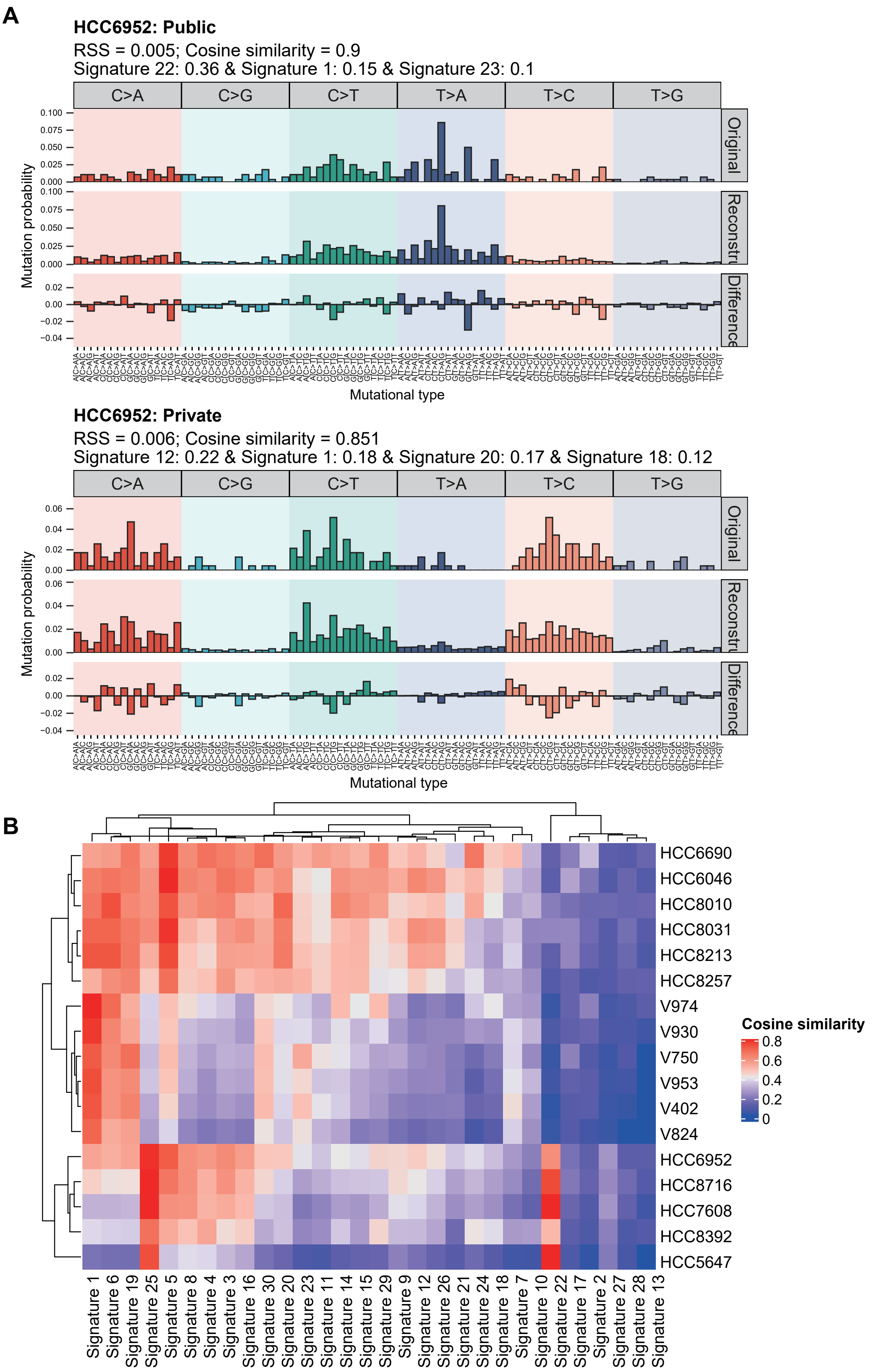

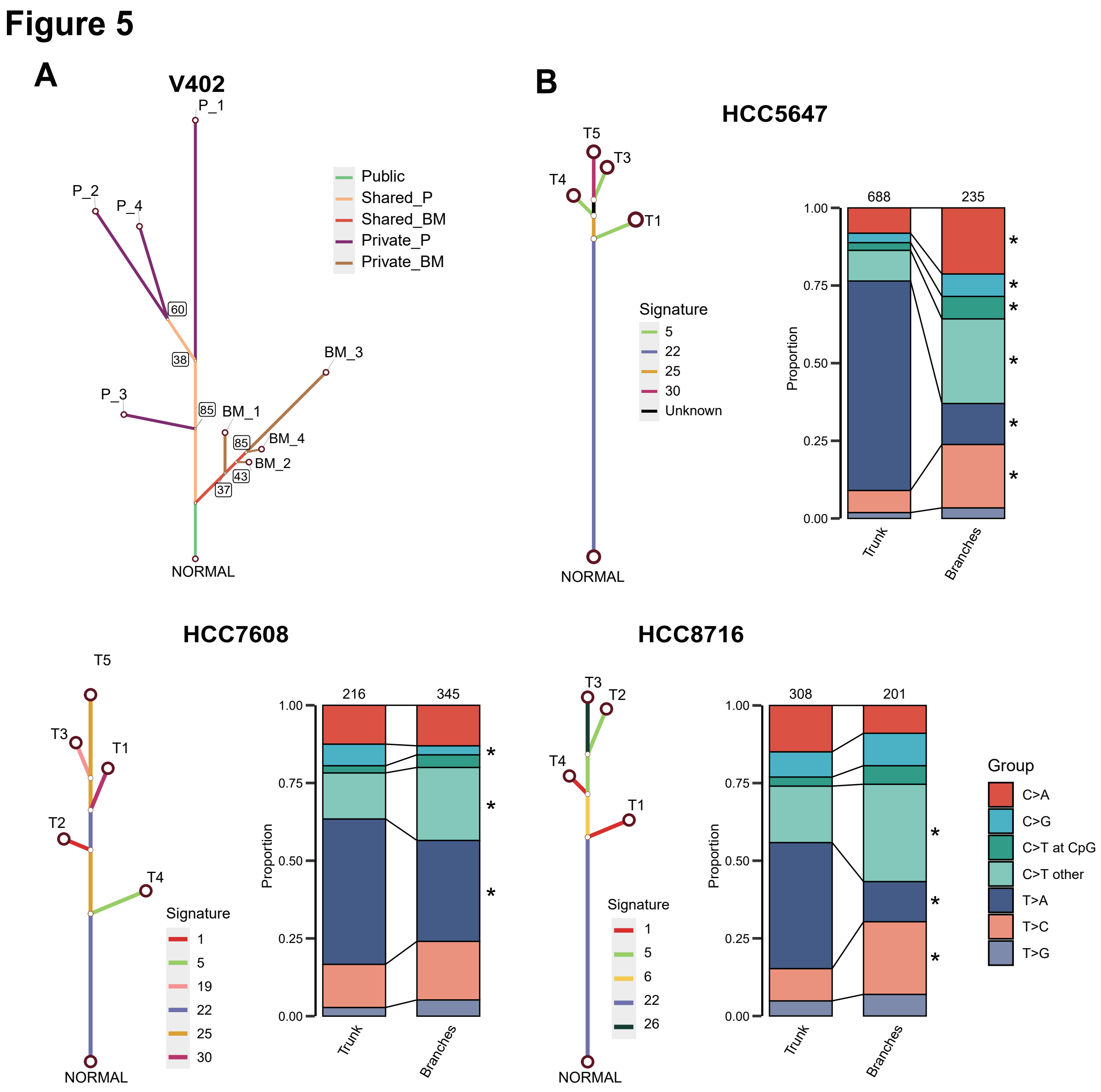
Figure 5
A
V402
P_1
P_2
P_4
P_3
BM_1
BM_2
BM_3
BM_4
NORMAL
60
38
85
85
43
37
Public
Shared_P
Shared_BM
Private_P
Private_BM
B
HCC5647
T5
T3
T4
T1
NORMAL
Signature
5
22
25
30
Unknown
688
235
Proportion
1.00
0.75
0.50
0.25
0.00
Trunk
Branches
HCC7608
T5
T3
T1
T2
T4
NORMAL
Signature
1
5
19
22
25
30
216
345
HCC8716
T3
T2
T4
T1
NORMAL
Signature
1
5
6
22
26
308
201
Group
C>A
C>G
C>T at CpG
C>T other
T>A
T>C
T>G

Supplementary Figure S1

Click here to access/download
Supplementary Material
Supplementary Figure S1.pdf

Supplementary Figure S2

Click here to access/download
Supplementary Material
Supplementary Figure S2.pdf

Supplementary Figure S3

Click here to access/download
Supplementary Material
Supplementary Figure S3.pdf

Supplementary Figure S4

Click here to access/download
Supplementary Material
Supplementary Figure S4.pdf

Click here to access/download
Supplementary Material
Supplementary File S1.docx

Supplementary Table S1

Click here to access/download
Supplementary Material
Supplementary Table S1.docx

Supplementary Table S2

Click here to access/download
Supplementary Material
Supplementary Table S2.xlsx
