# Supplementary material for: MesKit: a tool kit for dissecting cancer evolution of multi-region tumor biopsies through somatic alterations
Source: Gigascience. 2021 May 21;10(5):giab036. doi: 10.1093/gigascience/giab036 (PMC8138830; doi:10.1093/gigascience/giab036)
Supplement: giab036_GIGA-D-21-00007_Revision_1 [file giab036_giga-d-21-00007_revision_1.pdf]

GigaScience

MesKit: a tool kit for dissecting cancer evolution of multi-region tumor biopsies through somatic alterations

--Manuscript Draft--

Manuscript Number:	GIGA-D-21-00007R1
Full Title:	MesKit: a tool kit for dissecting cancer evolution of multi-region tumor biopsies through somatic alterations
Article Type:	Technical Note
Funding Information:	National Key R&D Program of China (2017YFA0106700) — Dr Jian Ren<br>Fundamental Research Funds for the Central Universities (SYSU: 19ykpy184) — Dr Qi Zhao
Abstract:	Background<br>Multi-region sequencing (MRS) has been widely used to analyze intra-tumor heterogeneity (ITH) and cancer evolution. However, comprehensive analysis of mutational data from MRS is still challenging, which requires complicated integration of a plethora of computational and statistical approaches.<br>Findings<br>Here, we present MesKit, an R/Bioconductor package, that can assist in characterizing genetic ITH and tracing the evolutionary history of tumors based on somatic alterations detected by MRS. MesKit provides a wide range of analysis and visualization modules, including ITH evaluation, metastatic route inference, and mutational signature identification. In addition, MesKit implements an auto-layout algorithm to generate phylogenetic trees based on somatic mutations. The application of MesKit for two reported MRS datasets of hepatocellular carcinoma (HCC) and colorectal cancer (CRC) identified known heterogeneous features and evolutionary patterns, together with potential driver events during cancer evolution.<br>Conclusions<br>In summary, MesKit is useful for interpreting ITH and tracing evolutionary trajectory based on MRS data. MesKit is implemented in R and available at https://bioconductor.org/packages/MesKit under the GPL v3 license.
Corresponding Author:	Qi Zhao<br>Sun Yat-Sen University Cancer Center<br>Guangzhou, Guangdong CHINA
Corresponding Author Secondary Information:	
Corresponding Author's Institution:	Sun Yat-Sen University Cancer Center
Corresponding Author's Secondary Institution:	
First Author:	Mengni Liu
First Author Secondary Information:	
Order of Authors:	Mengni Liu<br>Jianyu Chen<br>Xin Wang<br>Chengwei Wang<br>Xiaolong Zhang<br>Yubin Xie<br>Zhixiang Zuo<br>Jian Ren

Powered by Editorial Manager® and ProduXion Manager® from Aries Systems Corporation

	Qi Zhao
**Order of Authors Secondary Information:**	
**Response to Reviewers:**	Dear editor and reviewers<br><br>We are very grateful to the reviewers for their careful reading of the manuscript and constructive remarks. We have addressed all the issues raised by the reviewers and paid heed to their advices and suggestions. The manuscript has been massively revised. Please find below a point-by-point reply to the reviewers' comments. According to your suggestion, we included RRID (SCR_020959) and bio.tools ID (meskit) in the manuscript. In addition, we have sent our revised manuscript to professional English-editing service provider (AJE) for improvement of the manuscript. We believe that the contents and the clarity of our paper are much improved in the revised version.<br><br><br>---<br>Point by point response to revisers<br><br>Reviewer #1:<br>In their paper 'MesKit: a tool kit for dissecting cancer evolution of multi-region tumor biopsies through somatic alterations', the authors describe a new R package for analysis of multi-region tumour sequencing data (MRS) which comprises many of the commonly employed workflows and statistics into a convenient toolset.<br>I believe this is a useful and interesting piece of software, which might find broad application in the field. I particularly like the assembly of different heterogeneity measures within and between tumours. The tool is well documented and the online vignette is detailed and well made. I have only a few points that I would like to see addressed.<br><br>[Response]: Thank you very much for the positive and constructive comments. We have duly followed your comments and suggestions to address all concerns as elaborated in the following responses. In particular, we would like to thank you for your constructive comments which have improved the manuscript in numerous ways. Thank you very much for your precious time and kind attention.<br><br>Major points:<br>=============<br><br>1.) The authors position their tool as a general analysis toolkit for MRS datasets. However, the tool seems very much focused on somatic point mutations and provides little in terms of somatic-copy-number alteration (SCNA) analysis. SCNAs seem to be mainly used to correctly estimate CCFs. This is fine, but it needs to be stated clearly. I recommend extending the introduction to include and distinguish between heterogeneity measures and phylogeny reconstructions on SCNAs and those on SNVs. This way, the authors can give the reader a better understanding what MesKit does and does not provide.<br><br>[Response]: Thank you for this helpful comment. To describe the characteristics of MesKit more clearly, we modified the Introduction as follow: "MesKit was designed as an easy-to-use R package that only requires a Mutation Annotation Format (MAF) file and a clinical file as inputs, enabling researchers to evaluate the contribution of point mutations to heterogeneity within/between tumors from the same patient. MesKit can also be used to depict mutational profiles, track evolutionary dynamics and characterize mutational patterns at different<br>levels.".<br><br>2.) The discussion should also be adjusted accordingly. Please be more critical and make clear what the current drawbacks of MesKit are and where it might not be applicable. Again, bring up the point from the introduction, that MesKit is SNV-centric and that support for SCNA analysis so far is limited.<br><br>[Response]: Thank you for this valuable comment. According to your suggestion, we have revised the discussion as follow: "As MesKit takes a MAF file and a clinical data

Powered by Editorial Manager® and ProduXion Manager® from Aries Systems Corporation

file as standard inputs, it primarily evaluates ITH based on somatic mutations, and its assessment of contributions of CNAs is still limited. Currently, several subclonal reconstruction methods are available to infer the relative order of occurrence between a SNV and its associated CNA. In future updates, we plan to implement the integration of results from these methods to provide insight into the clonality and temporal dynamics of ITH…"

3.) The criterion for calling a SNV subclonal based on the merged CCF is unclear to me. There might be more information in the refs provided, but the paper needs to stand alone, so the authors need to at least provide a general idea of the motivation behind a 'merged CCF' and the specific cutoff of 0.5.

[Response]: We apologize for the confusion. Accordingly, we made a more detailed explanation about the "merged CCF" in the revised manuscript as follows (Methods-Clonal status of somatic mutations): "Since multiple samples collected from a single tumor collectively reflect its clonal composition, these regions should be considered as a whole to capture the overall tumor dynamics. Here, we assume that each tumor follows neutral exponential growth in a well-mixed population [39, 40]. When MRS data are available, the merged CCF (CCF_merged) of each mutation is computed by integrating multiple regions as previously described [12, 41, 42]…". And the CCF cutoff of "0.5" was chosen for its good performance in defining subclonality based on simulated virtual tumors, as demonstrated by Sun et al. Nature Genetics, 2017 [1].

Minor points:
=============

* Introduction, paragraph 1: "quantitative interpretation of heterogeneity..."I don't understand that sentence.

[Response]: Sorry for the confused phrase. We have corrected it accordingly.

* page 6: "visualization for MRS dataset yet" -> "datasets" (plural)

[Response]: Thank you very much for pointing this out. We have corrected it accordingly.

* Material and Methods (page 7): "Each sample was sequenced [...] to high coverage". It is very subjective what comprises high coverage. For example TRACERx sequencing coverage is > 400x. Please remove 'high'.

[Response]: Thank you very much for pointing this out. We have corrected it accordingly.

* Material and Methods (page 7): 'CRC cohort consisted of [...]' -> 'The CRC cohort...'

[Response]: Thank you very much for pointing this out. We have corrected it accordingly.

* Estimation of ITH: 'Fst' should be capitalised throughout (FST)

[Response]: Thank you very much for pointing this out. We have corrected it accordingly.

* Inference of metastatic routes: 'which identifies subclones shared between different regions via CCF plots': I was expecting a Figure reference here. Please provide one that shows a CCF plot and what it does.

[Response]: Thanks for your valuable comments. According to your suggestion, we have added a reference of CCF plot and revised manuscript as follow (Results-Inferring the clonality of metastatic seeding): "Given that mutations with similar CCFs tend to cluster into the same subpopulation [69, 70], many studies inferred the potential metastatic routes between different lesions from the same patient by plotting CCFs of mutations [12, 42, 71]. By this means Xue et al. [71] identified both monoclonal and multiclonal origins of separate type combined hepatocellular and intrahepatic

cholangiocarcinoma (cHCC-ICC).”

Reviewer #2:
In this manuscript, Liu et al present MesKit, an R package designed to study tumor heterogeneity through multiple samples sequencing, with examples coming from published data in hepatocellular carcinoma and colorectal cancer. The package is already available in R/Bioconductor, with an example script including embedded data, allowing anyone to test the functionalities. MesKit represents a useful tool since it wraps together multiple methods already described in various publications, and allows the usage of simple inputs (maf files) to study tumor heterogeneity. Moreover, a shiny application has been developed by the authors, allowing usage of the tool by researchers lacking bioinformatics skills.

[Response]: Thank you very much for the constructive and positive comments. We have duly followed your comments and suggestions to address all concerns elaborately in the following responses.

Here are my comments:

Q1. The formula used to calculate the AUC of CCF is not correct, therefore the values obtained are underestimated: in the ccfAUC function, the integration is done between min(df_ccf$CCF) and max(df_ccf$CCF) but it should be done between 0 and 1. For instance, the AUC of HCC8010_T1 (Fig3B) is missing all the area between 0.50 and 1, and therefore it should be the highest of the patient instead of being the smallest.

[Response]: Thanks for pointing out this mistake, we have corrected it accordingly.

Q2. For the vafCluster function (Fig3A), it is not really clear if the function takes into account the multiple regions and thus does a clustering in multiple dimensions, or just does independent Gaussian mixture in one dimension for each region. Is there a relation between the clusters (1-6) defined in each region? In any case, I'm not sure that the Gaussian finite mixture model is a good way to separate clusters of mutations corresponding to subclones. The usual approach since the seminal paper of Nik-Zainal et al, Cell 2012 is to use a Dirichlet process.

[Response]: We apologize for the confusion. The clusters of different regions are uncorrelated. The vafCluster function performs clustering in one dimension for each region/tumor based on the independent Gaussian mixture model. This model has been employed in multiple studies for 1D clustering [2, 3]. Furthermore, considering the clustering of observed VAFs without consideration of copy number may not be informative, we have modified the clustering strategy implemented in the function vafCluster and renamed it to mutCluster for accuracy and relevance. Details of this analysis were revised in the manuscript as follow (Methods-ITH estimation): “For a single region/tumor, it is common to infer subpopulations of tumor cells by clustering VAFs or CCFs [26, 30]. To implement this process, we employed Gaussian finite mixture models for one-dimensional clustering of VAFs or CCFs using mclust R package [46]. As copy number gains and losses can alter the fraction of reads bearing a mutation, we only focused on heterozygous mutations within copy-number neutral and loss of heterozygosity (LOH)-free regions when clustering VAFs.”. We have also made this clear in the vignette. Additionally, a prompting message will be printed when calling the function mutCluster as “## Performing one-dimensional clustering for …”. Although many existing methods rely on some form of “Dirichlet process” clustering, such as sciClone [4], DPClust [5], PyClone [6] and PhyloWGS [7], there is still a lack of thorough assessment of them. We will continue to pay attention to this fast-evolving field and consider the integration of clustering results of other tools in future updates. Thanks again for your valuable comments!

Q3. Relatedly, the 2-dimension representation of CCF (Fig3D) would clearly benefit of an estimation of clusters corresponding to subclones, either through a Dirichlet process, as performed in Gundem et al 2015, or just a clustering of the mutations in multiple dimensions. It would facilitate the interpretation of the plots and the evaluation of the monoclonal or polyclonal seeding of metastasis.

[Response]: Thanks for your comments. Yes, Gundern et al performed two-

dimensional clustering between metastatic sites, but our intension was to present comparison between lesions (primary tumor/metastasis pair) with multiple sites sequenced. To our knowledge, no appropriate method has been specifically developed for comparison of paired lesions with MRS data. To address your concerns, we employed SciClone [4] to cluster merged VAFs (equivalent to CCF_merged/2, see Methods for details) of each primary/metastasis tumor pair. As shown in Figure R1 (https://github.com/Niinleslie/MesKit/blob/paper/inst/plot/Figure_R1.md), mutations were indicated in different shapes according to the cluster they correspond to, which was consistent with the density-based distribution. Mutations with similar CCFs density were likely to cluster together by SciClone [4]. Still, we will continue to pay attention to this fast-evolving field and consider the integration of clustering results of other tools in future updates. Thanks again for your valuable comments!

[Figure R1] To note, as the submission system did not support figure, we have linked the figure with an external link, which could be found at
https://github.com/Niinleslie/MesKit/blob/paper/inst/plot/Figure_R1.md
Figure R1. Density plots of merged CCF values in paired primary tumors and metastases of the CRC cohort. SciClone was employed to cluster merged VAFs (equivalent to CCF_merged/2) for each primary tumor/metastasis sample pair and mutations were indicated in different shapes according to the cluster they correspond to. Jaccard similarity index (JSI) and putative CRC driver genes are indicated on the plot. P, primary tumor; BM, brain metastasis; LN, lymph node metastasis; LU, lung metastasis.

Q4. Some of the data used for examples are a little odd:

[Response]: Thanks! Your comments reminded us that there were potential artifacts in the datasets of the HCC cohort. To provide a better demonstration of MesKit, we amended the analysis pipeline and adopted more rigorous criteria for mutations calling and CNA detection. The “Method-Data collection and preprocessing” section was revised as follow: “In brief, we performed sSNV calling for each tumor/normal pair with Mutect (version 1.1.7) [33], while INDELs were detected with Strelka v2.7.1 [34]. Additionally, we adopted the “force calling” method [35] to rescue potential real mutations for each sample based on the aggregate set of somatic events in each patient using samtools mpileup (version 1.2) [36]. Both sSNVs and INDELs were annotated through ANNOVAR (v.20191024) [37]. The following filters were further applied to identify the sSNVs and INDELs: (1) Mutations with fewer than 15 total reads or 5 variant reads were discarded. (2) Mutations listed in dbSNP147 were removed unless they were documented in the Catalogue of Somatic Mutations in Cancer (COSMIC) database. (3) Mutations listed in the National Heart, Lung, and Blood Institute Exome Sequencing Project were removed. Copy number analysis based on exome sequencing was performed using Sequenza v3.0.0 [38]. Segments smaller than 500 kb were filtered and only autosomes were used in copy number analysis. CCFs of mutations were estimated by PyClone (v0.13.0) [28], which adjusted the variant allele frequencies (VAFs) of somatic mutations based on local copy numbers of the mutated loci and tumor purity (Sequenza v3.0.0) [38].”. Accordingly, we revised the corresponding results about the HCC cohort and redrew the following figures: Figure 2B, Figure 3A, B, Figure 5, Figure S1A and Figure S5.

i) in the HCC mutational landscape (Fig S1A), the frequency of KMT2C alterations is really high (69%) compared to known cohorts of HCC (< 10%), and may represent artifacts.

[Response]: Thank you very much for pointing this out. According to your suggestion, we have recalled somatic mutations as answered in Q4 and redrew the mutational landscape with updated mutation frequencies of the CRC cohort (Figure S1A). Thanks again for your comments.

ii) in the CNA data (Fig2B), there are really large deletions in dark blue, corresponding to CN=0, which is not possible for an entire chromosome, so the copy-number is probably wrong.

[Response]: Thanks for your comments. We have redrawn Figure2B based on CNAs detected by Sequenza (v3.0.0), see the response of Q4 for details. Besides, this part

Powered by Editorial Manager® and ProduXion Manager® from Aries Systems Corporation

was revised in manuscript as follow (Results-Mutational landscape from MRS studies): “Consistent with TCGA projects and other previous studies of hepatocellular carcinoma [65, 66], a number of copy number alterations were observed in our HCC cohort, such as gains of 1q, 6p, 8q and 13q as well as losses of 1p, 4q, 9q and 11q (Figure 2B).”.

iii) in the CCF distribution of HCC8010 samples, region T1 has only mutations with CCF < 0.5, i.e not a single clonal mutation. This is likely an artifact due to a wrong estimation of the tumor purity used to obtain CCF from VAF data.

[Response]: Thanks for your comments. We have redrawn the CCF distribution of HCC8010 (Figure 3B).

-> it would be better to have cleaner data for the examples, however this doesn't represent a problem of the tool itself, just a problem of the data used as input.

[Response]: We thank the reviewer for the valuable suggestion. We have re-analyzed the datasets of the HCC cohort as mentioned above. Please refer to the above response of Q4 for details.

Q5. It is really useful to be able to run examples from the package, however the data obtained sometimes differ from the one represented in the figures: for instance, the MATH values for HCC8010 samples are really different in the R example and Fig3A (T1 is 83.3 instead of 5.7), and the density plots of VAF for CHC8010 have really different profiles. Also, the AUC of CCF for HCC8010_T5 is 0.429 in Fig3B but 0.462 when running the R example script. There should be consistency between the figures plotted in the paper and the one obtained by running the script, or at least the difference should be explained.

[Response]: Thanks for your suggestion, we have uploaded all related scripts on CodeOcean. In terms of the inconsistency you mentioned here, it is likely that you used default parameters instead of those used for figures in the paper. We suggest running code in the CodeOcean for verification (https://codeocean.com/capsule/4694382/tree/v2).

Q6. For the clustering of mutational signatures (Fig4B), it might be more relevant to directly cluster the mutation matrix for the 96 trinucleotides changes, instead of adding the step of the cosine similarity with COSMIC signatures. Indeed, this extra step adds a level of approximation, and some mutations might account for multiple signatures (for instance the numerous T>A mutations account for signature 22 and signature 25 in HCC patients exposed to aristolochic acid).

[Response]: Thanks for your comments. Yes, clustering the mutation matrix is a more direct way to compare the mutational profiles between samples. However, we presented the “clustering of mutational signatures” for its own advantages, as it can directly show the similarity in mutation profiles between samples to some degree, while at the same time providing information on which signatures are likely active. And this method has been previously used in MutationalPatterns [8].

Q7. I have no expertise in the Wright's fixation index FST, but looking at formula (10) of the article Bhatia et al, 2013, it seems that the formula (4) of the manuscript is missing a minus. Also the term mt is not defined, and the binomial coefficient (2 k) is inverted, the k should be on top. The same comment applies for Nei's distance, formulas (5) and (6).

[Response]: Thank you very much for pointing this problem out. We have corrected the formula and added the definition of “mt” in our revised manuscript as follows: “m_t represents the total number of sSNVs in region a and b”.

Minor comments:
- In Figure 1, the optional inputs should include a segmented copy-number file.

[Response]: Thanks for pointing this out. We have corrected it accordingly.

Powered by Editorial Manager® and ProduXion Manager® from Aries Systems Corporation

- In sup Table 1, data source for HCC samples is De-Chen et al, 2017 instead of Lin et al, 2017.

[Response]: We thank the reviewer for pointing out this mistake. We have corrected it accordingly.

- In the introduction, the authors could cite the Palimpsest package (Shinde et al, 2018) since it allows the identification of different mutational signatures between clonal and subclonal mutations.

[Response]: Thanks for your suggestion. We have added the citation accordingly.

- In formula (7), the value of the original reference is 1.4826 instead of 1.4268, and I don't understand why there is an "avg" subscript for VAF.

[Response]: We deeply apology for this typo error and we have corrected it accordingly.

- Signatures of HCC are in Table S2 and not File S2.

[Response]: Thanks for pointing this out. We have corrected it accordingly.

Reviewer #3:
The authors present a software tool kit called MesKit that analyses multi region sequencing studies of cancer. The tool is quite comprehensive with many different capabilities, good documentation and produces high-quality figures. Overall, I think this would be a valuable piece of software to the cancer evolution community. I do have a few concerns with regard to the interpretation of some of these analyses which I believe should be appropriately caveated.

[Response]: Thank you very much for the critical and helpful comments. We have duly followed your comments and suggestions to address all the concerns as elaborated in the following responses.

Major comments

Q1. MesKit enables clustering by VAF to interpret ITH. Without consideration of the copy number of the mutation, VAF clustering will not be informative. The authors should clarify what they are doing here. It is more appropriate to do one of the following: i) cluster using CCFs ii) cluster using some kind VAF corrected by copy number or iii) cluster VAF per copy number state (cluster on mutation only found in diploid regions for example). Another relevant point here is that there is a debate within the field around the use of clustering to identify subpopulation, see for example Caravagna et al Nature Genetics 2020. The main issue is that "clusters" at low CCF/VAF may not represent distinct populations but rather are a mixture of variants present within different lineages. Another issue is that depth of sequencing can have a large influence on the quality of clustering results (again see Caravagna et al Nature Genetics 2020). I think the challenges and possible issues with this type of analysis should be mentioned in the paper and software vignette so that users understand the caveats here.

[Response]: Thanks for your valuable suggestions. We agree that clustering of estimated VAFs is not informative without adjustment of copy number. According to your suggestion, we modified the clustering strategy implemented in the ¬¬vafCluster and renamed it to mutCluster for accuracy and relevance. The mutCluster function performs one-dimensional clustering of mutations by VAFs and CCFs. When clustering VAFs, this function only focuses on heterozygous mutations within normal diploid regions. We have made a detailed description about this analysis in the revised manuscript as follow (Methods-Estimation of ITH): “For a single region/tumor, it is common to infer subpopulations of tumor cells by clustering VAFs or CCFs [26, 30]. To implement this process, we employed Gaussian finite mixture models for one-dimensional clustering of VAFs or CCFs using mclust R package [46]. As copy number gains and losses can alter the fraction of reads bearing a mutation, we only focused on heterozygous mutations within copy-number neutral and loss of heterozygosity (LOH)-

free regions when clustering VAFs." Besides, we agree that debates and challenges of this analysis should be emphasized. We thus highlighted the debate about "low-frequency populations" and added related citations[9, 10] in the "Results-ITH estimation" section as follow: "In MesKit, the mutCluster function deduces distinct subpopulations of a sample/tumor by clustering VAFs/CCFs in one dimension based on Gaussian finite mixture models [46]. It should be noted that low-frequency clusters might be a mixture of subclones that contain mutations coming from numerous parallel lineages growing neutrally [32, 68]". Furthermore, we added the following sentence to mention the depth issue in the Introduction: "In general, high-depth sequencing improves subclonal reconstruction accuracy and resolution [32].".

Q2. I think it is highly unlikely that the BRCA2 mutations the authors identify contribute to metastasis in CRC. To probe this further the authors should assess the functional impact of the mutation through cbioportal or the like and assess the copy number zygosity around that locus, appearance of LOH may give some further evidence that these mutations are functional. In general, there is little evidence that BRCA2 has a role in CRC development, see Jonsson et al. Nature 2019.

[Response]: Thanks for your valuable comments. We identified two nonsynonymous mutations in BRCA2 in metastases samples of V824 and V930 (chr13:32954205:A:T and chr13: 32911442:-:A), both of which are heterozygous (without LOH) and located in amplified regions. However, we agree that there is no strong evidence that demonstrates BRCA2 mutations contribute to CRC metastasis yet. To be more rigorous, we revised this part in "Results-Mutational landscape of MRS studies" section as follow: "Interestingly, heterozygous BRCA2 mutations were private to distant metastases, including the lung metastases (LU) and brain metastases (BM) of two patients (V824 and V930), while there is currently no strong evidence that shows BRCA2 mutations are associated with CRC metastasis."

Q3. I would like to see some comparison of the signature deconvolution with other methods to ensure that the authors implementation is approximately equivalent to other widely used tools.

[Response]: Thank you very much for the insights. We, therefore, addressed this deficiency by comparing the performance of figSignatures function against other three signature deconvolution methods. We provided additional files (Figure S3 and Table S3) and described the details of this analysis in the "Results-Temporal dissection of mutational signatures" section as follow: "Therefore, we developed the fitSignatures function to calculate the contribution of well-established signatures to mutations at different levels. By reconstructing the mutational profiles of the HCC and CRC cohorts using 30 COSMIC mutational signatures, we demonstrated that the signature contributions estimated by fitSignatures function were highly similar to those calculated by three other signature deconvolution tools (average Pearson correlation: 1-MutationalPatterns [23]; 0.997-SignatureEstimation [74]; 0.948-deconstructSigs [24]) (Figure S4A and Table S3). The similarities (indicated by cosine similarity) and discrepancies (indicated by RSS) between the original and reconstructed mutational profiles generated with MesKit were also comparable to those generated from other tools (Figure S4B)."

Minor comments

There are a number of grammatical errors that need addressing.

[Response]: Sorry for the grammatical errors. We have sent our revised manuscript to AJE (https://www.aje.cn) asking for improvement of the manuscript.

* The ability to generate phylogenetic trees with different methods is very valuable in my opinion. In the methods the authors comment on how MesKit provides the ability to compare tree similarity using a function called treedist, this is not used in the results as far as I can see. I think including some comparison in the results would be a valuable illustration of how similar (or different) differing approaches may be.

[Response]: Thanks for your valuable suggestion. We agree that results would benefit from a comparison of phylogenetics trees constructed by different methods. We have

Powered by Editorial Manager® and ProduXion Manager® from Aries Systems Corporation

added this analysis in the "Results-Construction and visualization of phylogenetic trees" section as follow: "Moreover, we compared the MP-based phylogenetic trees with those constructed by the NJ method and ML method for each CRC patient. Phylogenetic trees inferred through the three methods shared the same topology and clades for V402, V924 and V953 (Figure S3). When considering branch lengths, the MP-based trees were more similar to the NJ-based trees than the ML-based trees according to KF-branch distance [72] and weighted RF distance [74] (Table S2).". Additional figure (Figure S3) and table (Table S2) were provided for better illustration and explanation.

* It says in the text that the software is released under the GPL v3 License, but in the github repo, an MIT license is shown. This should be clarified.

[Response]: Thank you for pointing this problem out. MesKit was released under the GPL v3 and we have corrected it in the github repository.

* I was able to install the software via devtools but not bioconductor, it appears others have had similar issues - https://github.com/Niinleslie/MesKit/issues/211. Would be good to understand why this might be happening.

[Response]: MesKit was released on the version 3.12 of Bioconductor, which matches R 4.0.3. This problem would be solved when you update Bioconductor to version 3.12 (or higher). Besides, we have made this clear in the revised documentation.

* In the introduction, I think several commonly used ITH related tools are missing citations such as pyclone, DPclust and phylowgs.

[Response]: Thanks for pointing this problem out. Accordingly, we included corresponding citations and revised the Introduction as follows: "…In addition, lots of methods infer tumor heterogeneity by assessing the complex subclonal structure of tumors. Approaches such as SciClone [26], PhyloSub [27] and PyClone [28] are based solely on point mutations (sSNVs or INDELs), while SCHISM [29], DPClust [30] and PhyloWGS [31] adjust for CNAs in their models in different ways. In general, high-depth sequencing improves the accuracy of subclonal reconstruction and resolution [32]"

* This is not mentioned in the paper, but in the R package the authors provide the ability to perform a "neutrality test" as described in my paper from 2016: Williams et al Nature Genetics. This analysis has been superseded by a new software tool called MOBSTER (https://github.com/caravagnalab/mobster), I think this should be made clear in the vignette so that users know what the current recommended analysis tools are for this type of analysis. I would not expect a new implementation of MOBSTER, just a note in the vignette and function documentation would suffice.

[Response]: Thanks for your suggestion. At the beginning of the project, there was no such software or package providing such analysis. This function was retained during the development periods. We have made it clear in the vignette and added proper citation accordingly.

* Hu et al. Nature Genetic should be cited when describing Fst, this was the first (only?) study to use this measure to my knowledge and should be cited accordingly.

[Response]: Thanks for your comments. We have added the citation accordingly.

Reference for point-by-point response

1.Sun R, Hu Z, Sottoriva A, Graham TA, Harpak A, Ma Z, et al. Between-region genetic divergence reflects the mode and tempo of tumor evolution. Nat Genet. 2017;49 7:1015-24. doi:10.1038/ng.3891.

2.Mayakonda A, Lin D-C, Assenov Y, Plass C and Koeffler HP. Maftools: efficient and comprehensive analysis of somatic variants in cancer. Genome research. 2018;28 11:1747-56. doi:10.1101/gr.239244.118.

3.Patel AP, Tirosh I, Trombetta JJ, Shalek AK, Gillespie SM, Wakimoto H, et al. Single-

Powered by Editorial Manager® and ProduXion Manager® from Aries Systems Corporation

	cell RNA-seq highlights intratumoral heterogeneity in primary glioblastoma. Science. 2014;344 6190:1396-401. doi:10.1126/science.1254257.<br>4.Miller CA, White BS, Dees ND, Griffith M, Welch JS, Griffith OL, et al. SciClone: inferring clonal architecture and tracking the spatial and temporal patterns of tumor evolution. PLoS Comput Biol. 2014;10 8:e1003665. doi:10.1371/journal.pcbi.1003665.<br>5.Nik-Zainal S, Van Loo P, Wedge DC, Alexandrov LB, Greenman CD, Lau KW, et al. The life history of 21 breast cancers. Cell. 2012;149 5:994-1007. doi:10.1016/j.cell.2012.04.023.<br>6.Roth A, Khattra J, Yap D, Wan A, Laks E, Biele J, et al. PyClone: statistical inference of clonal population structure in cancer. Nat Methods. 2014;11 4:396-8. doi:10.1038/nmeth.2883.<br>7.Deshwar AG, Vembu S, Yung CK, Jang GH, Stein L and Morris Q. PhyloWGS: reconstructing subclonal composition and evolution from whole-genome sequencing of tumors. Genome Biol. 2015;16:35. doi:10.1186/s13059-015-0602-8.<br>8.Blokzijl F, Janssen R, van Boxtel R and Cuppen E. MutationalPatterns: comprehensive genome-wide analysis of mutational processes. Genome Med. 2018;10 1:33. doi:10.1186/s13073-018-0539-0.<br>9.Williams MJ, Werner B, Heide T, Curtis C, Barnes CP, Sottoriva A, et al. Quantification of subclonal selection in cancer from bulk sequencing data. Nat Genet. 2018;50 6:895-903. doi:10.1038/s41588-018-0128-6.<br>10.Caravagna G, Heide T, Williams MJ, Zapata L, Nichol D, Chkhaidze K, et al. Subclonal reconstruction of tumors by using machine learning and population genetics. Nat Genet. 2020;52 9:898-907. doi:10.1038/s41588-020-0675-5.
**Additional Information:**	
**Question**	**Response**
Are you submitting this manuscript to a special series or article collection?	No
**Experimental design and statistics**<br><br>Full details of the experimental design and statistical methods used should be given in the Methods section, as detailed in our Minimum Standards Reporting Checklist. Information essential to interpreting the data presented should be made available in the figure legends.<br><br>Have you included all the information requested in your manuscript?	Yes
**Resources**<br><br>A description of all resources used, including antibodies, cell lines, animals and software tools, with enough information to allow them to be uniquely identified, should be included in the Methods section. Authors are strongly encouraged to cite Research Resource Identifiers (RRIDs) for antibodies, model organisms and tools, where possible.	Yes

Powered by Editorial Manager® and ProduXion Manager® from Aries Systems Corporation

Have you included the information requested as detailed in our Minimum Standards Reporting Checklist?	
**Availability of data and materials**<br><br>All datasets and code on which the conclusions of the paper rely must be either included in your submission or deposited in publicly available repositories (where available and ethically appropriate), referencing such data using a unique identifier in the references and in the "Availability of Data and Materials" section of your manuscript.<br><br>Have you have met the above requirement as detailed in our Minimum Standards Reporting Checklist?	Yes

Powered by Editorial Manager® and ProduXion Manager® from Aries Systems Corporation

Manuscript

Click here to access/download;Manuscript;MesKIT_forProduction.docx

MesKit: a tool kit for dissecting cancer evolution of multi-region tumor biopsies through somatic alterations

Mengni Liu[1,2,#], Jianyu Chen[1,#], Xin Wang[1], Chengwei Wang[1], Xiaolong Zhang[2], Yubin Xie[1], Zhixiang Zuo[2], Jian Ren[1, 2, *], Qi Zhao[2,*]

[1]*School of Life Sciences, Sun Yat-sen University, Guangzhou, Guangdong 510275, China.*

[2]*State Key Laboratory of Oncology in South China, Collaborative Innovation Center for Cancer Medicine, Sun Yat-sen University Cancer Center, 651 E Dongfeng Road, Guangzhou, Guangdong, 510060, China*

[*]**Corresponding authors:** Qi Zhao. Tel/Fax: +86-20-87342209; E-mail: zhaoqi@sysucc.org.cn; ORCID: https://orcid.org/0000-0002-8683-6145 and Jian Ren. E-mail: renjian.sysu@gmail.com; ORCID: https://orcid.org/0000-0002-4161-1292

[#]Contributed equally.

Abstract

Background

Multi-region sequencing (MRS) has been widely used to analyze intra-tumor heterogeneity (ITH) and cancer evolution. However, comprehensive analysis of mutational data from MRS is still challenging, which requires complicated integration of a plethora of computational and statistical approaches.

Findings

Here, we present MesKit, an R/Bioconductor package, that can assist in characterizing genetic ITH and tracing the evolutionary history of tumors based on somatic alterations detected by MRS. MesKit provides a wide range of analysis and visualization modules, including ITH evaluation, metastatic route inference, and mutational signature identification. In addition, MesKit implements an auto-layout algorithm to generate phylogenetic trees based on somatic mutations. The application of MesKit for two reported MRS datasets of hepatocellular carcinoma (HCC) and colorectal cancer (CRC) identified known heterogeneous features and evolutionary patterns, together with potential driver events during cancer evolution.

Conclusions

In summary, MesKit is useful for interpreting ITH and tracing evolutionary trajectory based on MRS data. MesKit is implemented in R and available at https://bioconductor.org/packages/MesKit under the GPL v3 license.

Keywords: multi-region sequencing; somatic alterations; intra-tumor heterogeneity; metastatic routes; phylogenetic tree

Introduction

Cancer evolves through a process of somatic alterations [1], of which spatial and/or temporal changes can be detected by multi-region sequencing (MRS). Currently, MRS has become an effective and affordable way to trace the evolutionary history of carcinogenesis and metastasis. Cancer evolution research is focused on the identification and estimation of intra-tumor heterogeneity (ITH), phylogenetic reconstruction, mutational signature analysis, etc. Numerous MRS studies have identified extensive ITH among many solid tumors originating in the liver, prostate, esophagus, breast, and lung [2-7]. In addition, increased ITH has been implicated in dismal cancer prognosis [8-10]. While recent studies have largely generated descriptive summaries of ITH, a quantitative understanding of the heterogeneity within and between tumors from the same patient is more informative for personal therapeutics.

Recently, plenty of MRS studies have employed phylogenetic trees to show the temporal sequence and heterogeneous divergence between samples [2, 11, 12]. There are also increasing efforts to reconstruct subclonal phylogenies via a ‘clone tree’, which summarizes lineage relationships between cellular subpopulations [13-15]. Phylogenetic reconstruction over the cancer cell fraction (CCF) estimates has identified both monoclonal and multiclonal seeding patterns in several cancers [3, 16-18]. The distinction between these two patterns may have important clinical implications [19], it is thus necessary to infer metastatic routes and to explore potential metastasis drivers.

Moreover, MRS provides insights into the dynamics of mutational processes during tumor progression. A previous study indicated that DNA damage repair dysfunction might be crucial for mutation accumulation during osteosarcoma evolution [20]. Recently, Yan et al. [21] performed MRS of tumors from 39 esophageal squamous cell carcinoma (ESCC) patients and identified several potential actionable targets, such as *EGFR* and *FGFR1*. They also showed that APOBEC mutations and aging predominated in the early stage of tumorigenesis of ESCC. These findings suggest that the MRS strategy has the potential to reveal mutational mechanisms and thereby could improve both diagnosis and treatment.

The downstream analysis of MRS data focuses on somatic alterations, including somatic single-nucleotide variants (sSNVs), small insertions and deletions (INDELs) and copy-number alterations (CNAs). At present, many tools are available to analyze somatic alterations, which has greatly

promoted the development of cancer genomics. For example, Maftools [22] provides multiple functions for pathway annotation, de novo signature and enrichment analysis. MutationalPatterns [23] and deconstructSigs [24] are powerful tools for exploring mutational patterns and identifying mutational signatures of a single tumor sample. Besides, Palimpsest [25] enables the identification of different mutational signatures between clonal and subclonal mutations. In addition, lots of methods infer tumor heterogeneity by assessing the complex subclonal structure of tumors. Approaches such as SciClone [26], PhyloSub [27] and PyClone [28] are based solely on point mutations (sSNVs or INDELs), while SCHISM [29], DPClust [30] and PhyloWGS [31] adjust for CNAs in their models in different ways. In general, high-depth sequencing improves the accuracy of subclonal reconstruction and resolution [32]. However, performing integrated mutational analysis of MRS using these tools is inconvenient, as different preprocessing steps and inconsistent input formats are required. On the other hand, it is laborious and time-consuming to generate publication-quality images such as mutational profiles and phylogenetic trees, which requires manual modifications using extra graphic editors.

To address these concerns, we present MesKit, an R/Bioconductor package that provides commonly used analysis and visualization modules for MRS studies. MesKit was designed as an easy-to-use R package that only requires a Mutation Annotation Format (MAF) file and a clinical file as inputs, enabling researchers to evaluate the contribution of point mutations to heterogeneity within/between tumors from the same patient. MesKit can also be used to depict mutational profiles, track evolutionary dynamics and characterize mutational patterns at different levels. Notably, we implemented an auto-layout algorithm to visualize rooted phylogenetic trees with annotations. In addition, MesKit enables easy integration and analysis of segmentation data and CCF data and a Shiny application is provided to facilitate interactive analysis. Finally, we applied MesKit on two high-quality MRS datasets of hepatocellular carcinoma (HCC) [2] and colorectal cancer (CRC) [12] (Table S1). We reproduced well-known heterogeneous features and evolutionary patterns, together with potential driver events of HCC and CRC, demonstrating the robustness of MesKit in interpreting ITH and to inferring evolutionary trajectories based on MRS data.

Materials and methods

Data collection and preprocessing

We used two cohorts in our analysis. The HCC cohort included tumor tissue (n = 52) and matched blood (germline, n = 11) samples from 11 patients, which were collected before treatment [2]. All samples were sequenced using whole exome sequencing (average depth of 158X) and re-analyzed with a uniform pipeline described below. In brief, we performed sSNV calling for each tumor/normal pair with Mutect (version 1.1.7) [33], while INDELs were detected with Strelka v2.7.1 [34]. Additionally, we adopted the "force calling" method [35] to rescue potential real mutations for each sample based on the aggregate set of somatic events in each patient using *samtools mpileup* (version 1.2) [36]. Both sSNVs and INDELs were annotated through ANNOVAR (v.20191024) [37]. The following filters were further applied to identify the sSNVs and INDELs: (1) Mutations with fewer than 15 total reads or 5 variant reads were discarded. (2) Mutations listed in dbSNP147 were removed unless they were documented in the Catalogue of Somatic Mutations in Cancer (COSMIC) database. (3) Mutations listed in the National Heart, Lung, and Blood Institute Exome Sequencing Project were removed. Copy number analysis based on exome sequencing was performed using Sequenza v3.0.0 [38]. Segments smaller than 500 kb were filtered and only autosomes were used in copy number analysis. CCFs of mutations were estimated by PyClone (v0.13.0) [28], which adjusted the variant allele frequencies (VAFs) of somatic mutations based on local copy numbers of the mutated loci and tumor purity (Sequenza v3.0.0) [38]. The second cohort (the CRC cohort) comprised six patients processed with MRS for paired primary tumors and metastases (3–5 regions each) [12]. We obtained somatic mutation calls for sSNVs and INDELs, copy number segment data and CCF estimates of mutations from the original study. Driver genes of HCC and CRC were defined by IntOGen (v.2020.2). The GISTIC2 results of the TCGA HCC and TCGA CRC projects were obtained from the Broad GDAC website (analysis stamp: 2016_01_28).

Clonal status of somatic mutations

Since multiple samples collected from a single tumor collectively reflect its clonal composition, these regions should be considered as a whole to capture the overall tumor dynamics. Here, we assume that

each tumor follows neutral exponential growth in a well-mixed population [39, 40]. When MRS data are available, the merged CCF (CCF_{merged}) of each mutation is computed by integrating multiple regions as previously described [12, 41, 42]:

$$CCF_{merged} = \begin{cases} \frac{\sum_{i=1}^{k} CCF_i \times d_i}{\sum_{i=1}^{k} d_i} & CCF < 1 \\ 1 & CCF \geq 1 \end{cases} \quad (1)$$

where d_i and CCF_i refer to the sequencing depth and CCF estimation in region i, respectively. The clonal status of sSNVs/INDELs are determined based on CCFs. A CCF value of 1 indicates that the mutation is present in 100% of the cancer cells in a sample, while a CCF value < 1 indicates that the mutation is present in a subset of the cancer cells in a sample and thus is subclonal. In each sample, a mutation is classified as clonal with upper bound of the 95% confidence interval (CI) of the CCF is ≥ 1; and otherwise subclonal [43]. For MRS data, a mutation is considered subclonal when all of the following criteria are satisfied: (1) at least one region with upper bound of 95% CI of the CCF < 1; (2) at least one region with CCF < 0.5; (3) CCF_{merged} of mutation m < 0.5 (the cut-off was chosen for its good performance in defining subclonality in simulated virtual tumors [44, 45].

Estimation of ITH

MesKit includes several measures of ITH defined by recent genomic studies. For a single region/tumor, it is common to infer subpopulations of tumor cells by clustering VAFs or CCFs [26, 30]. To implement this process, we employed Gaussian finite mixture models for one-dimensional clustering of VAFs or CCFs using mclust R package [46]. As copy number gains and losses can alter the fraction of reads bearing a mutation, we only focused on heterozygous mutations within copy-number neutral and loss of heterozygosity (LOH)-free regions when clustering VAFs. More recently, Mroz et al. [47] developed the mutant-allele tumor heterogeneity (MATH) index, which corresponds to the ratio of the median absolution deviation (MAD) and the median of the VAF values among tumor-specific mutated loci. Generally, a more heterogeneous tumor with a higher MATH score tends to have a wider distribution of VAFs among all mutation loci and centers at a lower fraction.

$$MATH = 1.4826 \times \frac{MAD(VAF)}{Median(VAF)} \quad (2)$$

Another approach to estimate ITH is calculating the area under the curve (AUC) of the cumulative density function based on the CCFs per tumor, and tumors with higher AUC values are considered to be more heterogeneous [48]. Moreover, to quantify the genetic divergence of ITH between regions or tumors, we introduced two classical metrics derived from population genetics, Wright's fixation index (F_{ST}) [49] and Nei's genetic distance [50]. Calculations of between-region genetic heterogeneity within tumors only consider subclonal mutations, as clonal mutations present in all regions do not contribute to ITH. For pairwise comparisons of heterogeneity between tumors, both clonal and subclonal mutations were taken into consideration. The F_{ST} index estimating between-region ITH for k regions was computed as described previously [44]:

$$F_{ST} = \frac{1}{r} \times \sum_{j=1}^{r} F_{ST_j}^{Hudson},\ r = \binom{k}{2} \tag{3}$$

$$F_{ST_j}^{Hudson} = \frac{\sum_{m=1}^{m_t} (f_a^m - f_b^m)^2 - \frac{f_a^m \times (1 - f_a^m)}{d_a^m - 1} - \frac{f_b^m \times (1 - f_b^m)}{d_b^m - 1}}{\sum_{m=1}^{m_t} f_a^m \times (1 - f_b^m) + f_b^m \times (1 - f_a^m)} \tag{4}$$

where m_t represents the total number of sSNVs in regions a and b, f_a^m denotes the VAF for sSNV m and d_a^m denotes the sequencing depth for sSNV m in region a.

Nei's genetic distance for k regions within the same tumor was defined as follows [50]:

$$D_{Nei} = \frac{1}{r} \times \sum_{j=1}^{r} D_{Nei_j},\ r = \binom{k}{2} \tag{5}$$

$$D_{Nei_j} = -log \frac{\sum_{m=1}^{m_t} ccf_a^m \times ccf_b^m + (1 - ccf_a^m)(1 - ccf_b^m)}{\sqrt{(\sum_{m=1}^{m_t} {ccf_a^m}^2 + (1 - ccf_a^m)^2) \times (\sum_{m=1}^{m_t} {ccf_b^m}^2 + (1 - ccf_b^m)^2)}} \tag{6}$$

where m_t represents the total number of sSNVs in regions a and b. ccf_a^m and ccf_b^m represent the CCF values in region a and region b for mutation m, respectively.

Inference of metastatic routes

For spatially separated lesions from the same patient, the potential metastatic route can be determined by comparing subclonal architecture between paired lesions. Here, MesKit integrated a Jaccard similarity index (JSI)-based method to identify seeding patterns based on the CCFs of sSNVs for paired lesions [42]. The Jaccard coefficient for a lesion pair (a, b) is calculated as follows:

$$JSI = \frac{SS_{ab}}{PC_a + PC_b + SS_{ab}} \tag{7}$$

where SS_{ab} and PC_a/PC_b represent shared subclonal sSNVs of lesion pair (a, b) and private clonal sSNVs of lesion a/b, respectively. The mean SS_{ab}, and PC_a/PC_b of all sample pairs from lesion a and lesion b are used to compute the JSI for lesions with MRS data.

Construction and visualization of phylogenetic trees

MesKit reconstructs the phylogeny of multiple specimens from individual patients based on the presence or absence of somatic mutations. This process is implemented in *getPhyloTree* function via utilization R implementations of several standard phylogenetic approaches from the APE [51] and PHANGORN [52] R packages, including distance-based methods (neighbor-joining (NJ) [53] and minimum evolution [54]) as well as character-based methods (maximum parsimony (MP) [55] and maximum likelihood (ML) [56]). Notably, we implemented an auto-layout algorithm via the *plotPhyloTree* function to generate customizable images of phylogenetic trees with annotations (File S1). Furthermore, by employing the *treedist* function from the PHANGORN [52] R package, MesKit enables the comparison of phylogenetic trees constructed by different methods via the *compareTree* function.

Mutational signature analysis

To illustrate the dynamic mutational spectrum during tumor progression, we implemented mutational signature analysis based on phylogenetic trees. The process starts with the construction of a mutation matrix accounting for 96 trinucleotide changes, where the sequence context of the base substitutions can be retrieved from the corresponding reference genome using the BSgenome R package. Six types of base substitution types are distinguished by convention: C>A, C>G, C>T, T>A, T>C, and T>G. As methylated cytosine at CpG sites with the attendant risk of spontaneous deamination are mutagenic hotspots in the human genome [57], C>T mutations can be divided into C>T at CpG sites and other sites [23]. Genomic mutations are temporally dissected into truncal (shared among all samples from the same patient) and branch mutations of phylogenetic trees. For each mutational type, Fisher's exact test is implemented to assess the difference between the truncal and branch mutations. Once the signature matrix is provided, the *fitSignatures* function estimates the optimal contributions of known

signatures to reconstruct a mutational profile, which minimizes the residual sum of squares (RSS) between the original and reconstructed mutational profiles. This process was implemented by integrating a non-negative least-squares (NNLS) algorithm using the pracma R package (https://CRAN-project.rg/package=-pracma), as previously described in MutationalPatterns [23]. For convenience, we included known signature matrices (published by Alexandrov et al. in 2013 and Cosmic version 2, 3) along with the proposed etiology in MesKit. The similarity between mutational profiles A and B is calculated by cosine similarity as follows:

$$sim(A,B) = \frac{\sum_{i=1}^{n} A_i B_i}{\sqrt{\sum_{i=1}^{n} A_i^2}\sqrt{\sum_{i=1}^{n} B_i^2}} \tag{8}$$

where mutational profiles A and B are non-zero vectors with n mutational types. Cosine similarity value can be utilized to test how well each mutational profile can be explained by the provided mutational signatures. Two mutational profiles are identical when the cosine similarity is 1, and are independent when the cosine similarity is 0.

Results

Overview of MesKit functions and implementation

MesKit was implemented as an open source R/Bioconductor package. With a MAF file and a clinical data file as standard inputs, MesKit provides a series of analysis and visualization functions to interpret mutational data from MRS experiments (**Figure 1**). In addition, we implemented a Shiny application to facilitate the usage of the package. Moreover, we built a Docker image that enables the deployment of the Shiny-based MesKit GUI in a C/S mode.

Mutational landscape of MRS studies

Generally, somatic mutations identified from MRS in a single tumor are classified as “public mutations” (existing in all regions of the tumor), “shared mutations” (existing in part of all regions), or “private mutations” (existing in a single region) [20, 44, 58]. Such spatial-mutation categories largely correspond to the temporal order of mutation genesis during tumor evolution: most public mutations occur early in tumor-initiating cells and are inherited by their offspring, whereas private mutations

accumulate sporadically and markedly increase the ITH among different patients [59]. In MesKit, we implemented the *classifyMut* function to help categorize somatic mutations based on regional distribution, and/or to identify clonal and subclonal mutations according to their estimated CCFs (Methods). Analysis of the HCC and CRC cohorts showed significant inter-individual heterogeneity but much less intra-individual heterogeneity (**Figure 2** and Figure S1). In line with previous findings [13, 60, 61], the primary tumors and metastases of the CRC cohort exhibited high genomic concordance (Figure 2A). As expected, public mutations harbor higher CCFs than private mutations (Figure S2), which were more likely to be clonal events. Recurrent mutations in putative driver genes of CRCs (defined by IntOGen v.2020.2), such as *KRAS* and *APC*, were clonal and shared between paired primary tumors and metastases, indicating their early occurrence in colorectal carcinogenesis (Figure 2A and Figure S2). Interestingly, heterozygous *BRCA2* mutations were private to distant metastases, including the lung metastases (LU) and brain metastases (BM) of two patients (V824 and V930), while there is currently no strong evidence that shows *BRCA2* mutations are associated with CRC metastasis. In addition, the *plotCNA* function of MesKit can be used to characterize the CNA landscape across samples based on copy number data. Consistent with TCGA projects and other previous studies of hepatocellular carcinoma [62, 63], a number of copy number alterations were observed in our HCC cohort, such as gains of 1q, 6p, 8q and 13q as well as losses of 1p, 4q, 9q and 11q (Figure 2B). Taken together, these data suggest that MesKit can easily characterize the mutational landscape and potential driver genes during cancer evolution.

ITH estimation

Understanding the degree and development of ITH is clinically important, as ITH has been associated with treatment resistance and the prognosis of cancer patients [64]. MesKit integrates several approaches to estimate ITH within and between regions/tumors from the same patient. In MesKit, the mut*Cluster* function deduces distinct subpopulations of a sample/tumor by clustering VAFs/CCFs in one dimension based on Gaussian finite mixture models [46]. It should be noted that low-frequency clusters might be a mixture of subclones that contain mutations coming from numerous parallel lineages growing neutrally [32, 65]. Another approach is calculating MATH score, which is positively

correlated with tumor heterogeneity and metastatic potential [66, 67]. Besides, we integrated an index described by Charoentong et al. [48], to assess ITH by calculating the AUC of the cumulative density function from all CCFs per sample/tumor. Samples/tumors with higher AUCs are considered to be more heterogeneous than those with lower AUCs. Applying these measures on HCC8010 showed that samples with wider distributions of VAFs tended to have higher MATH scores, and VAF-based ITH was comparable to that calculated by CCFs (Figure 3A, B). Moreover, we introduced two measures from population genetics [44, 49, 50], named F_{ST} and Nei's genetic distance, to enable pairwise comparisons between regions/lesions. Comparison of ITH between primary tumors and paired metastases in CRC cohort showed no significant difference using these two indices (Wilcoxon signed-rank test, F_{ST}: $P = 0.5781$, Nei's distance: $P = 0.1094$, Figure 3C). Similarly, this observation supports the conclusion that primary and metastatic tumors of CRC exhibit a high degree of mutational discordance.

Inferring the clonality of metastatic seeding

Since metastasis is the major cause of cancer-related death, it is particularly important to gain a systematic understanding of how tumor cells disseminate and the scale of ongoing parallel evolution in metastatic and primary sites [68]. Given that mutations with similar CCFs tend to cluster into the same subpopulation [69, 70], many studies inferred the potential metastatic routes between different lesions from the same patient by plotting CCFs of mutations [12, 42, 71]. By this means, Xue et al. [71] identified both monoclonal and multiclonal origins of separate type combined hepatocellular and intrahepatic cholangiocarcinoma (cHCC-ICC). Here, we developed the *compareCCF* function to calculate the merged CCFs of distinct lesions with MRS data. To visualize the seeding patterns between lesions in a more intuitive way, the results of this function can be further used to plot CCF plots, where the clusters at (1, 1) correspond to the clonal mutations present in all cells in both lesions (CCF = 1), while those on axes refer to lesion-private subclones. In addition, MesKit integrated a JSI-based method to calculate mutational similarity between lesions [42]. Pairs following polyclonal seeding generally achieve higher JSI values because of their higher proportion of shared subclonal sSNVs and fewer lesion-private sSNVs (Methods). Analysis of the CRC cohort with these functions

revealed that all brain metastases exhibited enrichment of metastasis-private clonal sSNVs and shared clonal sSNVs, but lacked shared subclonal sSNVs (Figure 3D). Moreover, all brain metastases comprised a single phylogenetic clade in the phylogenetic trees (Figure S4). These observations jointly indicated that the brain metastases of this CRC cohort followed a monoclonal seeding manner, consistent with the original study [12]. Besides, in both paired primaries and metastases of most CRCs, the merged CCFs of mutations in CRC driver genes including *APC, KRAS* and *TP53* were > 0.6, suggesting that they may contribute to CRC tumorigenesis and metastasis. Notably, lymph nodes showed higher JSI values than distant metastases in V750 and V824, indicating polyclonal seeding was more prevalent in lymph node metastases (Figure 3D). In summary, these results demonstrated the ability and efficiency of MesKit to identify distinct patterns of seeding between paired lesions.

Construction and visualization of phylogenetic trees

A systematic understanding of the evolutionary relationships among tumor regions from a single patient plays a fundamental role in MRS studies, with the phylogenetic tree being a primary tool for delineating the relationship between tumor regions and interpreting ITH [2, 11, 44]. Consistent with original studies, we applied the MP method to reconstruct the tumor phylogeny of the CRC cohort using the *getPhyloTree* function in MesKit. Phylogenetic trees were further visualized with the function *plotPhyloTree*, which provides options to color the branches according to the classification of mutations or putative known signatures. We consistently reproduced tree structures of most CRCs from the original study [12], in which the primary regions and metastatic regions were clearly separated (**Figure 4**). Inspection of the phylogeny indicated early divergence of the metastatic lineage in V402, V824, V930, V953 and V974, whereas divergence occurred during diversification of the primary tumor in V750. Moreover, we compared the MP-based phylogenetic trees with those constructed by the NJ method and ML method for each CRC patient. Phylogenetic trees inferred through the three methods shared the same topology and clades for V402, V924 and V953 (Figure S3). When considering branch lengths, the MP-based trees were more similar to the NJ-based trees than the ML-based trees according to KF-branch distance [72] and weighted RF distance [74] (Table S2).

Collectively, these results demonstrate the functionality and efficiency of MesKit for analyzing and visualizing tumor phylogeny.

Temporal dissection of mutational signatures

Analysis of mutational signatures can be used to understand the mechanisms of transformation of normal cells to malignant cells and to identify underlying risk factors for tumor development. First, Alexandrov et al. [73] utilized over 7,000 cancer genomes and exomes to identify 21 signatures across 30 tumor types. More recently, the Wellcome Trust Sanger Institute (http://cancer.sanger.ac.uk/cosmic/signatures) published 30 mutational signatures (version 2) in primary cancer and an expanded 67 single base substitution signatures (version 3). Considering the limited number of tumor samples assessed by MRS and thus the limited number of identified mutations, it is not amenable to conduct de novo signature extraction. Therefore, we developed the *fitSignatures* function to calculate the contribution of well-established signatures to mutations at different levels. By reconstructing the mutational profiles of the HCC and CRC cohorts using 30 COSMIC mutational signatures, we demonstrated that the signature contributions estimated by *fitSignatures* function were highly similar to those calculated by three other signature deconvolution tools (average Pearson correlation: 1-MutationalPatterns [23]; 0.997-SignatureEstimation [74]; 0.948-deconstructSigs [24]) (Figure S4A and Table S3). The similarities (indicated by cosine similarity) and discrepancies (indicated by RSS) between the original and reconstructed mutational profiles generated with MesKit were also comparable to those generated from other tools (Figure S4B). As shown in Figure 5B, hierarchical clustering via Euclidean distance of the patients based on their cosine similarity values clearly separated the HCCs from the CRCs. These results demonstrate the ability of MesKit to reliably estimate signature contributions. We further applied the *fitSignatures* function with 30 COSMIC signatures to truncal and branch sSNVs of HCC5647, HCC7608 and HCC8716 (other HCCs were excluded for their truncal/branch sSNVs were less than 50). All three HCCs exhibited a prominent decrease of the contribution of signature 22 (exposures to aristolochic acid) in branch mutations compared with truncal mutations (**Figure 5A** and Table S4) Among them, HCC5647 and HCC8716 showed significantly higher percentages of T>A ($P < 0.01$) in truncal mutations than branch mutations

(Figure S5), which is consistent with the characteristic patterns of the signature 22 (characterized by T>A). Considering these observations, we hypothesized that exposure to aristolochic acid contributed significantly to mutagenic process in the early stage of tumorigenesis for these HCCs. This analysis suggests the utility of MesKit to reveal the dynamic mutational processes.

Discussion

Multi-region sequencing has become an affordable and effective way to investigate genetic heterogeneity and trace tumor evolutionary trajectory. Multiple spatial snapshots of tumors can help reduce sampling bias and detect minor subclones. Despite these advantages, there are few tools available to systematically analyze mutational data of multi-region samples from a single patient so far. In this regard, we present MesKit, an R/Bioconductor package, that incorporates a diversity of essential analysis and visualization functions for MRS studies. MesKit quantifies ITH based on somatic mutations by integrating several approaches described in recent cancer genome studies [47-50]. Besides, MesKit can be used to infer metastatic routes, characterize mutational patterns at different levels, and generate publication-quality images such as mutational profiles and phylogenetic trees. Via implementation of the Shiny application, MesKit enables researchers with minimal informatics skills to effortlessly interpret and visualize the intricate mutational data from MRS. Furthermore, we demonstrated the utility and efficiency of MesKit in interpreting ITH and inferring evolutionary trajectory using two published MRS datasets of HCC and CRC. Collectively, we believe that MesKit is a handy and feature-rich tool, which will greatly facilitate the exploration of mutational data from MRS experiments.

As MesKit takes a MAF file and a clinical data file as standard inputs, it primarily evaluates ITH based on somatic mutations, and its assessment of contributions of CNAs is still limited. Currently, several subclonal reconstruction methods are available to infer the relative order of occurrence between a SNV and its associated CNA. In future updates, we plan to implement the integration of results from these methods to provide insights into the clonality and temporal dynamics of ITH. On the other hand, as ITH arises through various mechanisms, it is invaluable to perform investigations at the genetic, transcriptomic, phenotypic, and cellular levels.

Availability of source code and requirements

Project name: MesKit

Project home page: https://github.com/Niinleslie/MesKit

Operating system(s): Platform independent

Programming language: R

Other requirements: R ≥ 4.0

License: GPL-3

RRID: SCR_020959

biotools: meskit

The code for creating the figures in this article can be found and re-executed in a Code Ocean capsule [75]. Supporting data and an archival copy of the code is also available via the *GigaScience* database GigaDB [76].

Abbreviations

AUC: area under the curve; BM: brain metastasis; CCF: cancer cell fraction; CI: confidence interval; CNAs: copy-number alterations; COSMIC: catalogue of somatic mutations in cancer; CRC: colorectal cancer; ESCC: esophageal squamous cell carcinoma; F_{ST}: fixation index; HCC: hepatocellular carcinoma; INDELs: small insertions and deletions; ITH: intra-tumor heterogeneity; JSI: jaccard similarity index; LN: lymph node; LOH: losses of heterozygosity; LU: lung metastasis; MAD: median absolution deviation; MAF: mutation annotation format; MATH: mutant-allele tumor heterogeneity; ML: maximum likelihood; MP: maximum parsimony; MRS: multi-region sequencing; NJ: neighbor-joining; NNLS: non-negative least squares; RSS: residual sum of squares; sSNVs: somatic single-nucleotide variants; VAFs: variant allele frequencies; WES: whole exome sequencing

Authors' contributions

QZ and JR conceived the project. ML, JC, XW, and CW developed the methodology and implemented the method. LZ and YX helped test the software. ML, QZ and JR wrote the paper. All authors read and approved the final manuscript.

Competing interests

The authors have declared no competing interests.

Acknowledgements

This work was supported by grants from the National Natural Science Foundation of China (Grant Nos. 91753137, 31471252, 31771462, 81772614, U1611261 and 31801105); National Key R&D Program of China (Grant No. 2017YFA0106700); Program for Guangdong Introducing Innovative and Entrepreneurial Teams (Grant No. 2017ZT07S096); Guangdong Natural Science Foundation (Grant No. 2018A030313323); and Fundamental Research Funds for the Central Universities (SYSU: 19ykpy184).

Figure legends

Figure 1. Overview of the MesKit package

A. Overview of MesKit. MesKit consists of five major modules: characterizing mutational landscape, estimating ITH, inferring metastatic routes, exploring mutational patterns, and visualizing phylogenetic trees automatically. Corresponding functions for each module are displayed separately.

Figure 2. Mutational landscape of the HCC and CRC cohorts

A. Mutational profile of the CRC cohort. The oncoprint of the top 15 most frequently mutated driver genes of CRC grouped by public, shared or private mutations, including both clonal and subclonal drivers. Genes were sorted by mutational frequency, and those with multiple mutations were annotated as Multi_Hit. Samples were split by patients as indicated by the annotation bar (bottom). P, primary tumor; BM, brain metastasis; LN, lymph node metastasis; LU, lung metastasis. The stacked bar charts on the top and right show the number of different types of mutations per sample and per driver gene, respectively. **B**. The consistent CNAs of the HCC cohort with significant recurring CNAs were identified from the TCGA hepatocellular carcinoma project by GISTIC2.0 (obtained from the Broad GDAC website). Each track represents one tumor sample. Dark red indicates amplifications ($CN \geq 4$), light red indicates gains ($2 < CN < 4$), dark blue indicates deletions ($CN = 0$), and light blue indicates losses ($0 < CN < 2$).

Figure 3. ITH estimation and the clonality of metastatic seeding

A. Clustering mutations by VAFs of each tumor sample from HCC8010 based on a Gaussian finite mixture model. MATH scores are indicated above. **B**. CCF density plot of tumor samples from HCC8010. **C**. F_{ST}- and Nei's distance-based quantification of ITH in paired primary tumors and metastases of the CRC cohort (n = 7). P-value, Wilcoxon rank-sum test (two-sided). **D**. Density plots of merged CCF values in paired primary tumors and metastases of the CRC cohort. For each pair, the JSI was computed according to equation (7). Putative CRC driver genes are indicated on the plot. P, primary tumor; BM, brain metastasis; LN, lymph node metastasis; LU, lung metastasis.

Figure 4. Phylogenetic trees of the CRC cohort

Phylogenetic trees of the CRC cohort were constructed from all sSNVs and INDELs using the MP algorithm. Branches were colored according to the regional distribution of mutations. The branch lengths are proportional to the number of mutations.

Figure 5. Temporal dissection of mutational signatures

A. Relative contribution of the 96 trinucleotide changes to the original mutational profile (upper panel), the reconstructed mutational profile (middle panel), and the difference between these profiles for truncal mutations and branch mutations from HCC patient HCC8716. The RSS, cosine similarity between the original and the reconstructed mutational profile and proposed etiology for mutational processes underlying the signature are indicated on the top. **B**. Heat map of cosine similarities between the 30 COSMIC signatures and the mutational profiles of the HCC and CRC cohorts. Patients were hierarchically clustered between the vectors of cosine similarities of signatures using the Euclidean distance methods. The signatures were ordered according to hierarchical clustering based on the cosine similarity between signatures.

Supplementary material

Figure S1. Mutational landscape of HCC and CRC cohorts

A. Mutational profile of HCC cohort. Oncoprint of top 15 most frequently mutated driver genes of HCC were grouped by public, shared or private mutations including both clonal and subclonal drivers. Stacked bar charts on the top and right show the number of mutations for different types per sample and per driver gene, respectively. Genes were sorted by mutational frequency and samples were split by patients as indicated by the annotation bar (bottom). **B**. The consistent CNAs of CRC cohort with significant recurring CNAs identified from TCGA Colorectal Adenocarcinoma project by GISTIC2.0 (obtained from Broad GDAC website). Each track represents one tumor sample. P, primary tumor; BM, brain metastasis; LN, lymph node metastasis; LU, lung metastasis. Dark red for amplifications ($CN \geq 4$), light red for gains ($2 < CN < 4$), dark blue for deletions ($CN = 0$), and light blue for losses ($0 < CN < 2$).

Figure 2. CCF heat maps of CRC cohort

The heat maps of CCF values of tumor samples from the same patient. The color bar next to the heatmap indicates the classification of mutations shared amongst different samples. The proportion of each classification is indicated in the legend. Putative CRC driver genes were labelled on the right.

Figure S3. Comparison of phylogenetic trees constructed by different methods of the CRC cohort

Comparison of the MP-based phylogenetic trees against those constructed by NJ method and ML method for each CRC patient. For each pair, the different clades between two phylogenetic trees were highlight in red (the first tree) or blue (the second tree).

Figure S4. Comparison of signature contributions measured by MesKit, MutationalPatterns, SignatureEstimation and deconstructSigs

A. Relative contributions of all 30 COSMIC signatures for each patient in the HCC and CRC cohorts.
B. Cosine similarity and RSS between the original and the reconstructed mutational profiles.

Figure S5. Mutation spectra of truncal and branch mutations of HCC5647, HCC7608 and HCC8716

Stacked bar plots show the proportions of truncal and branch mutations accounted for by each of the six mutation types in HCC5647, HCC7608 and HCC8716. The number of analyzed mutations is displayed on top of each bar. A Fisher exact test was used to compare truncal and branch mutations for each mutation type (two-sided: *P < 0.01).

Figure S6. Schematic diagram of visualizing phylogenetic trees

Node N refers to a non-mutated normal sample: node 0 represents the starting node.
In tree T_0: $K = \{node\ 0, node\ 2, node\ 4, node\ 5, node\ 8\}$, $K^{[1]}$ is $node\ 0$;
$B = \{node\ 1,\ node\ 3,\ node\ 6,\ node\ 7\}$, $B^{[1]}$ is $node\ 1$; $R = \{node\ 1,\ node\ 7\}$, $R^{[1]}$ is $node\ 1$; $L = \{node\ 3, node\ 6\}$, $L^{[1]}$ is $node\ 3$

Table S1. Clinical features of the HCC and CRC cohorts

Table S2. Distance between the MP-based phylogenetic tree and the NJ-/ML- based phylogenetic tree for each patient in CRC cohort

Table S3. Relative contributions of all 30 COSMIC signatures for each patient in HCC and CRC cohorts, as measured by MesKit, MutationalPatterns, SignatureEstimation and deconstructSigs

Table S4. Signature contributions of truncal and branch mutations of HCC5647, HCC7608 and HCC8716

References

1. Hanahan D and Weinberg RA. Hallmarks of cancer: the next generation. Cell. 2011;144 5:646-74. doi:10.1016/j.cell.2011.02.013.
2. Lin DC, Mayakonda A, Dinh HQ, Huang P, Lin L, Liu X, et al. Genomic and Epigenomic Heterogeneity of Hepatocellular Carcinoma. Cancer Res. 2017;77 9:2255-65. doi:10.1158/0008-5472.CAN-16-2822.
3. Gundem G, Van Loo P, Kremeyer B, Alexandrov LB, Tubio JMC, Papaemmanuil E, et al. The evolutionary history of lethal metastatic prostate cancer. Nature. 2015;520 7547:353-7. doi:10.1038/nature14347.
4. Hong MK, Macintyre G, Wedge DC, Van Loo P, Patel K, Lunke S, et al. Tracking the origins and drivers of subclonal metastatic expansion in prostate cancer. Nat Commun. 2015;6:6605. doi:10.1038/ncomms7605.
5. Hao JJ, Lin DC, Dinh HQ, Mayakonda A, Jiang YY, Chang C, et al. Spatial intratumoral heterogeneity and temporal clonal evolution in esophageal squamous cell carcinoma. 1546-1718 (Electronic).
6. Yates LR, Gerstung M, Knappskog S, Desmedt C, Gundem G, Van Loo P, et al. Subclonal diversification of primary breast cancer revealed by multiregion sequencing. Nat Med. 2015;21 7:751-9. doi:10.1038/nm.3886.
7. de Bruin EC, McGranahan N, Mitter R, Salm M, Wedge DC, Yates L, et al. Spatial and temporal diversity in genomic instability processes defines lung cancer evolution. Science. 2014;346 6206:251-6. doi:10.1126/science.1253462.
8. Zhang J, Fujimoto J, Zhang J, Wedge DC, Song X, Zhang J, et al. Intratumor heterogeneity in localized lung adenocarcinomas delineated by multiregion sequencing. Science. 2014;346 6206:256-9. doi:10.1126/science.1256930.
9. Patel AP, Tirosh I, Trombetta JJ, Shalek AK, Gillespie SM, Wakimoto H, et al. Single-cell RNA-seq highlights intratumoral heterogeneity in primary glioblastoma. Science. 2014;344 6190:1396-401. doi:10.1126/science.1254257.

10. Jamal-Hanjani M, Wilson GA, McGranahan N, Birkbak NJ, Watkins TBK, Veeriah S, et al. Tracking the Evolution of Non–Small-Cell Lung Cancer. New Engl J Med. 2017;376 22:2109-21. doi:10.1056/NEJMoa1616288.
11. Gerlinger M, Rowan AJ, Horswell S, Math M, Larkin J, Endesfelder D, et al. Intratumor heterogeneity and branched evolution revealed by multiregion sequencing. N Engl J Med. 2012;366 10:883-92. doi:10.1056/NEJMoa1113205.
12. Hu Z, Ding J, Ma Z, Sun R, Seoane JA, Scott Shaffer J, et al. Quantitative evidence for early metastatic seeding in colorectal cancer. Nat Genet. 2019;51 7:1113-22. doi:10.1038/s41588-019-0423-x.
13. Kim TM, Jung SH, An CH, Lee SH, Baek IP, Kim MS, et al. Subclonal Genomic Architectures of Primary and Metastatic Colorectal Cancer Based on Intratumoral Genetic Heterogeneity. Clin Cancer Res. 2015;21 19:4461-72. doi:10.1158/1078-0432.CCR-14-2413.
14. El-Kebir M, Oesper L, Acheson-Field H and Raphael BJ. Reconstruction of clonal trees and tumor composition from multi-sample sequencing data. Bioinformatics. 2015;31 12:i62-70. doi:10.1093/bioinformatics/btv261.
15. Gerlinger M, Horswell S, Larkin J, Rowan AJ, Salm MP, Varela I, et al. Genomic architecture and evolution of clear cell renal cell carcinomas defined by multiregion sequencing. Nat Genet. 2014;46 3:225-33. doi:10.1038/ng.2891.
16. Liu W, Laitinen S, Khan S, Vihinen M, Kowalski J, Yu G, et al. Copy number analysis indicates monoclonal origin of lethal metastatic prostate cancer. Nat Med. 2009;15 5:559-65. doi:10.1038/nm.1944.
17. Huang Y, Gao S, Wu S, Song P, Sun X, Hu X, et al. Multilayered molecular profiling supported the monoclonal origin of metastatic renal cell carcinoma. Int J Cancer. 2014;135 1:78-87. doi:10.1002/ijc.28654.
18. Cheung KJ, Padmanaban V, Silvestri V, Schipper K, Cohen JD, Fairchild AN, et al. Polyclonal breast cancer metastases arise from collective dissemination of keratin 14-expressing tumor cell clusters. Proc Natl Acad Sci U S A. 2016;113 7:E854-63. doi:10.1073/pnas.1508541113.
19. Beltran H and Demichelis F. Prostate cancer: Intrapatient heterogeneity in prostate cancer. Nat Rev Urol. 2015;12 8:430-1. doi:10.1038/nrurol.2015.182.
20. Wang D, Niu X, Wang Z, Song CL, Huang Z, Chen KN, et al. Multiregion Sequencing Reveals the Genetic Heterogeneity and Evolutionary History of Osteosarcoma and Matched Pulmonary Metastases. Cancer Res. 2019;79 1:7-20. doi:10.1158/0008-5472.CAN-18-1086.
21. Yan T, Cui H, Zhou Y, Yang B, Kong P, Zhang Y, et al. Multi-region sequencing unveils novel actionable targets and spatial heterogeneity in esophageal squamous cell carcinoma. Nat Commun. 2019;10 1:1670. doi:10.1038/s41467-019-09255-1.
22. Mayakonda A, Lin D-C, Assenov Y, Plass C and Koeffler HP. Maftools: efficient and comprehensive analysis of somatic variants in cancer. Genome research. 2018;28 11:1747-56. doi:10.1101/gr.239244.118.
23. Blokzijl F, Janssen R, van Boxtel R and Cuppen E. MutationalPatterns: comprehensive genome-wide analysis of mutational processes. Genome Med. 2018;10 1:33. doi:10.1186/s13073-018-0539-0.

24. Rosenthal R, McGranahan N, Herrero J, Taylor BS and Swanton C. DeconstructSigs: delineating mutational processes in single tumors distinguishes DNA repair deficiencies and patterns of carcinoma evolution. Genome Biol. 2016;17:31. doi:10.1186/s13059-016-0893-4.
25. Shinde J, Bayard Q, Imbeaud S, Hirsch TZ, Liu F, Renault V, et al. Palimpsest: an R package for studying mutational and structural variant signatures along clonal evolution in cancer. Bioinformatics. 2018;34 19:3380-1. doi:10.1093/bioinformatics/bty388.
26. Miller CA, White BS, Dees ND, Griffith M, Welch JS, Griffith OL, et al. SciClone: inferring clonal architecture and tracking the spatial and temporal patterns of tumor evolution. PLoS Comput Biol. 2014;10 8:e1003665. doi:10.1371/journal.pcbi.1003665.
27. Jiao W, Vembu S, Deshwar AG, Stein L and Morris Q. Inferring clonal evolution of tumors from single nucleotide somatic mutations. BMC Bioinformatics. 2014;15:35. doi:10.1186/1471-2105-15-35.
28. Roth A, Khattra J, Yap D, Wan A, Laks E, Biele J, et al. PyClone: statistical inference of clonal population structure in cancer. Nat Methods. 2014;11 4:396-8. doi:10.1038/nmeth.2883.
29. Niknafs N, Beleva-Guthrie V, Naiman DQ and Karchin R. SubClonal Hierarchy Inference from Somatic Mutations: Automatic Reconstruction of Cancer Evolutionary Trees from Multi-region Next Generation Sequencing. PLoS Comput Biol. 2015;11 10:e1004416. doi:10.1371/journal.pcbi.1004416.
30. Nik-Zainal S, Van Loo P, Wedge DC, Alexandrov LB, Greenman CD, Lau KW, et al. The life history of 21 breast cancers. Cell. 2012;149 5:994-1007. doi:10.1016/j.cell.2012.04.023.
31. Deshwar AG, Vembu S, Yung CK, Jang GH, Stein L and Morris Q. PhyloWGS: reconstructing subclonal composition and evolution from whole-genome sequencing of tumors. Genome Biol. 2015;16:35. doi:10.1186/s13059-015-0602-8.
32. Caravagna G, Heide T, Williams MJ, Zapata L, Nichol D, Chkhaidze K, et al. Subclonal reconstruction of tumors by using machine learning and population genetics. Nat Genet. 2020;52 9:898-907. doi:10.1038/s41588-020-0675-5.
33. Cibulskis K, Lawrence MS, Carter SL, Sivachenko A, Jaffe D, Sougnez C, et al. Sensitive detection of somatic point mutations in impure and heterogeneous cancer samples. Nat Biotechnol. 2013;31 3:213-9. doi:10.1038/nbt.2514.
34. Saunders CT, Wong WS, Swamy S, Becq J, Murray LJ and Cheetham RK. Strelka: accurate somatic small-variant calling from sequenced tumor-normal sample pairs. Bioinformatics. 2012;28 14:1811-7. doi:10.1093/bioinformatics/bts271.
35. Stachler MD, Taylor-Weiner A, Peng S, McKenna A, Agoston AT, Odze RD, et al. Paired exome analysis of Barrett's esophagus and adenocarcinoma. Nat Genet. 2015;47 9:1047-55. doi:10.1038/ng.3343.
36. Li H, Handsaker B, Wysoker A, Fennell T, Ruan J, Homer N, et al. The Sequence Alignment/Map format and SAMtools. Bioinformatics. 2009;25 16:2078-9. doi:10.1093/bioinformatics/btp352.
37. Wang K, Li M and Hakonarson H. ANNOVAR: functional annotation of genetic variants from high-throughput sequencing data. Nucleic Acids Res. 2010;38 16:e164. doi:10.1093/nar/gkq603.

38. Favero F, Joshi T, Marquard AM, Birkbak NJ, Krzystanek M, Li Q, et al. Sequenza: allele-specific copy number and mutation profiles from tumor sequencing data. Ann Oncol. 2015;26 1:64-70. doi:10.1093/annonc/mdu479.
39. Williams MJ, Werner B, Barnes CP, Graham TA and Sottoriva A. Identification of neutral tumor evolution across cancer types. Nat Genet. 2016;48 3:238-44. doi:10.1038/ng.3489.
40. Durrett R. Population Genetics of Neutral Mutations in Exponentially Growing Cancer Cell Populations. Ann Appl Probab. 2013;23 1:230-50. doi:10.1214/11-aap824.
41. Zhang C, Zhang L, Xu T, Xue R, Yu L, Zhu Y, et al. Mapping the spreading routes of lymphatic metastases in human colorectal cancer. Nat Commun. 2020;11 1:1993. doi:10.1038/s41467-020-15886-6.
42. Hu Z, Li Z, Ma Z and Curtis C. Multi-cancer analysis of clonality and the timing of systemic spread in paired primary tumors and metastases. Nat Genet. 2020; doi:10.1038/s41588-020-0628-z.
43. McGranahan N, Favero F, de Bruin EC, Birkbak NJ, Szallasi Z and Swanton C. Clonal status of actionable driver events and the timing of mutational processes in cancer evolution. Sci Transl Med. 2015;7 283:283ra54. doi:10.1126/scitranslmed.aaa1408.
44. Sun R, Hu Z, Sottoriva A, Graham TA, Harpak A, Ma Z, et al. Between-region genetic divergence reflects the mode and tempo of tumor evolution. Nat Genet. 2017;49 7:1015-24. doi:10.1038/ng.3891.
45. Caswell-Jin JL, McNamara K, Reiter JG, Sun R, Hu Z, Ma Z, et al. Clonal replacement and heterogeneity in breast tumors treated with neoadjuvant HER2-targeted therapy. Nat Commun. 2019;10 1:657. doi:10.1038/s41467-019-08593-4.
46. Scrucca L, Fop M, Murphy TB and Raftery AE. mclust 5: Clustering, Classification and Density Estimation Using Gaussian Finite Mixture Models. R J. 2016;8 1:289-317.
47. Mroz EA, Tward AD, Pickering CR, Myers JN, Ferris RL and Rocco JW. High intratumor genetic heterogeneity is related to worse outcome in patients with head and neck squamous cell carcinoma. Cancer-Am Cancer Soc. 2013;119 16:3034-42. doi:10.1002/cncr.28150.
48. Charoentong P, Finotello F, Angelova M, Mayer C, Efremova M, Rieder D, et al. Pan-cancer Immunogenomic Analyses Reveal Genotype-Immunophenotype Relationships and Predictors of Response to Checkpoint Blockade. Cell reports. 2017;18 1:248-62. doi:10.1016/j.celrep.2016.12.019.
49. Bhatia G, Patterson N, Sankararaman S and Price AL. Estimating and interpreting FST: the impact of rare variants. Genome Res. 2013;23 9:1514-21. doi:10.1101/gr.154831.113.
50. Lee JK, Wang J, Sa JK, Ladewig E, Lee HO, Lee IH, et al. Spatiotemporal genomic architecture informs precision oncology in glioblastoma. Nat Genet. 2017;49 4:594-9. doi:10.1038/ng.3806.
51. Paradis E, Claude J and Strimmer K. APE: Analyses of Phylogenetics and Evolution in R language. Bioinformatics. 2004;20 2:289-90. doi:10.1093/bioinformatics/btg412.
52. Schliep KP. phangorn: phylogenetic analysis in R. Bioinformatics. 2011;27 4:592-3. doi:10.1093/bioinformatics/btq706.

53. Saitou N and Nei M. The neighbor-joining method: a new method for reconstructing phylogenetic trees. Mol Biol Evol. 1987;4 4:406-25. doi:10.1093/oxfordjournals.molbev.a040454.
54. Desper R and Gascuel O. Fast and accurate phylogeny reconstruction algorithms based on the minimum-evolution principle. J Comput Biol. 2002;9 5:687-705. doi:10.1089/106652702761034136.
55. Yang Z. Phylogenetic analysis using parsimony and likelihood methods. J Mol Evol. 1996;42 2:294-307.
56. Felsenstein J. Evolutionary trees from DNA sequences: a maximum likelihood approach. J Mol Evol. 1981;17 6:368-76. doi:10.1007/bf01734359.
57. Youssoufian H, Kazazian HH, Jr., Phillips DG, Aronis S, Tsiftis G, Brown VA, et al. Recurrent mutations in haemophilia A give evidence for CpG mutation hotspots. Nature. 1986;324 6095:380-2. doi:10.1038/324380a0.
58. Hu Z, Sun R and Curtis C. A population genetics perspective on the determinants of intra-tumor heterogeneity. Biochim Biophys Acta Rev Cancer. 2017;1867 2:109-26. doi:10.1016/j.bbcan.2017.03.001.
59. Liu M, Liu Y, Di J, Su Z, Yang H, Jiang B, et al. Multi-region and single-cell sequencing reveal variable genomic heterogeneity in rectal cancer. BMC Cancer. 2017;17 1:787. doi:10.1186/s12885-017-3777-4.
60. Brannon AR, Vakiani E, Sylvester BE, Scott SN, McDermott G, Shah RH, et al. Comparative sequencing analysis reveals high genomic concordance between matched primary and metastatic colorectal cancer lesions. Genome Biol. 2014;15 8:454. doi:10.1186/s13059-014-0454-7.
61. Tan IB, Malik S, Ramnarayanan K, McPherson JR, Ho DL, Suzuki Y, et al. High-depth sequencing of over 750 genes supports linear progression of primary tumors and metastases in most patients with liver-limited metastatic colorectal cancer. Genome Biol. 2015;16:32. doi:10.1186/s13059-015-0589-1.
62. Wang K, Lim HY, Shi S, Lee J, Deng S, Xie T, et al. Genomic landscape of copy number aberrations enables the identification of oncogenic drivers in hepatocellular carcinoma. Hepatology. 2013;58 2:706-17. doi:10.1002/hep.26402.
63. Guichard C, Amaddeo G, Imbeaud S, Ladeiro Y, Pelletier L, Maad IB, et al. Integrated analysis of somatic mutations and focal copy-number changes identifies key genes and pathways in hepatocellular carcinoma. Nat Genet. 2012;44 6:694-8. doi:10.1038/ng.2256.
64. Dagogo-Jack I and Shaw AT. Tumour heterogeneity and resistance to cancer therapies. Nat Rev Clin Oncol. 2018;15 2:81-94. doi:10.1038/nrclinonc.2017.166.
65. Williams MJ, Werner B, Heide T, Curtis C, Barnes CP, Sottoriva A, et al. Quantification of subclonal selection in cancer from bulk sequencing data. Nat Genet. 2018;50 6:895-903. doi:10.1038/s41588-018-0128-6.
66. Rajput A, Bocklage T, Greenbaum A, Lee JH and Ness SA. Mutant-Allele Tumor Heterogeneity Scores Correlate With Risk of Metastases in Colon Cancer. Clin Colorectal Cancer. 2017;16 3:e165-e70. doi:10.1016/j.clcc.2016.11.004.

67. Mroz EA and Rocco JW. MATH, a novel measure of intratumor genetic heterogeneity, is high in poor-outcome classes of head and neck squamous cell carcinoma. Oral Oncol. 2013;49 3:211-5. doi:10.1016/j.oraloncology.2012.09.007.
68. Campbell PJ, Yachida S, Mudie LJ, Stephens PJ, Pleasance ED, Stebbings LA, et al. The patterns and dynamics of genomic instability in metastatic pancreatic cancer. Nature. 2010;467 7319:1109-13. doi:10.1038/nature09460.
69. Ding L, Ley TJ, Larson DE, Miller CA, Koboldt DC, Welch JS, et al. Clonal evolution in relapsed acute myeloid leukaemia revealed by whole-genome sequencing. Nature. 2012;481 7382:506-10. doi:10.1038/nature10738.
70. Griffith M, Miller CA, Griffith OL, Krysiak K, Skidmore ZL, Ramu A, et al. Optimizing cancer genome sequencing and analysis. Cell Syst. 2015;1 3:210-23. doi:10.1016/j.cels.2015.08.015.
71. Xue R, Chen L, Zhang C, Fujita M, Li R, Yan SM, et al. Genomic and Transcriptomic Profiling of Combined Hepatocellular and Intrahepatic Cholangiocarcinoma Reveals Distinct Molecular Subtypes. Cancer Cell. 2019;35 6:932-47 e8. doi:10.1016/j.ccell.2019.04.007.
72. Kuhner MK and Felsenstein J. A simulation comparison of phylogeny algorithms under equal and unequal evolutionary rates. Mol Biol Evol. 1994;11 3:459-68. doi:10.1093/oxfordjournals.molbev.a040126.
73. Alexandrov LB, Nik-Zainal S, Wedge DC, Aparicio SA, Behjati S, Biankin AV, et al. Signatures of mutational processes in human cancer. Nature. 2013;500 7463:415-21. doi:10.1038/nature12477.
74. Huang X, Wojtowicz D and Przytycka TM. Detecting presence of mutational signatures in cancer with confidence. Bioinformatics. 2018;34 2:330-7. doi:10.1093/bioinformatics/btx604.
75. Mengni Liu (2021) Supporting code and data for "MesKit: a tool kit for dissecting cancer evolution of multi-region tumor biopsies through somatic alterations" [Source Code]. https://doi.org/10.24433/CO.6811520.v2
76. Liu M, Chen J, Wang X, Wang C, Zhang X, Xie Y et al. Supporting data for "MesKit: a tool kit for dissecting cancer evolution of multi-region tumor biopsies through somatic alterations" GigaScience Database. 2021 http://dx.doi.org/10.5524/100891

Figure 1

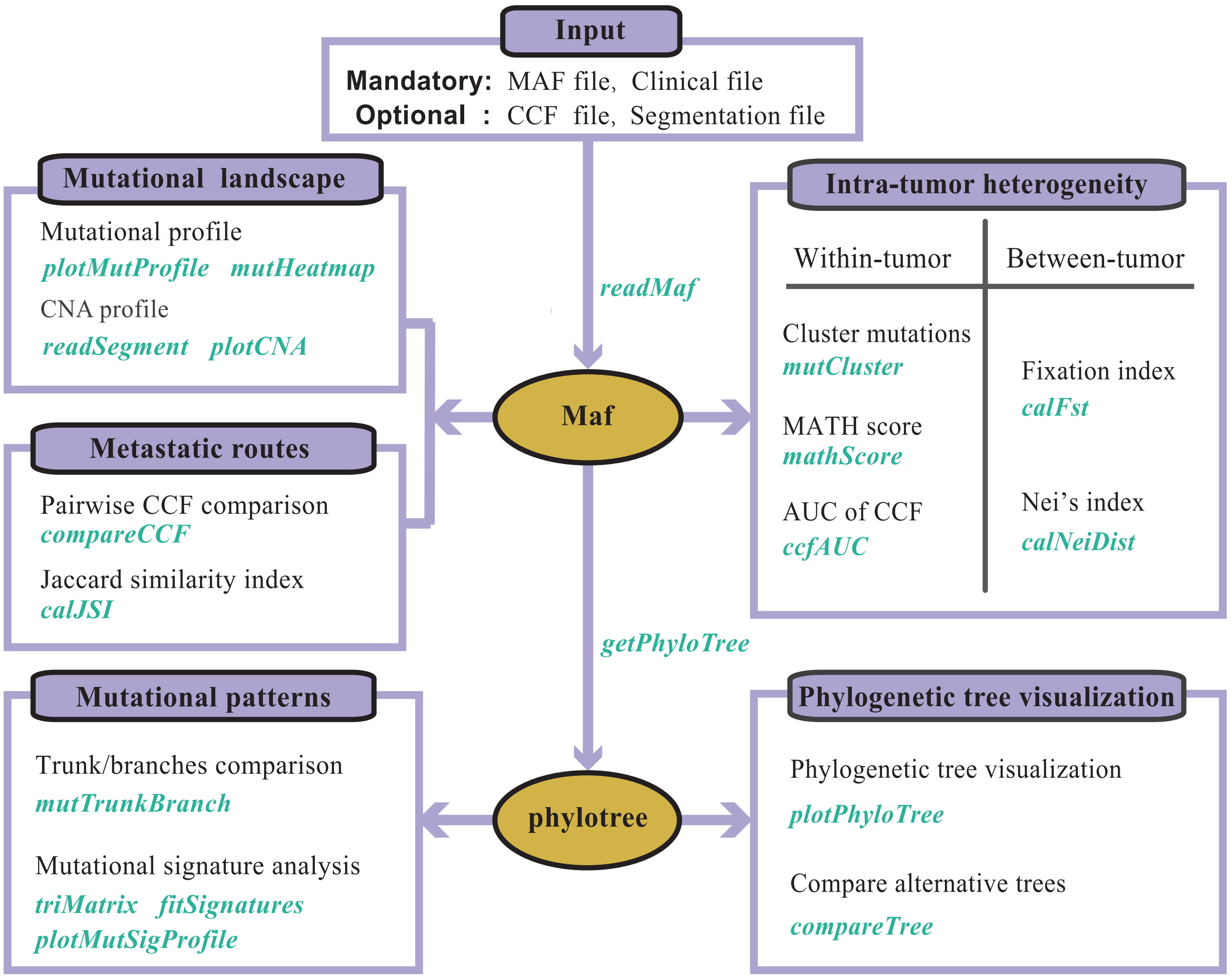
Input
Mandatory: MAF file, Clinical file
Optional : CCF file, Segmentation file
readMaf
Maf
getPhyloTree
phylotree
Mutational landscape
Mutational profile
plotMutProfile mutHeatmap
CNA profile
readSegment plotCNA
Metastatic routes
Pairwise CCF comparison
compareCCF
Jaccard similarity index
calJSI
Intra-tumor heterogeneity
Within-tumor
Between-tumor
Cluster mutations
mutCluster
MATH score
mathScore
AUC of CCF
ccfAUC
Fixation index
calFst
Nei's index
calNeiDist
Mutational patterns
Trunk/branches comparison
mutTrunkBranch
Mutational signature analysis
triMatrix fitSignatures
plotMutSigProfile
Phylogenetic tree visualization
Phylogenetic tree visualization
plotPhyloTree
Compare alternative trees
compareTree

Figure 2 Click here to access/download;Figure;Figure2.pdf

Figure 2

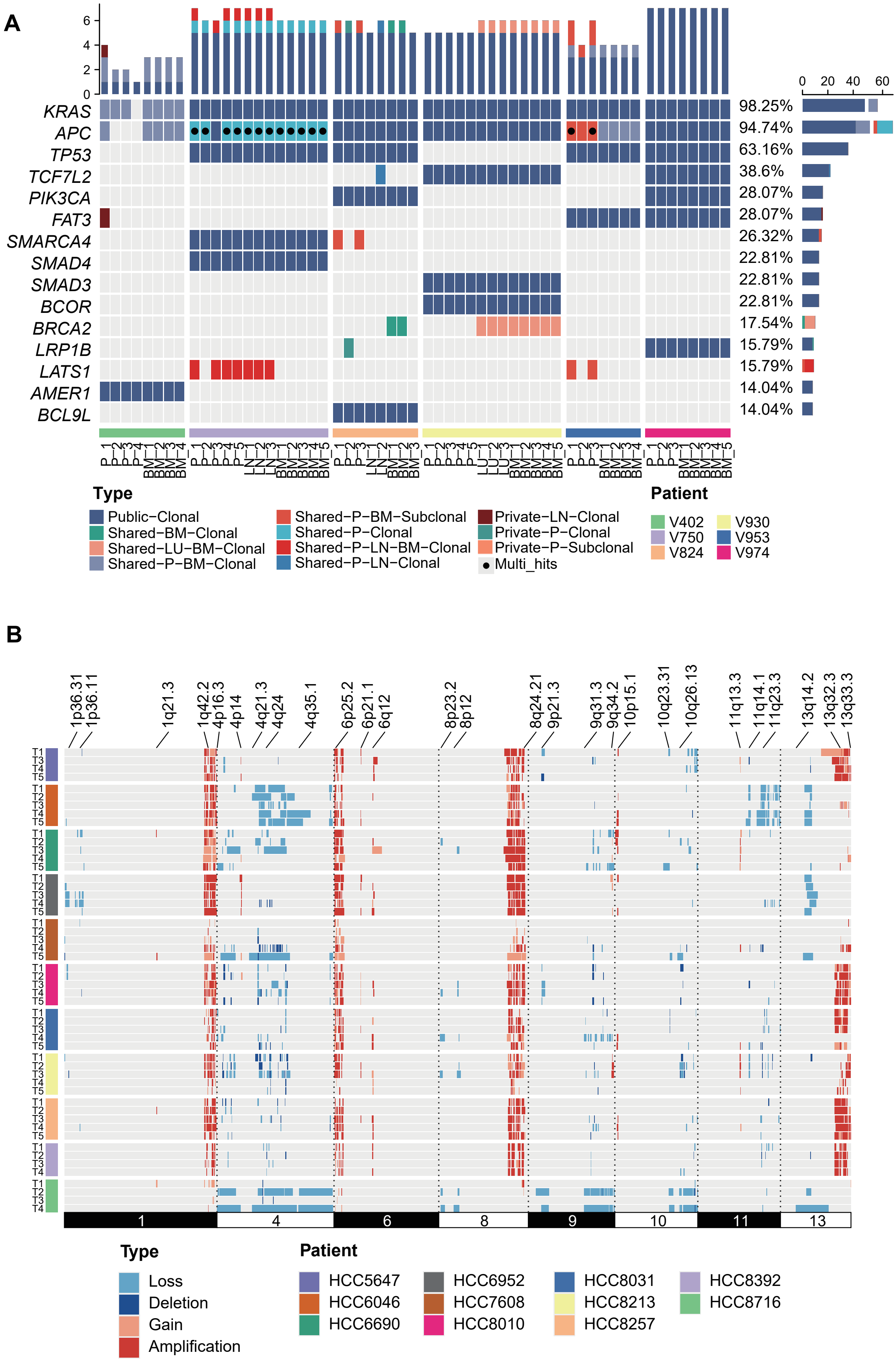

Figure 3

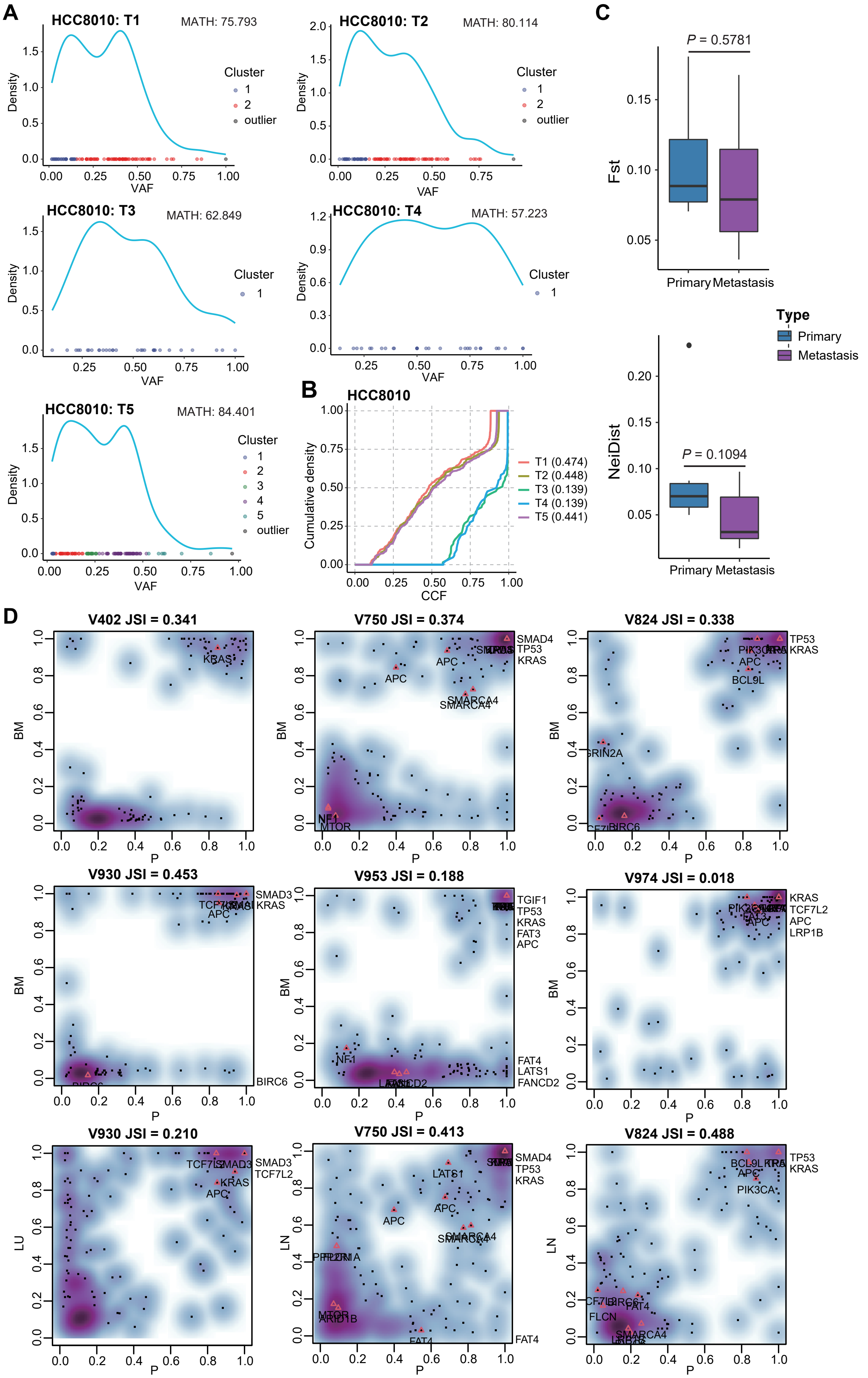
A
HCC8010: T1
MATH: 75.793
HCC8010: T2
MATH: 80.114
HCC8010: T3
MATH: 62.849
HCC8010: T4
MATH: 57.223
HCC8010: T5
MATH: 84.401
Cluster
outlier
Density
VAF
B
HCC8010
Cumulative density
CCF
T1 (0.474)
T2 (0.448)
T3 (0.139)
T4 (0.139)
T5 (0.441)
C
P = 0.5781
Fst
P = 0.1094
NeiDist
Primary
Metastasis
Type
D
V402 JSI = 0.341
V750 JSI = 0.374
V824 JSI = 0.338
V930 JSI = 0.453
V953 JSI = 0.188
V974 JSI = 0.018
V930 JSI = 0.210
V750 JSI = 0.413
V824 JSI = 0.488
BM
LU
LN
P

Figure 4 Click here to access/download;Figure;Figure4.pdf

Figure 4

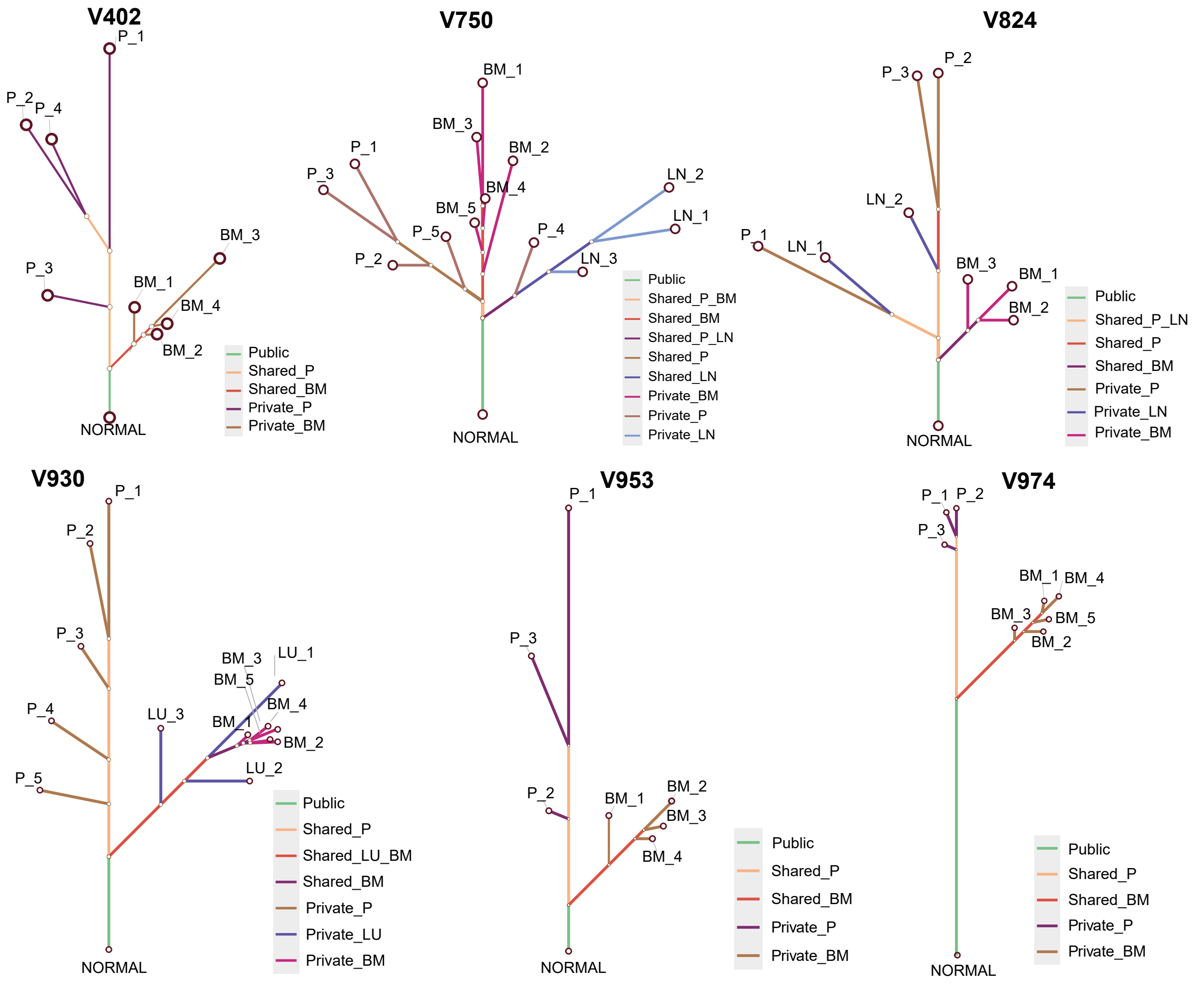

Figure 5 Click here to access/download;Figure;Figure5.pdf

Figure 5

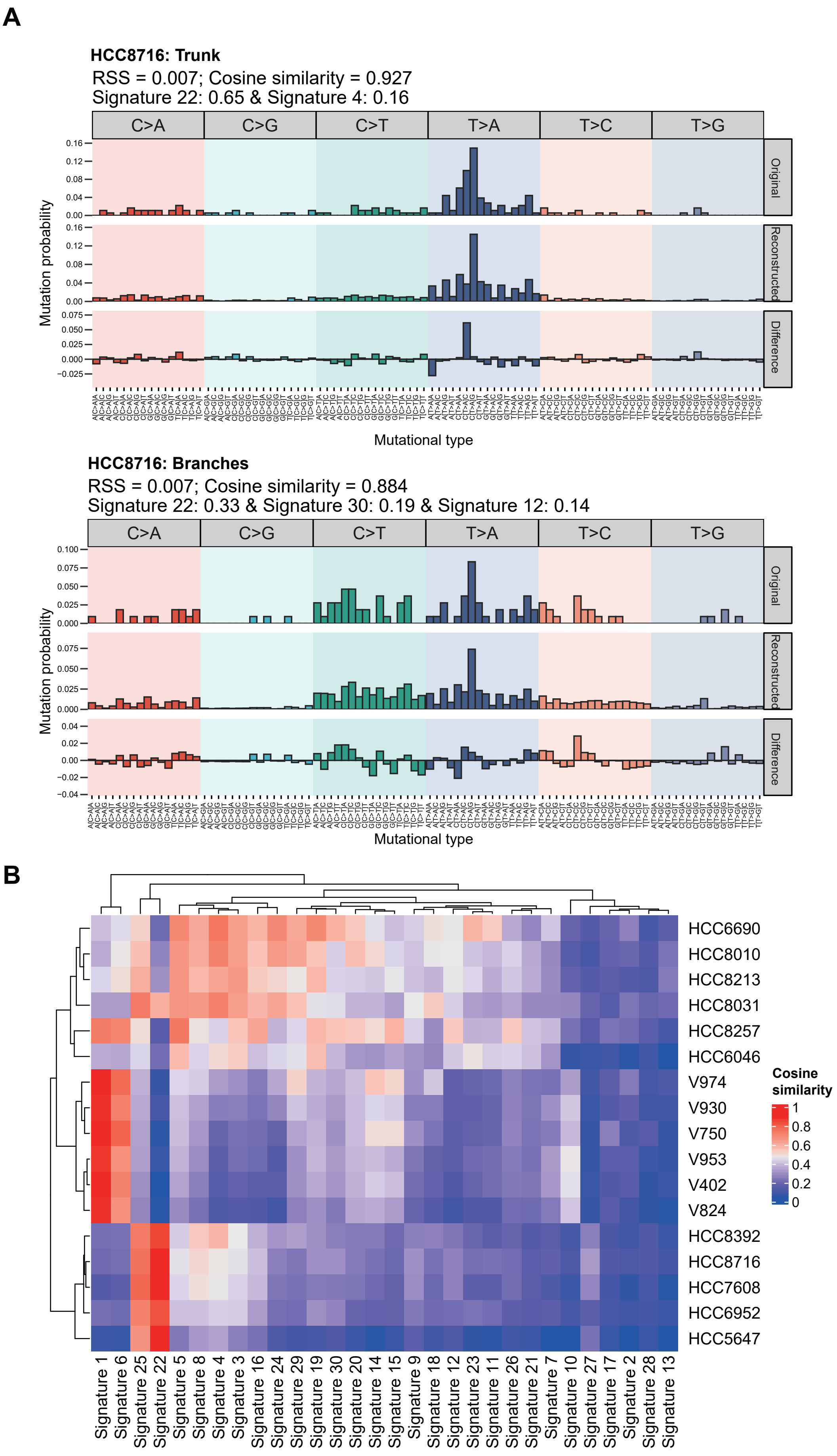

Supplementary method and figures

Click here to access/download
Supplementary Material
Supplementary Method and Figures.pdf

Supplementary Table S1

Click here to access/download
Supplementary Material
Supplementary Table S1.docx

Supplementary Table S2

Click here to access/download
Supplementary Material
Supplementary Table S2.xlsx

Supplementary Table S3

Click here to access/download
Supplementary Material
Supplementary Table S3.xlsx

Supplementary Table S4

Click here to access/download
Supplementary Material
Supplementary Table S4.xlsx
