# Supplementary material for: MesKit: a tool kit for dissecting cancer evolution of multi-region tumor biopsies through somatic alterations
Source: Gigascience. 2021 May 21;10(5):giab036. doi: 10.1093/gigascience/giab036 (PMC8138830; doi:10.1093/gigascience/giab036)

Supplementary material to MesKit: a tool kit for dissecting cancer evolution of multi-region tumor biopsies through somatic alterations

Supplementary Method

Phylogenetic visualization auto-layout algorithm

MesKit implemented an auto-layout algorithm for visualizing phylogenetic tree based on somatic mutations. For a phylogenetic tree of an individual patient, we artificially set a non-mutated, normal sample as an outgroup root (Figure S6). The adjacent node of the root is placed on the origin of the rectangular coordinate system, which is selected as the starting node. For each subtree T_s with starting node s, N_i denotes the number of descendants of node i, where $N_i = 1$ if i is a leaf node; ω_i denotes the wedge size occupied by the node i. Tree edges are directed away from the root, for branch $\delta(i,j)$, node i is the parent of node j. Let α_j denote the included angle between branch $\delta(i,j)$ and x axis. Ordinal indicators of the node set are written using superscript, for example, $B^{[x]}$ refers to the xth node of node set B. The branch length can be extracted from the phyloTree object, which is proportional to the numbers of somatic mutations. The algorithm comprises five steps explained below:

1. We identify the longest branch of tree T_s and assign it as the angular bisector of ω_s. $\sum N_L^i$ and $\sum N_R^i$ represent the total number of child nodes of node i in the left wedges and right wedges, respectively. In Figure S4, $\sum N_R^0 = N_1 + N_7$.
2. Then, we create a node set K, which contains nodes situated on the longest branch of T_s. Let α_K denote the angle between the longest branch of T_s and x axis. For each node $j \in K$, $\alpha_j = \alpha_K$. The adjacent nodes of set K are stored in a node set B. To balance the distribution of nodes on both sides of T_s, we traverse the set B: when $\alpha_K \geq \frac{\pi}{2}$, the first node $B^{[1]}$ will be placed on the right, otherwise on the left. For any node $B^{[x]}$ $(x > 1)$, if $\sum N_R^i > \sum N_L^i$, it will be placed on the left, vice versa.
3. To avoid overlap between subtrees, we set the angular space of each node to be proportional to the number of its descendants. And $\omega_{L^{[x]}}$ and $\omega_{R^{[x]}}$ are calculated as below:

$$\begin{cases} \omega_{L[x]} = \frac{\omega_r}{2} \times \frac{N_{L[x]}}{\sum N_L} \\ \omega_{R[x]} = \frac{\omega_r}{2} \times \frac{N_{R[x]}}{\sum N_R} \end{cases} \quad (1)$$

In Figure S4, $L^{[1]}$ is node 3, ω_r is ω_0 :

$$\omega_3 = \frac{\omega_0}{2} \times \frac{N_3}{N_{3+N_6}} \quad (2)$$

Then, we place each branch on the angle bisector of $\omega_{L[x]}$ or $\omega_{R[x]}$.

For $L^{[x]}$ on the left of tree:

$$\begin{cases} \alpha_{L[1]} = \frac{\omega_{L[1]}}{2} + \alpha_K - \frac{\pi}{2} & (x = 1) \\ \alpha_{L[x]} = \alpha_{L[x-1]} + \frac{\omega_{L[x-1]}}{2} + \frac{\omega_{L[x]}}{2} & (x > 1) \end{cases} \quad (3)$$

For $R^{[x]}$ on the right of tree:

$$\begin{cases} \alpha_{R[1]} = \frac{\omega_{R[1]}}{2} + \alpha_K - \frac{\pi}{2} & (x = 1) \\ \alpha_{R[x]} = \alpha_{R[x-1]} + \frac{\omega_{R[x-1]}}{2} + \frac{\omega_{R[x]}}{2} & (x > 1) \end{cases} \quad (4)$$

In Figure S4, $R^{[1]}$ is node 1, $R^{[2]}$ is node 7:

$$\alpha_1 = \frac{\omega_1}{2} + \alpha_K - \frac{\pi}{2} \quad (5)$$

$$\alpha_7 = \alpha_1 + \frac{\omega_1}{2} + \frac{\omega_7}{2} \quad (6)$$

4. For branch $\delta(i, j)$, the coordinate of node j is calculated as:

$$\begin{cases} X_j = \cos \alpha_1 \times length(\delta(i, j)) + X_i \\ Y_j = \sin \alpha_1 \times length(\delta(i, j)) + Y_i \end{cases} \quad (7)$$

the coordinate of node 1 is:

$$\begin{cases} X_1 = \cos \alpha_1 \times length(\delta(0,1)) + X_0 \\ Y_1 = \sin \alpha_1 \times length(\delta(0,1)) + Y_0 \end{cases} \quad (8)$$

5. Stop the loop and assign the root node right below node 0 when all internal nodes have been traversed.

Supplementary Figures

Figure S1. Mutational landscape of HCC and CRC cohorts

A. Mutational profile of HCC cohort. Oncoprint of top 15 most frequently mutated driver genes of HCC were grouped by public, shared or private mutations including both clonal and subclonal drivers. Stacked bar charts on the top and right show the number of mutations for different types per sample and per driver gene, respectively. Genes were sorted by mutational frequency and samples were split by patients as indicated by the annotation bar (bottom). **B**. The consistent CNAs of CRC cohort with significant recurring CNAs identified from TCGA Colorectal Adenocarcinoma project by GISTIC2.0 (obtained from Broad GDAC website). Each track represents one tumor sample. P, primary tumor; BM, brain metastasis; LN, lymph node metastasis; LU, lung metastasis. Dark red for amplifications ($CN \geq 4$), light red for gains ($2 < CN < 4$), dark blue for deletions ($CN = 0$), and light blue for losses ($0 < CN < 2$).

Figure S1

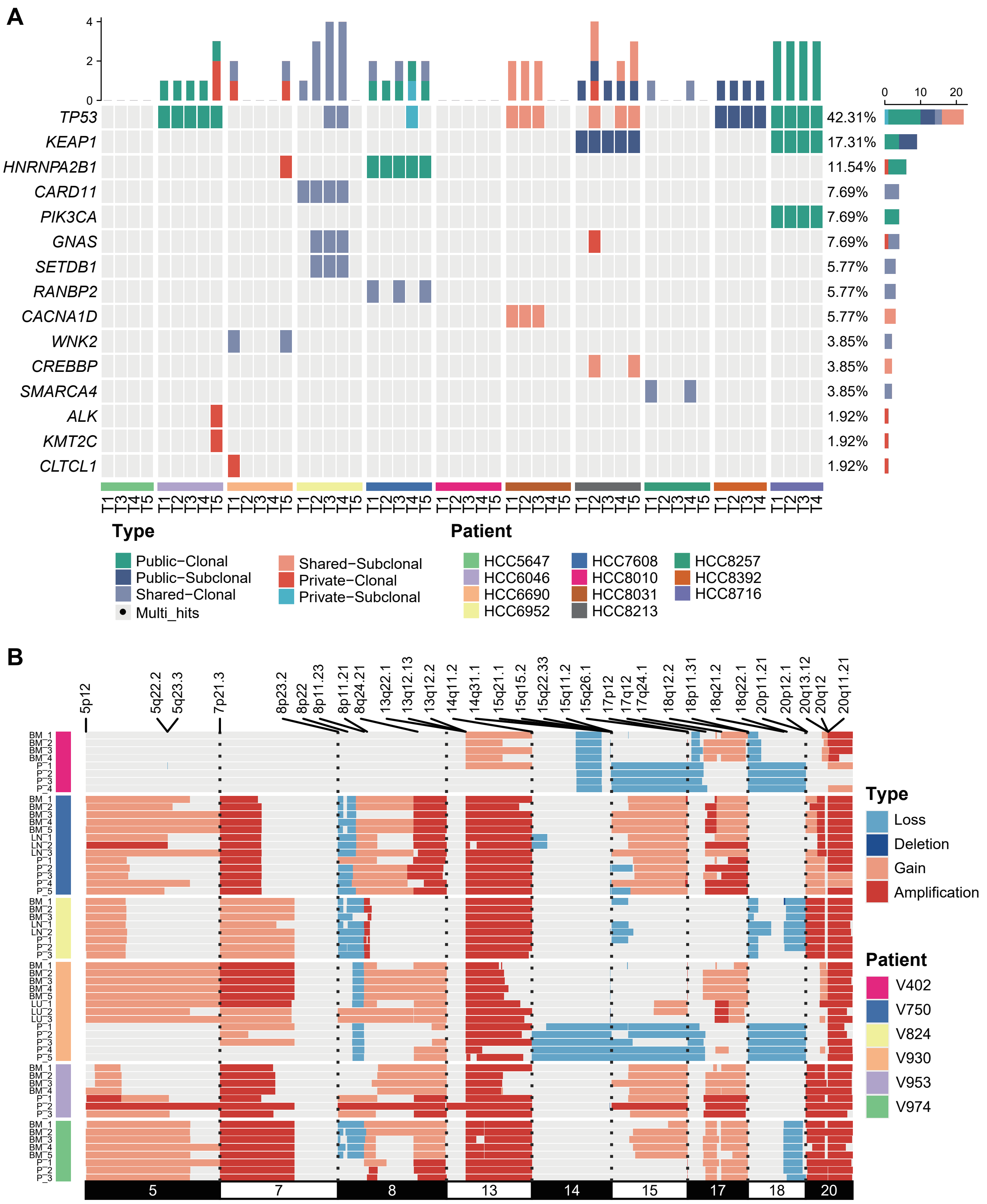
A
TP53
KEAP1
HNRNPA2B1
CARD11
PIK3CA
GNAS
SETDB1
RANBP2
CACNA1D
WNK2
CREBBP
SMARCA4
ALK
KMT2C
CLTCL1
42.31%
17.31%
11.54%
7.69%
7.69%
7.69%
5.77%
5.77%
5.77%
3.85%
3.85%
3.85%
1.92%
1.92%
1.92%
Type
Public−Clonal
Public−Subclonal
Shared−Clonal
Multi_hits
Shared−Subclonal
Private−Clonal
Private−Subclonal
Patient
HCC5647
HCC6046
HCC6690
HCC6952
HCC7608
HCC8010
HCC8031
HCC8213
HCC8257
HCC8392
HCC8716
B
5p12
5q22.2
5q23.3
7p21.3
8p23.2
8p22
8p11.23
8p11.21
8q24.21
13q22.1
13q12.13
13q12.2
14q11.2
14q31.1
15q21.1
15q15.2
15q22.33
15q11.2
15q26.1
17p12
17q12
17q24.1
18q12.2
18p11.31
18q21.2
18q22.1
20p11.21
20p12.1
20q13.12
20q12
20q11.21
Type
Loss
Deletion
Gain
Amplification
Patient
V402
V750
V824
V930
V953
V974
5
7
8
13
14
15
17
18
20

Figure 2. CCF heat maps of CRC cohort

The heat maps of CCF values of tumor samples from the same patient. The color bar next to the heatmap indicates the classification of mutations shared amongst different samples. The proportion of each classification is indicated in the legend. Putative CRC driver genes were labelled on the right.

Figure S2

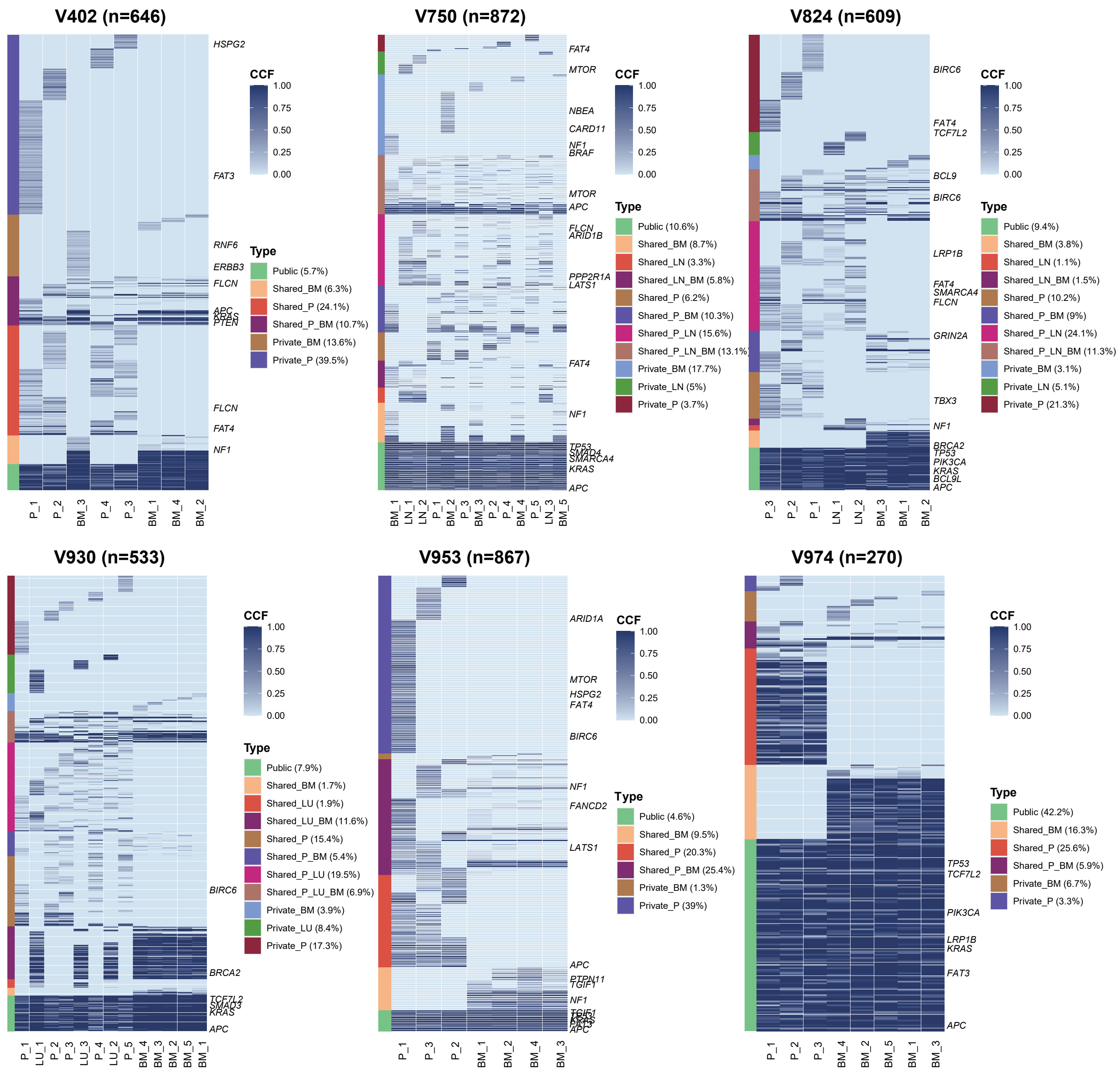

V402 (n=646)
HSPG2
FAT3
RNF6
ERBB3
FLCN
APC
KRAS
PTEN
FLCN
FAT4
NF1
P_1
P_2
BM_3
P_4
P_3
BM_1
BM_4
BM_2
CCF
1.00
0.75
0.50
0.25
0.00
Type
Public (5.7%)
Shared_BM (6.3%)
Shared_P (24.1%)
Shared_P_BM (10.7%)
Private_BM (13.6%)
Private_P (39.5%)
V750 (n=872)
FAT4
MTOR
NBEA
CARD11
NF1
BRAF
MTOR
APC
FLCN
ARID1B
PPP2R1A
LATS1
FAT4
NF1
TP53
SMAD4
SMARCA4
KRAS
APC
BM_1
LN_1
LN_2
P_1
BM_2
BM_3
P_3
P_2
P_4
BM_4
P_5
LN_3
BM_5
CCF
1.00
0.75
0.50
0.25
0.00
Type
Public (10.6%)
Shared_BM (8.7%)
Shared_LN (3.3%)
Shared_LN_BM (5.8%)
Shared_P (6.2%)
Shared_P_BM (10.3%)
Shared_P_LN (15.6%)
Shared_P_LN_BM (13.1%)
Private_BM (17.7%)
Private_LN (5%)
Private_P (3.7%)
V824 (n=609)
BIRC6
FAT4
TCF7L2
BCL9
BIRC6
LRP1B
FAT4
SMARCA4
FLCN
GRIN2A
TBX3
NF1
BRCA2
TP53
PIK3CA
KRAS
BCL9L
APC
P_3
P_2
P_1
LN_1
LN_2
BM_3
BM_1
BM_2
CCF
1.00
0.75
0.50
0.25
0.00
Type
Public (9.4%)
Shared_BM (3.8%)
Shared_LN (1.1%)
Shared_LN_BM (1.5%)
Shared_P (10.2%)
Shared_P_BM (9%)
Shared_P_LN (24.1%)
Shared_P_LN_BM (11.3%)
Private_BM (3.1%)
Private_LN (5.1%)
Private_P (21.3%)
V930 (n=533)
BIRC6
BRCA2
TCF7L2
SMAD3
KRAS
APC
P_1
LU_1
P_2
P_3
LU_3
P_4
LU_2
P_5
BM_4
BM_3
BM_2
BM_5
BM_1
CCF
1.00
0.75
0.50
0.25
0.00
Type
Public (7.9%)
Shared_BM (1.7%)
Shared_LU (1.9%)
Shared_LU_BM (11.6%)
Shared_P (15.4%)
Shared_P_BM (5.4%)
Shared_P_LU (19.5%)
Shared_P_LU_BM (6.9%)
Private_BM (3.9%)
Private_LU (8.4%)
Private_P (17.3%)
V953 (n=867)
ARID1A
MTOR
HSPG2
FAT4
BIRC6
NF1
FANCD2
LATS1
APC
PTPN11
TGIF1
NF1
APC
P_1
P_3
P_2
BM_1
BM_2
BM_4
BM_3
CCF
1.00
0.75
0.50
0.25
0.00
Type
Public (4.6%)
Shared_BM (9.5%)
Shared_P (20.3%)
Shared_P_BM (25.4%)
Private_BM (1.3%)
Private_P (39%)
V974 (n=270)
TP53
TCF7L2
PIK3CA
LRP1B
KRAS
FAT3
APC
P_1
P_2
P_3
BM_4
BM_2
BM_5
BM_1
BM_3
CCF
1.00
0.75
0.50
0.25
0.00
Type
Public (42.2%)
Shared_BM (16.3%)
Shared_P (25.6%)
Shared_P_BM (5.9%)
Private_BM (6.7%)
Private_P (3.3%)

Figure S3. Comparison of phylogenetic trees constructed by different methods of the CRC cohort

Comparison of the MP-based phylogenetic trees against those constructed by NJ method and ML method for each CRC patient. For each pair, the different clades between two phylogenetic trees were highlight in red (the first tree) or blue (the second tree).

Figure S3

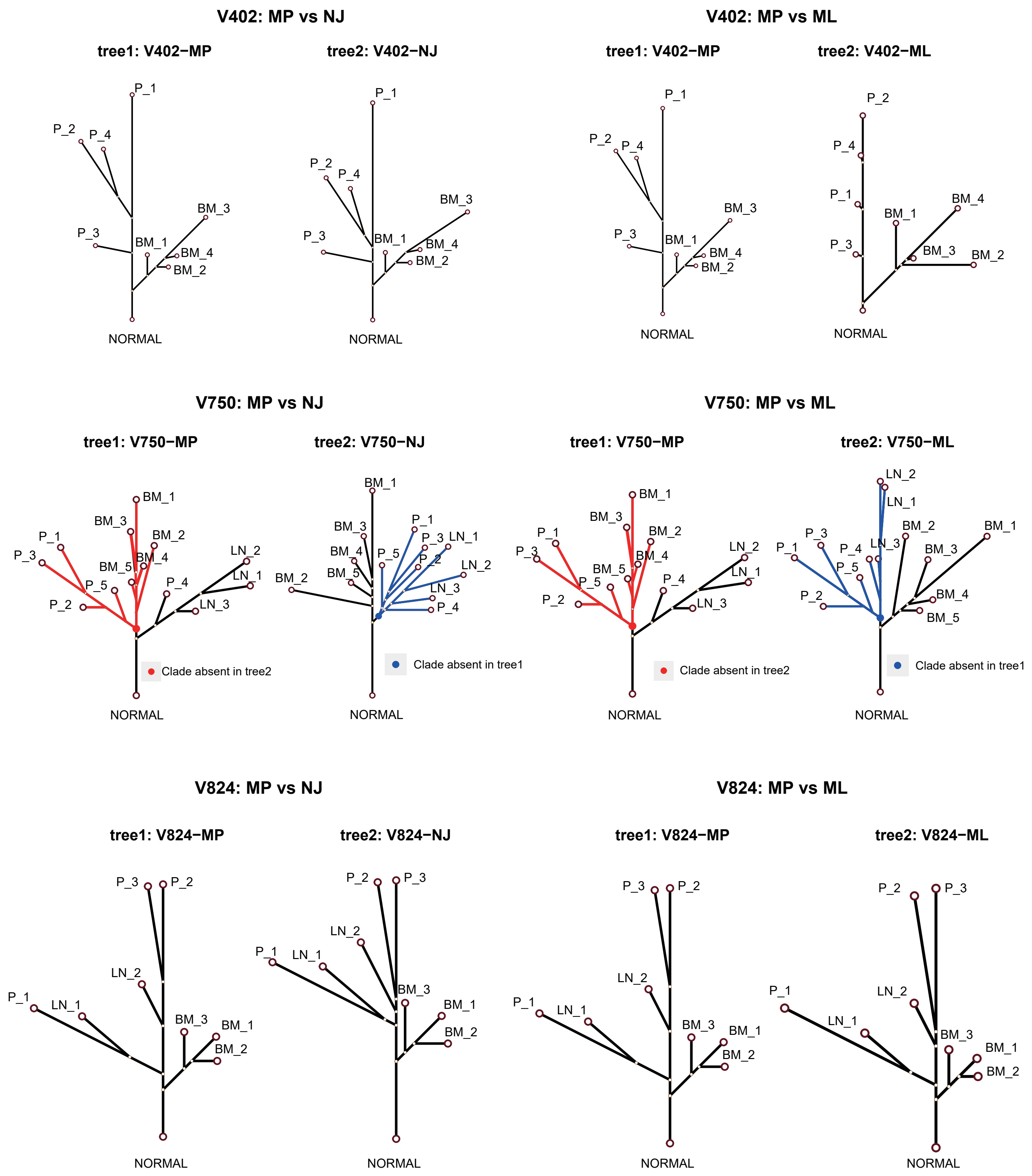
V402: MP vs NJ
tree1: V402−MP
P_1
P_2
P_4
BM_3
P_3
BM_1
BM_4
BM_2
NORMAL
tree2: V402−NJ
P_1
P_2
P_4
BM_3
P_3
BM_1
BM_4
BM_2
NORMAL
V402: MP vs ML
tree1: V402−MP
P_1
P_2
P_4
BM_3
P_3
BM_1
BM_4
BM_2
NORMAL
tree2: V402−ML
P_2
P_4
P_1
BM_4
BM_1
P_3
BM_3
BM_2
NORMAL
V750: MP vs NJ
tree1: V750−MP
BM_1
BM_3
BM_2
P_1
P_3
BM_4
BM_5
LN_2
LN_1
P_5
P_4
P_2
LN_3
Clade absent in tree2
NORMAL
tree2: V750−NJ
BM_1
BM_3
P_1
P_3
LN_1
BM_4
P_5
P_2
LN_2
BM_2
BM_5
LN_3
P_4
Clade absent in tree1
NORMAL
V750: MP vs ML
tree1: V750−MP
BM_1
BM_3
BM_2
P_1
P_3
BM_4
BM_5
LN_2
LN_1
P_5
P_4
P_2
LN_3
Clade absent in tree2
NORMAL
tree2: V750−ML
LN_2
LN_1
P_3
BM_2
BM_1
P_1
P_4
LN_3
BM_3
P_5
P_2
BM_4
BM_5
Clade absent in tree1
NORMAL
V824: MP vs NJ
tree1: V824−MP
P_3
P_2
LN_2
P_1
LN_1
BM_3
BM_1
BM_2
NORMAL
tree2: V824−NJ
P_2
P_3
LN_2
P_1
LN_1
BM_3
BM_1
BM_2
NORMAL
V824: MP vs ML
tree1: V824−MP
P_3
P_2
LN_2
P_1
LN_1
BM_3
BM_1
BM_2
NORMAL
tree2: V824−ML
P_2
P_3
P_1
LN_2
LN_1
BM_3
BM_1
BM_2
NORMAL

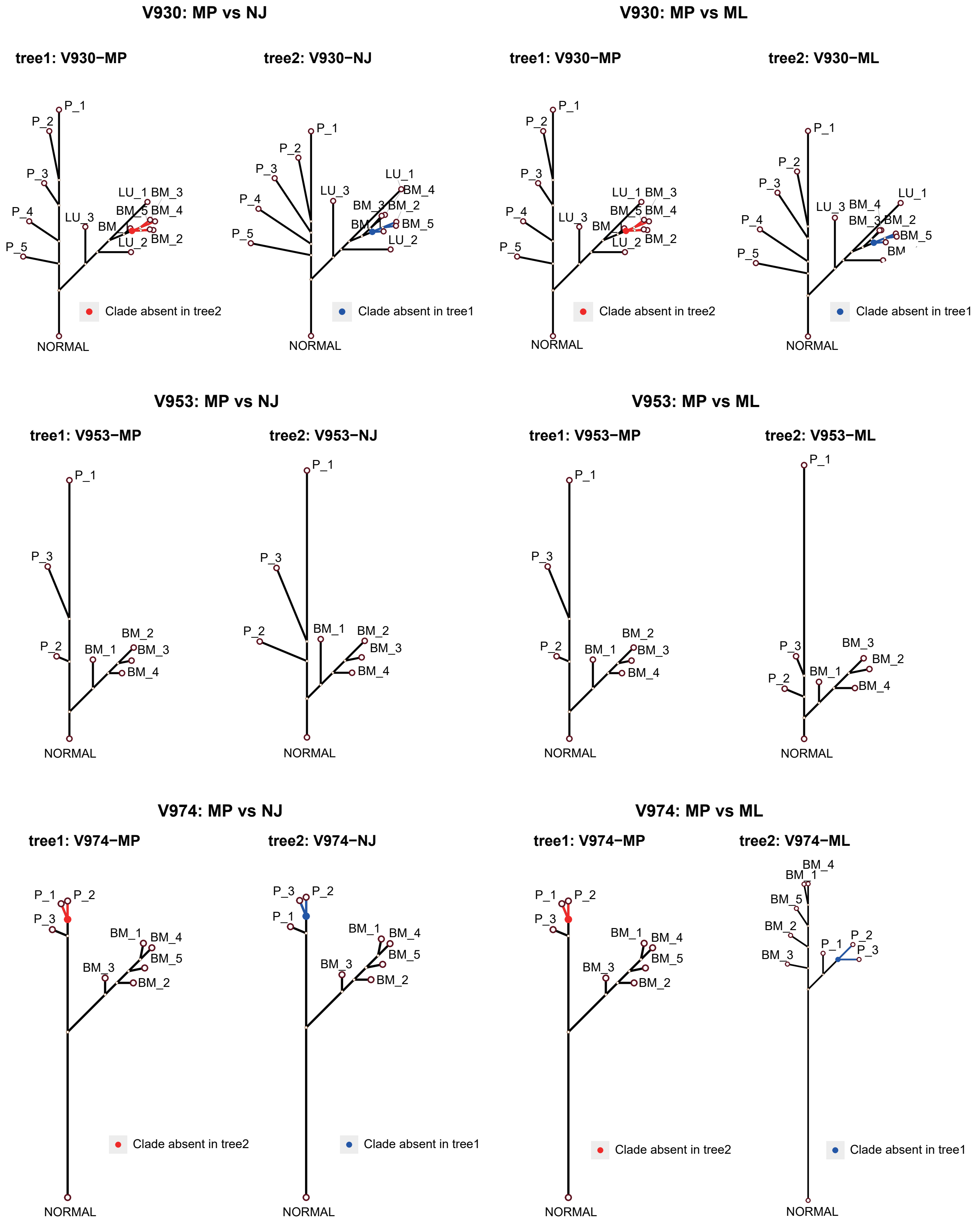

Figure S3 - continued

Figure S4. Comparison of signature contributions measured by MesKit, MutationalPatterns, SignatureEstimation and deconstructSigs

A. Relative contributions of all 30 COSMIC signatures for each patient in the HCC and CRC cohorts.

B. Cosine similarity and RSS between the original and the reconstructed mutational profiles.

Figure S4

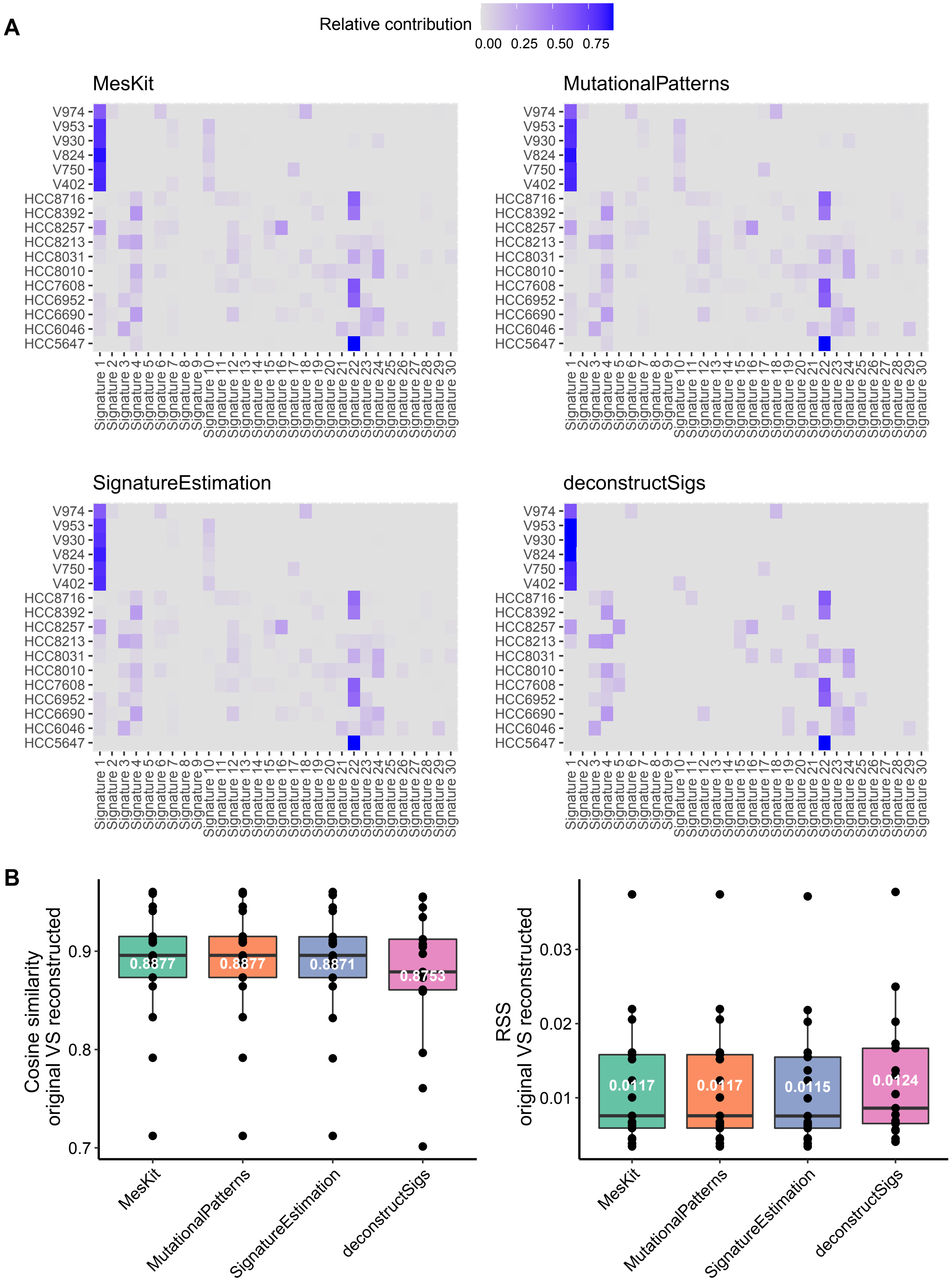
A
Relative contribution
0.00 0.25 0.50 0.75
MesKit
MutationalPatterns
SignatureEstimation
deconstructSigs
V974
V953
V930
V824
V750
V402
HCC8716
HCC8392
HCC8257
HCC8213
HCC8031
HCC8010
HCC7608
HCC6952
HCC6690
HCC6046
HCC5647
Signature 1 … Signature 30
B
Cosine similarity original VS reconstructed
0.7
0.8
0.9
0.8877
0.8877
0.8871
0.8753
RSS original VS reconstructed
0.01
0.02
0.03
0.0117
0.0117
0.0115
0.0124
MesKit
MutationalPatterns
SignatureEstimation
deconstructSigs

Figure S5. Mutation spectra of truncal and branch mutations of HCC5647, HCC7608 and HCC8716

Stacked bar plots show the proportions of truncal and branch mutations accounted for by each of the six mutation types in HCC5647, HCC7608 and HCC8716. The number of analyzed mutations is displayed on top of each bar. A Fisher exact test was used to compare truncal and branch mutations for each mutation type (two-sided: *$P < 0.01$).

Figure S5

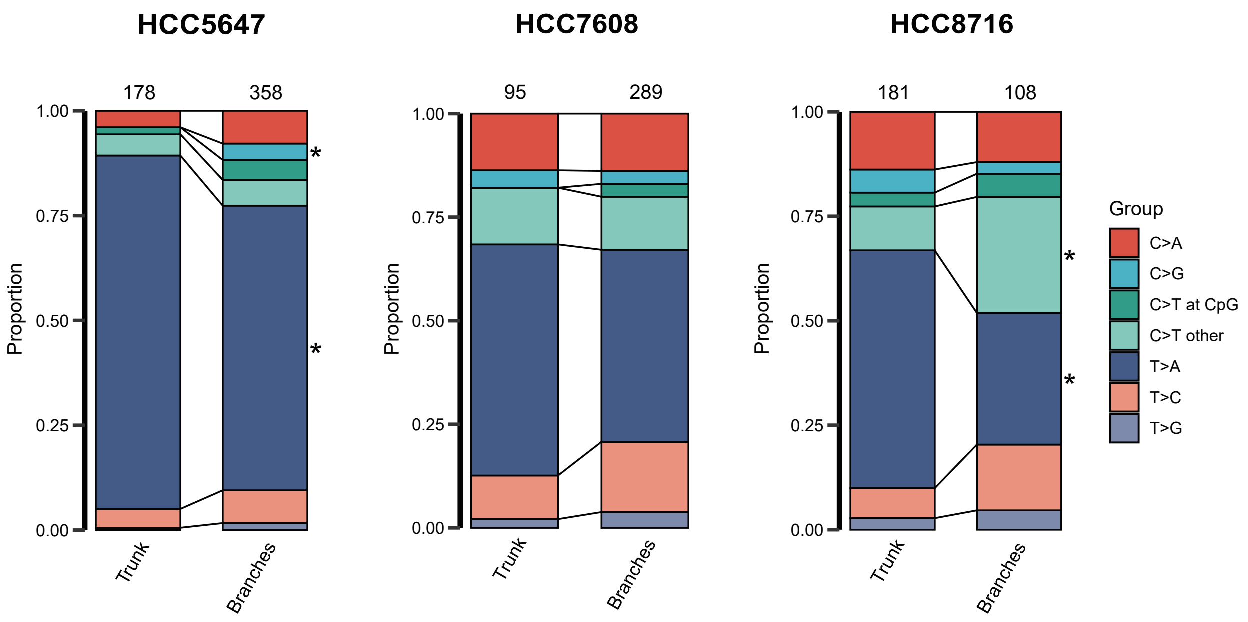
HCC5647
HCC7608
HCC8716
178
358
95
289
181
108
Proportion
1.00
0.75
0.50
0.25
0.00
*
Trunk
Branches
Group
C>A
C>G
C>T at CpG
C>T other
T>A
T>C
T>G

Figure S6. Schematic diagram of visualizing phylogenetic trees

Node N refers to a non-mutated normal sample: node 0 represents the starting node.

In tree T_0: $K = \{node\ 0, node\ 2, node\ 4, node\ 5, node\ 8\}$, $K^{[1]}$ is $node\ 0$;

$B = \{node\ 1,\ node\ 3,\ node\ 6,\ node\ 7\}$, $B^{[1]}$ is $node\ 1$; $R = \{node\ 1,\ node\ 7\}$, $R^{[1]}$ is $node\ 1$;

$L = \{node\ 3, node\ 6\}$, $L^{[1]}$ is $node\ 3$

Figure S6

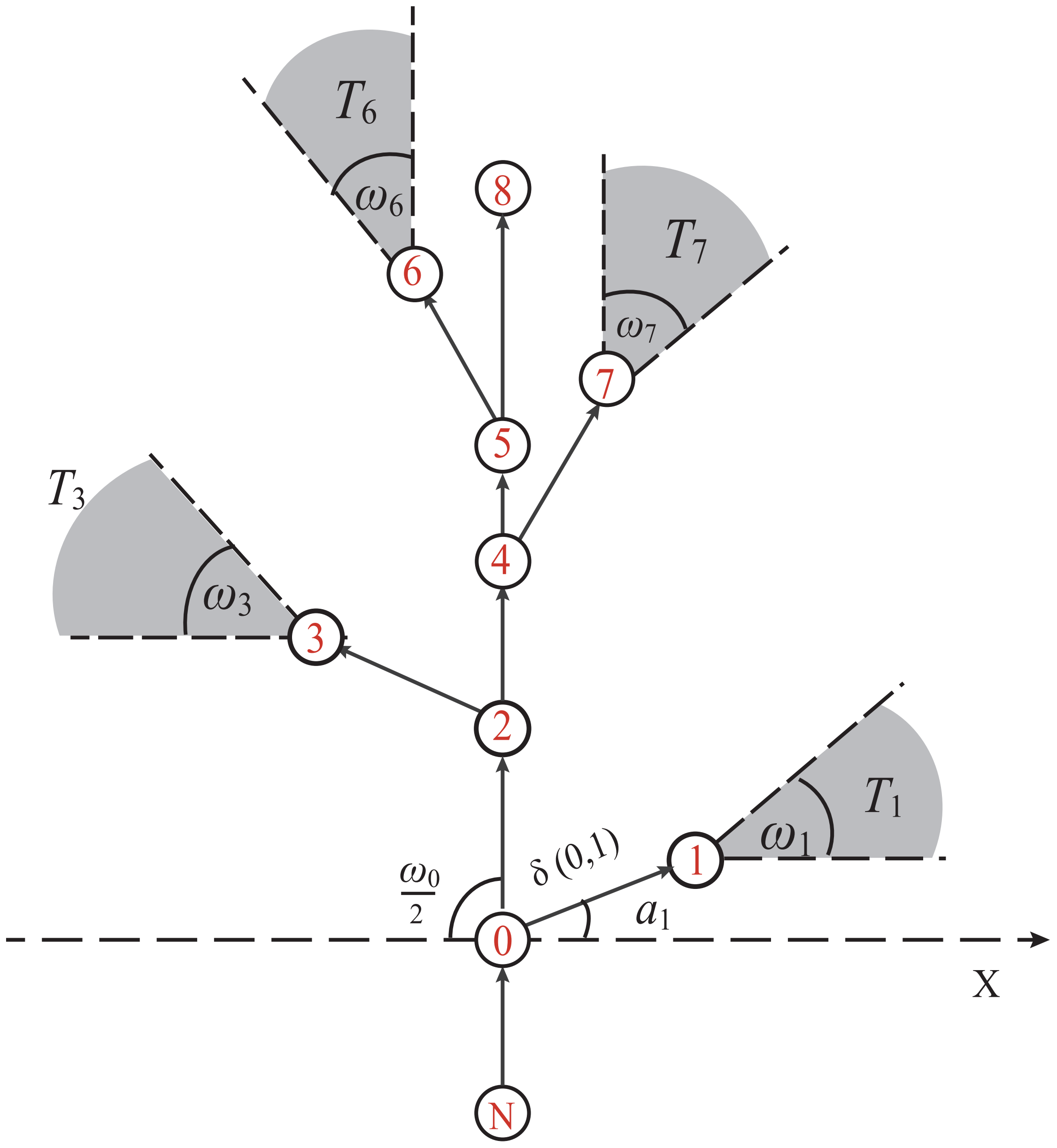

Supplement: giab036_Supplemental_Files [file giab036_supplemental_files.zip › Supplementary Method and Figures.pdf]
