# Supplementary material for: MesKit: a tool kit for dissecting cancer evolution of multi-region tumor biopsies through somatic alterations
Source: Gigascience. 2021 May 21;10(5):giab036. doi: 10.1093/gigascience/giab036 (PMC8138830; doi:10.1093/gigascience/giab036)
Supplement: giab036_Supplemental_Files [file giab036_supplemental_files.zip › Supplementary Table S1.docx]

**Table S1 Clinical features of the HCC cohort and CRC cohort**

| **Cohort** | **PatientID** | **Sex** | **Age at diagnosis of primary tumor** | **Diagnosis history** | **Tumor Type** | **Total number of samples** | **Data source** |
| --- | --- | --- | --- | --- | --- | --- | --- |
| HCC_LDC | HCC5647 | M | 40 | - | Primary (4) | 4 | Lin et al, 2017 |
| HCC_LDC | HCC6690 | M | 66 | - | Primary (5) | 5 | Lin et al, 2017 |
| HCC_LDC | HCC6046 | M | 48 | - | Primary (5) | 5 | Lin et al, 2017 |
| HCC_LDC | HCC6952 | M | 32 | - | Primary (5) | 5 | Lin et al, 2017 |
| HCC_LDC | HCC8010 | M | 65 | - | Primary (5) | 5 | Lin et al, 2017 |
| HCC_LDC | HCC7608 | M | 38 | - | Primary (5) | 5 | Lin et al, 2017 |
| HCC_LDC | HCC8031 | F | 71 | - | Primary (5) | 5 | Lin et al, 2017 |
| HCC_LDC | HCC8213 | M | 45 | - | Primary (5) | 5 | Lin et al, 2017 |
| HCC_LDC | HCC8257 | M | 39 | - | Primary (5) | 5 | Lin et al, 2017 |
| HCC_LDC | HCC8392 | M | 71 | - | Primary (4) | 4 | Lin et al, 2017 |
| HCC_LDC | HCC8716 | M | 53 | - | Primary (4) | 4 | Lin et al, 2017 |
| CRC_HZ | 402 | F | 47 | P – BM (4y8m) | Primary (4), BM (4) | 8 | Hu et al, 2019 |
| CRC_HZ | V750 | M | 65 | P&LN&LI&LU – BM (0y6m) | Primary (5), BM (5), LN (3) | 13 | Hu et al, 2019 |
| CRC_HZ | V824 | M | 61 | P&LN – BM&LU(0y10m) | Primary (3), BM (3), LN (2) | 9 | Hu et al, 2019 |
| CRC_HZ | V930 | F | 71 | P – LI (2y2m) – LU (5y8m) –BM(8y7m) | Primary (5), BM (5), LU (3) | 13 | Hu et al, 2019 |
| CRC_HZ | V953 | F | 68 | P – BM (2y6m) | Primary (3), BM (4) | 7 | Hu et al, 2019 |
| CRC_HZ | V974 | F | 60 | P&BM – RecBM (0y5m) | Primary (3), BM (5) | 8 | Hu et al, 2019 |
