# Supplementary material for: MesKit: a tool kit for dissecting cancer evolution of multi-region tumor biopsies through somatic alterations
Source: Gigascience. 2021 May 21;10(5):giab036. doi: 10.1093/gigascience/giab036 (PMC8138830; doi:10.1093/gigascience/giab036)
Supplement: giab036_Response_to_Reviewer_Comments_Original_Submission [file giab036_response_to_reviewer_comments_original_submission.pdf]

Author's Response To Reviewer Comments

Close

Dear editor and reviewers

We are very grateful to the reviewers for their careful reading of the manuscript and constructive remarks. We have addressed all the issues raised by the reviewers and paid heed to their advices and suggestions. The manuscript has been massively revised. Please find below a point-by-point reply to the reviewers' comments.
According to your suggestion, we included RRID (SCR_020959) and bio.tools ID (meskit) in the manuscript. In addition, we have sent our revised manuscript to professional English-editing service provider (AJE) for improvement of the manuscript. We believe that the contents and the clarity of our paper are much improved in the revised version.

Point by point response to revisers

Reviewer #1:
In their paper 'MesKit: a tool kit for dissecting cancer evolution of multi-region tumor biopsies through somatic alterations', the authors describe a new R package for analysis of multi-region tumour sequencing data (MRS) which comprises many of the commonly employed workflows and statistics into a convenient toolset.
I believe this is a useful and interesting piece of software, which might find broad application in the field. I particularly like the assembly of different heterogeneity measures within and between tumours. The tool is well documented and the online vignette is detailed and well made. I have only a few points that I would like to see addressed.

[Response]: Thank you very much for the positive and constructive comments. We have duly followed your comments and suggestions to address all concerns as elaborated in the following responses. In particular, we would like to thank you for your constructive comments which have improved the manuscript in numerous ways. Thank you very much for your precious time and kind attention.

Major points:
=============

1.) The authors position their tool as a general analysis toolkit for MRS datasets. However, the tool seems very much focused on somatic point mutations and provides little in terms of somatic-copy-number alteration (SCNA) analysis. SCNAs seem to be mainly used to correctly estimate CCFs. This is fine, but it needs to be stated clearly. I recommend extending the introduction to include and distinguish between heterogeneity measures and phylogeny reconstructions on SCNAs and those on SNVs. This way, the authors can give the reader a better understanding what MesKit does and does not provide.

[Response]: Thank you for this helpful comment. To describe the characteristics of MesKit more clearly, we modified the Introduction as follow: "MesKit was designed as an easy-to-use R package that only requires a Mutation Annotation Format (MAF) file and a clinical file as inputs, enabling researchers to evaluate the contribution of point mutations to heterogeneity within/between tumors from the same patient. MesKit can also be used to depict mutational profiles, track evolutionary dynamics and characterize mutational patterns at different
levels.".

2.) The discussion should also be adjusted accordingly. Please be more critical and make clear what the current drawbacks of MesKit are and where it might not be applicable. Again, bring up the point from the introduction, that MesKit is SNV-centric and that support for SCNA analysis so far is limited.

[Response]: Thank you for this valuable comment. According to your suggestion, we have revised the

discussion as follow: "As MesKit takes a MAF file and a clinical data file as standard inputs, it primarily evaluates ITH based on somatic mutations, and its assessment of contributions of CNAs is still limited. Currently, several subclonal reconstruction methods are available to infer the relative order of occurrence between a SNV and its associated CNA. In future updates, we plan to implement the integration of results from these methods to provide insight into the clonality and temporal dynamics of ITH…"

3.) The criterion for calling a SNV subclonal based on the merged CCF is unclear to me. There might be more information in the refs provided, but the paper needs to stand alone, so the authors need to at least provide a general idea of the motivation behind a 'merged CCF' and the specific cutoff of 0.5.

[Response]: We apologize for the confusion. Accordingly, we made a more detailed explanation about the "merged CCF" in the revised manuscript as follows (Methods-Clonal status of somatic mutations): "Since multiple samples collected from a single tumor collectively reflect its clonal composition, these regions should be considered as a whole to capture the overall tumor dynamics. Here, we assume that each tumor follows neutral exponential growth in a well-mixed population [39, 40]. When MRS data are available, the merged CCF (CCF_merged) of each mutation is computed by integrating multiple regions as previously described [12, 41, 42]…". And the CCF cutoff of "0.5" was chosen for its good performance in defining subclonality based on simulated virtual tumors, as demonstrated by Sun et al. Nature Genetics, 2017 [1].

Minor points:
=============

* Introduction, paragraph 1: "quantitative interpretation of heterogeneity..."I don't understand that sentence.

[Response]: Sorry for the confused phrase. We have corrected it accordingly.

* page 6: "visualization for MRS dataset yet" -> "datasets" (plural)

[Response]: Thank you very much for pointing this out. We have corrected it accordingly.

* Material and Methods (page 7): "Each sample was sequenced [...] to high coverage". It is very subjective what comprises high coverage. For example TRACERx sequencing coverage is > 400x. Please remove 'high'.

[Response]: Thank you very much for pointing this out. We have corrected it accordingly.

* Material and Methods (page 7): 'CRC cohort consisted of [...]' -> 'The CRC cohort...'

[Response]: Thank you very much for pointing this out. We have corrected it accordingly.

* Estimation of ITH: 'Fst' should be capitalised throughout (FST)

[Response]: Thank you very much for pointing this out. We have corrected it accordingly.

* Inference of metastatic routes: 'which identifies subclones shared between different regions via CCF plots': I was expecting a Figure reference here. Please provide one that shows a CCF plot and what it does.

[Response]: Thanks for your valuable comments. According to your suggestion, we have added a reference of CCF plot and revised manuscript as follow (Results-Inferring the clonality of metastatic seeding): "Given that mutations with similar CCFs tend to cluster into the same subpopulation [69, 70], many studies inferred the potential metastatic routes between different lesions from the same patient by plotting CCFs of mutations [12, 42, 71]. By this means Xue et al. [71] identified both monoclonal and multiclonal origins of separate type combined hepatocellular and intrahepatic cholangiocarcinoma (cHCC-ICC)."

Reviewer #2:
In this manuscript, Liu et al present MesKit, an R package designed to study tumor heterogeneity through multiple samples sequencing, with examples coming from published data in hepatocellular

carcinoma and colorectal cancer. The package is already available in R/Bioconductor, with an example script including embedded data, allowing anyone to test the functionalities. MesKit represents a useful tool since it wraps together multiple methods already described in various publications, and allows the usage of simple inputs (maf files) to study tumor heterogeneity. Moreover, a shiny application has been developed by the authors, allowing usage of the tool by researchers lacking bioinformatics skills.

[Response]: Thank you very much for the constructive and positive comments. We have duly followed your comments and suggestions to address all concerns elaborately in the following responses.

Here are my comments:

Q1. The formula used to calculate the AUC of CCF is not correct, therefore the values obtained are underestimated: in the ccfAUC function, the integration is done between min(df_ccf$CCF) and max(df_ccf$CCF) but it should be done between 0 and 1. For instance, the AUC of HCC8010_T1 (Fig3B) is missing all the area between 0.50 and 1, and therefore it should be the highest of the patient instead of being the smallest.

[Response]: Thanks for pointing out this mistake, we have corrected it accordingly.

Q2. For the vafCluster function (Fig3A), it is not really clear if the function takes into account the multiple regions and thus does a clustering in multiple dimensions, or just does independent Gaussian mixture in one dimension for each region. Is there a relation between the clusters (1-6) defined in each region? In any case, I'm not sure that the Gaussian finite mixture model is a good way to separate clusters of mutations corresponding to subclones. The usual approach since the seminal paper of Nik-Zainal et al, Cell 2012 is to use a Dirichlet process.

[Response]: We apologize for the confusion. The clusters of different regions are uncorrelated. The vafCluster function performs clustering in one dimension for each region/tumor based on the independent Gaussian mixture model. This model has been employed in multiple studies for 1D clustering [2, 3]. Furthermore, considering the clustering of observed VAFs without consideration of copy number may not be informative, we have modified the clustering strategy implemented in the function vafCluster and renamed it to mutCluster for accuracy and relevance. Details of this analysis were revised in the manuscript as follow (Methods-ITH estimation): "For a single region/tumor, it is common to infer subpopulations of tumor cells by clustering VAFs or CCFs [26, 30]. To implement this process, we employed Gaussian finite mixture models for one-dimensional clustering of VAFs or CCFs using mclust R package [46]. As copy number gains and losses can alter the fraction of reads bearing a mutation, we only focused on heterozygous mutations within copy-number neutral and loss of heterozygosity (LOH)-free regions when clustering VAFs.". We have also made this clear in the vignette. Additionally, a prompting message will be printed when calling the function mutCluster as "## Performing one-dimensional clustering for …".
Although many existing methods rely on some form of "Dirichlet process" clustering, such as sciClone [4], DPClust [5], PyClone [6] and PhyloWGS [7], there is still a lack of thorough assessment of them. We will continue to pay attention to this fast-evolving field and consider the integration of clustering results of other tools in future updates. Thanks again for your valuable comments!

Q3. Relatedly, the 2-dimension representation of CCF (Fig3D) would clearly benefit of an estimation of clusters corresponding to subclones, either through a Dirichlet process, as performed in Gundem et al 2015, or just a clustering of the mutations in multiple dimensions. It would facilitate the interpretation of the plots and the evaluation of the monoclonal or polyclonal seeding of metastasis.

[Response]: Thanks for your comments. Yes, Gundern et al performed two-dimensional clustering between metastatic sites, but our intension was to present comparison between lesions (primary tumor/metastasis pair) with multiple sites sequenced. To our knowledge, no appropriate method has been specifically developed for comparison of paired lesions with MRS data. To address your concerns, we employed SciClone [4] to cluster merged VAFs (equivalent to CCF_merged/2, see Methods for details) of each primary/metastasis tumor pair. As shown in Figure R1 (https://github.com/Niinleslie/MesKit/blob/paper/inst/plot/Figure_R1.md), mutations were indicated in different shapes according to the cluster they correspond to, which was consistent with the density-based distribution. Mutations with similar CCFs density were likely to cluster together by SciClone [4]. Still, we will continue to pay attention to this fast-evolving field and consider the integration of clustering results of other tools in future updates. Thanks again for your valuable comments!

[Figure R1] To note, as the submission system did not support figure, we have linked the figure with an external link, which could be found at
https://github.com/Niinleslie/MesKit/blob/paper/inst/plot/Figure_R1.md
Figure R1. Density plots of merged CCF values in paired primary tumors and metastases of the CRC cohort. SciClone was employed to cluster merged VAFs (equivalent to CCF_merged/2) for each primary tumor/metastasis sample pair and mutations were indicated in different shapes according to the cluster they correspond to. Jaccard similarity index (JSI) and putative CRC driver genes are indicated on the plot. P, primary tumor; BM, brain metastasis; LN, lymph node metastasis; LU, lung metastasis.

Q4. Some of the data used for examples are a little odd:

[Response]: Thanks! Your comments reminded us that there were potential artifacts in the datasets of the HCC cohort. To provide a better demonstration of MesKit, we amended the analysis pipeline and adopted more rigorous criteria for mutations calling and CNA detection. The "Method-Data collection and preprocessing" section was revised as follow: "In brief, we performed sSNV calling for each tumor/normal pair with Mutect (version 1.1.7) [33], while INDELs were detected with Strelka v2.7.1 [34]. Additionally, we adopted the "force calling" method [35] to rescue potential real mutations for each sample based on the aggregate set of somatic events in each patient using samtools mpileup (version 1.2) [36]. Both sSNVs and INDELs were annotated through ANNOVAR (v.20191024) [37]. The following filters were further applied to identify the sSNVs and INDELs: (1) Mutations with fewer than 15 total reads or 5 variant reads were discarded. (2) Mutations listed in dbSNP147 were removed unless they were documented in the Catalogue of Somatic Mutations in Cancer (COSMIC) database. (3) Mutations listed in the National Heart, Lung, and Blood Institute Exome Sequencing Project were removed. Copy number analysis based on exome sequencing was performed using Sequenza v3.0.0 [38]. Segments smaller than 500 kb were filtered and only autosomes were used in copy number analysis. CCFs of mutations were estimated by PyClone (v0.13.0) [28], which adjusted the variant allele frequencies (VAFs) of somatic mutations based on local copy numbers of the mutated loci and tumor purity (Sequenza v3.0.0) [38].". Accordingly, we revised the corresponding results about the HCC cohort and redrew the following figures: Figure 2B, Figure 3A, B, Figure 5, Figure S1A and Figure S5.

i) in the HCC mutational landscape (Fig S1A), the frequency of KMT2C alterations is really high (69%) compared to known cohorts of HCC (< 10%), and may represent artifacts.

[Response]: Thank you very much for pointing this out. According to your suggestion, we have recalled somatic mutations as answered in Q4 and redrew the mutational landscape with updated mutation frequencies of the CRC cohort (Figure S1A). Thanks again for your comments.

ii) in the CNA data (Fig2B), there are really large deletions in dark blue, corresponding to CN=0, which is not possible for an entire chromosome, so the copy-number is probably wrong.

[Response]: Thanks for your comments. We have redrawn Figure2B based on CNAs detected by Sequenza (v3.0.0), see the response of Q4 for details. Besides, this part was revised in manuscript as follow (Results-Mutational landscape from MRS studies): "Consistent with TCGA projects and other previous studies of hepatocellular carcinoma [65, 66], a number of copy number alterations were observed in our HCC cohort, such as gains of 1q, 6p, 8q and 13q as well as losses of 1p, 4q, 9q and 11q (Figure 2B).".

iii) in the CCF distribution of HCC8010 samples, region T1 has only mutations with CCF < 0.5, i.e not a single clonal mutation. This is likely an artifact due to a wrong estimation of the tumor purity used to obtain CCF from VAF data.

[Response]: Thanks for your comments. We have redrawn the CCF distribution of HCC8010 (Figure 3B).

-> it would be better to have cleaner data for the examples, however this doesn't represent a problem of the tool itself, just a problem of the data used as input.

[Response]: We thank the reviewer for the valuable suggestion. We have re-analyzed the datasets of the HCC cohort as mentioned above. Please refer to the above response of Q4 for details.

Q5. It is really useful to be able to run examples from the package, however the data obtained sometimes differ from the one represented in the figures: for instance, the MATH values for HCC8010 samples are really different in the R example and Fig3A (T1 is 83.3 instead of 5.7), and the density plots

of VAF for CHC8010 have really different profiles. Also, the AUC of CCF for HCC8010_T5 is 0.429 in Fig3B but 0.462 when running the R example script. There should be consistency between the figures plotted in the paper and the one obtained by running the script, or at least the difference should be explained.

[Response]: Thanks for your suggestion, we have uploaded all related scripts on CodeOcean. In terms of the inconsistency you mentioned here, it is likely that you used default parameters instead of those used for figures in the paper. We suggest running code in the CodeOcean for verification (https://codeocean.com/capsule/4694382/tree/v2).

Q6. For the clustering of mutational signatures (Fig4B), it might be more relevant to directly cluster the mutation matrix for the 96 trinucleotides changes, instead of adding the step of the cosine similarity with COSMIC signatures. Indeed, this extra step adds a level of approximation, and some mutations might account for multiple signatures (for instance the numerous T>A mutations account for signature 22 and signature 25 in HCC patients exposed to aristolochic acid).

[Response]: Thanks for your comments. Yes, clustering the mutation matrix is a more direct way to compare the mutational profiles between samples. However, we presented the "clustering of mutational signatures" for its own advantages, as it can directly show the similarity in mutation profiles between samples to some degree, while at the same time providing information on which signatures are likely active. And this method has been previously used in MutationalPatterns [8].

Q7. I have no expertise in the Wright's fixation index FST, but looking at formula (10) of the article Bhatia et al, 2013, it seems that the formula (4) of the manuscript is missing a minus. Also the term mt is not defined, and the binomial coefficient (2 k) is inverted, the k should be on top. The same comment applies for Nei's distance, formulas (5) and (6).

[Response]: Thank you very much for pointing this problem out. We have corrected the formula and added the definition of "mt" in our revised manuscript as follows: "m_t represents the total number of sSNVs in region a and b".

Minor comments:
- In Figure 1, the optional inputs should include a segmented copy-number file.

[Response]: Thanks for pointing this out. We have corrected it accordingly.

- In sup Table 1, data source for HCC samples is De-Chen et al, 2017 instead of Lin et al, 2017.

[Response]: We thank the reviewer for pointing out this mistake. We have corrected it accordingly.

- In the introduction, the authors could cite the Palimpsest package (Shinde et al, 2018) since it allows the identification of different mutational signatures between clonal and subclonal mutations.

[Response]: Thanks for your suggestion. We have added the citation accordingly.

- In formula (7), the value of the original reference is 1.4826 instead of 1.4268, and I don't understand why there is an "avg" subscript for VAF.

[Response]: We deeply apology for this typo error and we have corrected it accordingly.

- Signatures of HCC are in Table S2 and not File S2.

[Response]: Thanks for pointing this out. We have corrected it accordingly.

Reviewer #3:
The authors present a software tool kit called MesKit that analyses multi region sequencing studies of cancer. The tool is quite comprehensive with many different capabilities, good documentation and produces high-quality figures. Overall, I think this would be a valuable piece of software to the cancer evolution community. I do have a few concerns with regard to the interpretation of some of these analyses which I believe should be appropriately caveated.

[Response]: Thank you very much for the critical and helpful comments. We have duly followed your comments and suggestions to address all the concerns as elaborated in the following responses.

Major comments

Q1. MesKit enables clustering by VAF to interpret ITH. Without consideration of the copy number of the mutation, VAF clustering will not be informative. The authors should clarify what they are doing here. It is more appropriate to do one of the following: i) cluster using CCFs ii) cluster using some kind VAF corrected by copy number or iii) cluster VAF per copy number state (cluster on mutation only found in diploid regions for example). Another relevant point here is that there is a debate within the field around the use of clustering to identify subpopulation, see for example Caravagna et al Nature Genetics 2020. The main issue is that "clusters" at low CCF/VAF may not represent distinct populations but rather are a mixture of variants present within different lineages. Another issue is that depth of sequencing can have a large influence on the quality of clustering results (again see Caravagna et al Nature Genetics 2020). I think the challenges and possible issues with this type of analysis should be mentioned in the paper and software vignette so that users understand the caveats here.

[Response]: Thanks for your valuable suggestions. We agree that clustering of estimated VAFs is not informative without adjustment of copy number. According to your suggestion, we modified the clustering strategy implemented in the ¬¬vafCluster and renamed it to mutCluster for accuracy and relevance. The mutCluster function performs one-dimensional clustering of mutations by VAFs and CCFs. When clustering VAFs, this function only focuses on heterozygous mutations within normal diploid regions. We have made a detailed description about this analysis in the revised manuscript as follow (Methods-Estimation of ITH): "For a single region/tumor, it is common to infer subpopulations of tumor cells by clustering VAFs or CCFs [26, 30]. To implement this process, we employed Gaussian finite mixture models for one-dimensional clustering of VAFs or CCFs using mclust R package [46]. As copy number gains and losses can alter the fraction of reads bearing a mutation, we only focused on heterozygous mutations within copy-number neutral and loss of heterozygosity (LOH)-free regions when clustering VAFs." Besides, we agree that debates and challenges of this analysis should be emphasized. We thus highlighted the debate about "low-frequency populations" and added related citations[9, 10] in the "Results-ITH estimation" section as follow: "In MesKit, the mutCluster function deduces distinct subpopulations of a sample/tumor by clustering VAFs/CCFs in one dimension based on Gaussian finite mixture models [46]. It should be noted that low-frequency clusters might be a mixture of subclones that contain mutations coming from numerous parallel lineages growing neutrally [32, 68]". Furthermore, we added the following sentence to mention the depth issue in the Introduction: "In general, high-depth sequencing improves subclonal reconstruction accuracy and resolution [32].".

Q2. I think it is highly unlikely that the BRCA2 mutations the authors identify contribute to metastasis in CRC. To probe this further the authors should assess the functional impact of the mutation through cbioportal or the like and assess the copy number zygosity around that locus, appearance of LOH may give some further evidence that these mutations are functional. In general, there is little evidence that BRCA2 has a role in CRC development, see Jonsson et al. Nature 2019.

[Response]: Thanks for your valuable comments. We identified two nonsynonymous mutations in BRCA2 in metastases samples of V824 and V930 (chr13:32954205:A:T and chr13: 32911442:-:A), both of which are heterozygous (without LOH) and located in amplified regions. However, we agree that there is no strong evidence that demonstrates BRCA2 mutations contribute to CRC metastasis yet. To be more rigorous, we revised this part in "Results-Mutational landscape of MRS studies" section as follow: "Interestingly, heterozygous BRCA2 mutations were private to distant metastases, including the lung metastases (LU) and brain metastases (BM) of two patients (V824 and V930), while there is currently no strong evidence that shows BRCA2 mutations are associated with CRC metastasis."

Q3. I would like to see some comparison of the signature deconvolution with other methods to ensure that the authors implementation is approximately equivalent to other widely used tools.

[Response]: Thank you very much for the insights. We, therefore, addressed this deficiency by comparing the performance of figSignatures function against other three signature deconvolution methods. We provided additional files (Figure S3 and Table S3) and described the details of this analysis in the "Results-Temporal dissection of mutational signatures" section as follow: "Therefore, we developed the fitSignatures function to calculate the contribution of well-established signatures to mutations at different levels. By reconstructing the mutational profiles of the HCC and CRC cohorts using 30 COSMIC mutational signatures, we demonstrated that the signature contributions estimated by

fitSignatures function were highly similar to those calculated by three other signature deconvolution tools (average Pearson correlation: 1-MutationalPatterns [23]; 0.997-SignatureEstimation [74]; 0.948-deconstructSigs [24]) (Figure S4A and Table S3). The similarities (indicated by cosine similarity) and discrepancies (indicated by RSS) between the original and reconstructed mutational profiles generated with MesKit were also comparable to those generated from other tools (Figure S4B)."

Minor comments

There are a number of grammatical errors that need addressing.

[Response]: Sorry for the grammatical errors. We have sent our revised manuscript to AJE (https://www.aje.cn) asking for improvement of the manuscript.

* The ability to generate phylogenetic trees with different methods is very valuable in my opinion. In the methods the authors comment on how MesKit provides the ability to compare tree similarity using a function called treedist, this is not used in the results as far as I can see. I think including some comparison in the results would be a valuable illustration of how similar (or different) differing approaches may be.

[Response]: Thanks for your valuable suggestion. We agree that results would benefit from a comparison of phylogenetics trees constructed by different methods. We have added this analysis in the "Results-Construction and visualization of phylogenetic trees" section as follow: "Moreover, we compared the MP-based phylogenetic trees with those constructed by the NJ method and ML method for each CRC patient. Phylogenetic trees inferred through the three methods shared the same topology and clades for V402, V924 and V953 (Figure S3). When considering branch lengths, the MP-based trees were more similar to the NJ-based trees than the ML-based trees according to KF-branch distance [72] and weighted RF distance [74] (Table S2).". Additional figure (Figure S3) and table (Table S2) were provided for better illustration and explanation.

* It says in the text that the software is released under the GPL v3 License, but in the github repo, an MIT license is shown. This should be clarified.

[Response]: Thank you for pointing this problem out. MesKit was released under the GPL v3 and we have corrected it in the github repository.

* I was able to install the software via devtools but not bioconductor, it appears others have had similar issues - https://github.com/Niinleslie/MesKit/issues/211. Would be good to understand why this might be happening.

[Response]: MesKit was released on the version 3.12 of Bioconductor, which matches R 4.0.3. This problem would be solved when you update Bioconductor to version 3.12 (or higher). Besides, we have made this clear in the revised documentation.

* In the introduction, I think several commonly used ITH related tools are missing citations such as pyclone, DPclust and phylowgs.

[Response]: Thanks for pointing this problem out. Accordingly, we included corresponding citations and revised the Introduction as follows: "…In addition, lots of methods infer tumor heterogeneity by assessing the complex subclonal structure of tumors. Approaches such as SciClone [26], PhyloSub [27] and PyClone [28] are based solely on point mutations (sSNVs or INDELs), while SCHISM [29], DPClust [30] and PhyloWGS [31] adjust for CNAs in their models in different ways. In general, high-depth sequencing improves the accuracy of subclonal reconstruction and resolution [32]"

* This is not mentioned in the paper, but in the R package the authors provide the ability to perform a "neutrality test" as described in my paper from 2016: Williams et al Nature Genetics. This analysis has been superseded by a new software tool called MOBSTER (https://github.com/caravagnalab/mobster), I think this should be made clear in the vignette so that users know what the current recommended analysis tools are for this type of analysis. I would not expect a new implementation of MOBSTER, just a note in the vignette and function documentation would suffice.

[Response]: Thanks for your suggestion. At the beginning of the project, there was no such software or

package providing such analysis. This function was retained during the development periods. We have made it clear in the vignette and added proper citation accordingly.

* Hu et al. Nature Genetic should be cited when describing Fst, this was the first (only?) study to use this measure to my knowledge and should be cited accordingly.

[Response]: Thanks for your comments. We have added the citation accordingly.

Reference for point-by-point response

1. Sun R, Hu Z, Sottoriva A, Graham TA, Harpak A, Ma Z, et al. Between-region genetic divergence reflects the mode and tempo of tumor evolution. Nat Genet. 2017;49 7:1015-24. doi:10.1038/ng.3891.
2. Mayakonda A, Lin D-C, Assenov Y, Plass C and Koeffler HP. Maftools: efficient and comprehensive analysis of somatic variants in cancer. Genome research. 2018;28 11:1747-56. doi:10.1101/gr.239244.118.
3. Patel AP, Tirosh I, Trombetta JJ, Shalek AK, Gillespie SM, Wakimoto H, et al. Single-cell RNA-seq highlights intratumoral heterogeneity in primary glioblastoma. Science. 2014;344 6190:1396-401. doi:10.1126/science.1254257.
4. Miller CA, White BS, Dees ND, Griffith M, Welch JS, Griffith OL, et al. SciClone: inferring clonal architecture and tracking the spatial and temporal patterns of tumor evolution. PLoS Comput Biol. 2014;10 8:e1003665. doi:10.1371/journal.pcbi.1003665.
5. Nik-Zainal S, Van Loo P, Wedge DC, Alexandrov LB, Greenman CD, Lau KW, et al. The life history of 21 breast cancers. Cell. 2012;149 5:994-1007. doi:10.1016/j.cell.2012.04.023.
6. Roth A, Khattra J, Yap D, Wan A, Laks E, Biele J, et al. PyClone: statistical inference of clonal population structure in cancer. Nat Methods. 2014;11 4:396-8. doi:10.1038/nmeth.2883.
7. Deshwar AG, Vembu S, Yung CK, Jang GH, Stein L and Morris Q. PhyloWGS: reconstructing subclonal composition and evolution from whole-genome sequencing of tumors. Genome Biol. 2015;16:35. doi:10.1186/s13059-015-0602-8.
8. Blokzijl F, Janssen R, van Boxtel R and Cuppen E. MutationalPatterns: comprehensive genome-wide analysis of mutational processes. Genome Med. 2018;10 1:33. doi:10.1186/s13073-018-0539-0.
9. Williams MJ, Werner B, Heide T, Curtis C, Barnes CP, Sottoriva A, et al. Quantification of subclonal selection in cancer from bulk sequencing data. Nat Genet. 2018;50 6:895-903. doi:10.1038/s41588-018-0128-6.
10. Caravagna G, Heide T, Williams MJ, Zapata L, Nichol D, Chkhaidze K, et al. Subclonal reconstruction of tumors by using machine learning and population genetics. Nat Genet. 2020;52 9:898-907. doi:10.1038/s41588-020-0675-5.

Close
