# Supplementary material for: MesKit: a tool kit for dissecting cancer evolution of multi-region tumor biopsies through somatic alterations
Source: Gigascience. 2021 May 21;10(5):giab036. doi: 10.1093/gigascience/giab036 (PMC8138830; doi:10.1093/gigascience/giab036)
Supplement: giab036_Reviewer_1_Report_Original_Submission — Roland Schwarz -- 1/31/2021 Reviewed [file giab036_reviewer_1_report_original_submission.pdf]

Reviewer Report

Title: MesKit: a tool kit for dissecting cancer evolution of multi-region tumor biopsies through somatic alterations

Version: Original Submission **Date:** 1/31/2021

Reviewer name: Roland Schwarz

Reviewer Comments to Author:

In their paper 'MesKit: a tool kit for dissecting cancer evolution of multi-region tumor biopsies through somatic alterations', the authors describe a new R package for analysis of multi-region tumour sequencing data (MRS) which comprises many of the commonly employed workflows and statistics into a convenient toolset.
I believe this is a useful and interesting piece of software, which might find broad application in the field. I particularly like the assembly of different heterogeneity measures within and between tumours. The tool is well documented and the online vignette is detailed and well made.
I have only a few points that I would like to see addressed.
Major points:
=============
1.) The authors position their tool as a general analysis toolkit for MRS datasets. However, the tool seems very much focused on somatic point mutations and provides little in terms of somatic-copy-number alteration (SCNA) analysis. SCNAs seem to be mainly used to correctly estimate CCFs. This is fine, but it needs to be stated clearly. I recommend extending the introduction to include and distinguish between heterogeneity measures and phylogeny reconstructions on SCNAs and those on SNVs. This way, the authors can give the reader a better understanding what MesKit does and does not provide.
2.) The discussion should also be adjusted accordingly. Please be more critical and make clear what the current drawbacks of MesKit are and where it might not be applicable. Again, bring up the point from the introduction, that MesKit is SNV-centric and that support for SCNA analysis so far is limited.
3.) The criterion for calling a SNV subclonal based on the merged CCF is unclear to me. There might be more information in the refs provided, but the paper needs to stand alone, so the authors need to at least provide a general idea of the motivation behind a 'merged CCF' and the specific cutoff of 0.5.

Minor points:
=============
* Introduction, paragraph 1: "quantitative interpretation of heterogeneity..."
I don't understand that sentence.
* page 6: "visualization for MRS dataset yet" -> "datasets" (plural)
* Material and Methods (page 7): "Each sample was sequenced [...] to high coverage". It is very subjective what comprises high coverage. For example TRACERx sequencing coverage is > 400x. Please remove 'high'.
* Material and Methods (page 7): 'CRC cohort consisted of [...]' -> 'The CRC cohort...'
* Estimation of ITH: 'Fst' should be capitalised throughout (FST)
* Inference of metastatic routes: 'which identifies subclones shared bewteen different regions via CCF plots': I was expecting a Figure reference here. Please provide one that shows a CCF plot and what it does.

Level of Interest

Please indicate how interesting you found the manuscript: Choose an item.

Quality of Written English

Please indicate the quality of language in the manuscript: Choose an item.

Declaration of Competing Interests

Please complete a declaration of competing interests, considering the following questions:

- Have you in the past five years received reimbursements, fees, funding, or salary from an organisation that may in any way gain or lose financially from the publication of this manuscript, either now or in the future?
- Do you hold any stocks or shares in an organisation that may in any way gain or lose financially from the publication of this manuscript, either now or in the future?
- Do you hold or are you currently applying for any patents relating to the content of the manuscript?
- Have you received reimbursements, fees, funding, or salary from an organization that holds or has applied for patents relating to the content of the manuscript?
- Do you have any other financial competing interests?
- Do you have any non-financial competing interests in relation to this paper?

If you can answer no to all of the above, write 'I declare that I have no competing interests' below. If your reply is yes to any, please give details below.

I declare that I have no competing interests

I agree to the open peer review policy of the journal. I understand that my name will be included on my report to the authors and, if the manuscript is accepted for publication, my named report including any attachments I upload will be posted on the website along with the authors' responses. I agree for my report to be made available under an Open Access Creative Commons CC-BY license (http://creativecommons.org/licenses/by/4.0/). I understand that any comments which I do not wish to be included in my named report can be included as confidential comments to the editors, which will not be published.

Choose an item.

To further support our reviewers, we have joined with Publons, where you can gain additional credit to further highlight your hard work (see: https://publons.com/journal/530/gigascience). On publication of this paper, your review will be automatically added to Publons, you can then choose whether or not to claim your Publons credit. I understand this statement.

Yes Choose an item.
