# Supplementary material for: MesKit: a tool kit for dissecting cancer evolution of multi-region tumor biopsies through somatic alterations
Source: Gigascience. 2021 May 21;10(5):giab036. doi: 10.1093/gigascience/giab036 (PMC8138830; doi:10.1093/gigascience/giab036)
Supplement: giab036_Reviewer_2_Report_Original_Submission — Theo Z Hirsch, Ph.D -- 2/2/2021 Reviewed [file giab036_reviewer_2_report_original_submission.pdf]

Reviewer Report

Title: MesKit: a tool kit for dissecting cancer evolution of multi-region tumor biopsies through somatic alterations

Version: Original Submission **Date:** 2/2/2021

Reviewer name: Theo Z Hirsch, Ph.D

Reviewer Comments to Author:

In this manuscript, Liu et al present MesKit, an R package designed to study tumor heterogeneity through multiple samples sequencing, with examples coming from published data in hepatocellular carcinoma and colorectal cancer. The package is already available in R/Bioconductor, with an example script including embedded data, allowing anyone to test the functionalities. MesKit represents a useful tool since it wraps together multiple methods already described in various publications, and allows the usage of simple inputs (maf files) to study tumor heterogeneity. Moreover, a shiny application has been developed by the authors, allowing usage of the tool by researchers lacking bioinformatics skills.
Here are my comments:
- The formula used to calculate the AUC of CCF is not correct, therefore the values obtained are underestimated: in the ccfAUC function, the integration is done between min(df_ccf$CCF) and max(df_ccf$CCF) but it should be done between 0 and 1. For instance, the AUC of HCC8010_T1 (Fig3B) is missing all the area between 0.50 and 1, and therefore it should be the highest of the patient instead of being the smallest.
- For the vafCluster function (Fig3A), it is not really clear if the function takes into account the multiple regions and thus does a clustering in multiple dimensions, or just does independent Gaussian mixture in one dimension for each region. Is there a relation between the clusters (1-6) defined in each region? In any case, I'm not sure that the Gaussian finite mixture model is a good way to separate clusters of mutations corresponding to subclones. The usual approach since the seminal paper of Nik-Zainal et al, Cell 2012 is to use a Dirichlet process.
- Relatedly, the 2-dimension representation of CCF (Fig3D) would clearly benefit of an estimation of clusters corresponding to subclones, either through a Dirichlet process, as performed in Gundem et al 2015, or just a clustering of the mutations in multiple dimensions. It would facilitate the interpretation of the plots and the evaluation of the monoclonal or polyclonal seeding of metastasis.
- Some of the data used for examples are a little odd:
i) in the HCC mutational landscape (Fig S1A), the frequency of KMT2C alterations is really high (69%) compared to known cohorts of HCC (< 10%), and may represent artifacts.
ii) in the CNA data (Fig2B), there are really large deletions in dark blue, corresponding to CN=0, which is not possible for an entire chromosome, so the copy-number is probably wrong.
iii) in the CCF distribution of HCC8010 samples, region T1 has only mutations with CCF < 0.5, i.e not a single clonal mutation. This is likely an artifact due to a wrong estimation of the tumor purity used to obtain CCF from VAF data.
-> it would be better to have cleaner data for the examples, however this doesn't represent a problem

of the tool itself, just a problem of the data used as input.
- It is really useful to be able to run examples from the package, however the data obtained sometimes differ from the one represented in the figures: for instance, the MATH values for HCC8010 samples are really different in the R example and Fig3A (T1 is 83.3 instead of 5.7), and the density plots of VAF for CHC8010 have really different profiles. Also, the AUC of CCF for HCC8010_T5 is 0.429 in Fig3B but 0.462 when running the R example script. There should be consistency between the figures plotted in the paper and the one obtained by running the script, or at least the difference should be explained.
- For the clustering of mutational signatures (Fig4B), it might be more relevant to directly cluster the mutation matrix for the 96 trinucleotides changes, instead of adding the step of the cosine similarity with COSMIC signatures. Indeed, this extra step adds a level of approximation, and some mutations might account for multiple signatures (for instance the numerous T>A mutations account for signature 22 and signature 25 in HCC patients exposed to aristolochic acid).
- I have no expertise in the Wright's fixation index FST, but looking at formula (10) of the article Bhatia et al, 2013, it seems that the formula (4) of the manuscript is missing a minus. Also the term mt is not defined, and the binomial coefficient (2 k) is inverted, the k should be on top. The same comment applies for Nei's distance, formulas (5) and (6).
Minor comments:
- In Figure 1, the optional inputs should include a segmented copy-number file.
- In sup Table 1, data source for HCC samples is De-Chen et al, 2017 instead of Lin et al, 2017.
- In the introduction, the authors could cite the Palimpsest package (Shinde et al, 2018) since it allows the identification of different mutational signatures between clonal and subclonal mutations.
- In formula (7), the value of the original reference is 1.4826 instead of 1.4268, and I don't understand why there is an "avg" subscript for VAF.
- Signatures of HCC are in Table S2 and not File S2.

Level of Interest

Please indicate how interesting you found the manuscript: Choose an item.

Quality of Written English

Please indicate the quality of language in the manuscript: Choose an item.

Declaration of Competing Interests

Please complete a declaration of competing interests, considering the following questions:

- Have you in the past five years received reimbursements, fees, funding, or salary from an organisation that may in any way gain or lose financially from the publication of this manuscript, either now or in the future?
- Do you hold any stocks or shares in an organisation that may in any way gain or lose financially from the publication of this manuscript, either now or in the future?

- Do you hold or are you currently applying for any patents relating to the content of the manuscript?
- Have you received reimbursements, fees, funding, or salary from an organization that holds or has applied for patents relating to the content of the manuscript?
- Do you have any other financial competing interests?
- Do you have any non-financial competing interests in relation to this paper?

If you can answer no to all of the above, write 'I declare that I have no competing interests' below. If your reply is yes to any, please give details below.

I declare that I have no competing interests

I agree to the open peer review policy of the journal. I understand that my name will be included on my report to the authors and, if the manuscript is accepted for publication, my named report including any attachments I upload will be posted on the website along with the authors' responses. I agree for my report to be made available under an Open Access Creative Commons CC-BY license (http://creativecommons.org/licenses/by/4.0/). I understand that any comments which I do not wish to be included in my named report can be included as confidential comments to the editors, which will not be published.

Choose an item.

To further support our reviewers, we have joined with Publons, where you can gain additional credit to further highlight your hard work (see: https://publons.com/journal/530/gigascience). On publication of this paper, your review will be automatically added to Publons, you can then choose whether or not to claim your Publons credit. I understand this statement.

Yes Choose an item.
