# Supplementary material for: MesKit: a tool kit for dissecting cancer evolution of multi-region tumor biopsies through somatic alterations
Source: Gigascience. 2021 May 21;10(5):giab036. doi: 10.1093/gigascience/giab036 (PMC8138830; doi:10.1093/gigascience/giab036)
Supplement: giab036_Reviewer_3_Report_Original_Submission — Marc Williams -- 2/4/2021 Reviewed [file giab036_reviewer_3_report_original_submission.pdf]

Reviewer Report

Title: MesKit: a tool kit for dissecting cancer evolution of multi-region tumor biopsies through somatic alterations

Version: Original Submission **Date:** 2/4/2021

Reviewer name: Marc Williams

Reviewer Comments to Author:

The authors present a software tool kit called MesKit that analyses multi region sequencing studies of cancer. The tool is quite comprehensive with many different capabilities, good documentation and produces high-quality figures. Overall I think this would be a valuable piece of software to the cancer evolution community. I do have a few concerns with regard to the interpretation of some of these analyses which I believe should be appropriately caveated.

Major comments

MesKit enables clustering by VAF to interpret ITH. Without consideration of the copy number of the mutation, VAF clustering will not be informative. The authors should clarify what they are doing here. It is more appropriate to do one of the following: i) cluster using CCFs ii) cluster using some kind VAF corrected by copy number or iii) cluster VAF per copy number state (cluster on mutation only found in diploid regions for example). Another relevant point here is that there is a debate within the field around the use of clustering to identify subpopulation, see for example Caravagna et al Nature Genetics 2020. The main issue is that "clusters" at low CCF/VAF may not represent distinct populations but rather are a mixture of variants present within different lineages. Another issue is that depth of sequencing can have a large influence on the quality of clustering results (again see Caravagna et al Nature Genetics 2020). I think the challenges and possible issues with this type of analysis should be mentioned in the paper and software vignette so that users understand the caveats here.

I think it is highly unlikely that the BRCA2 mutations the authors identify contribute to metastasis in CRC. To probe this further the authors should assess the functional impact of the mutation through cbioportal or the like and assess the copy number zygosity around that locus, appearance of LOH may give some further evidence that these mutations are functional. In general there is little evidence that BRCA2 has a role in CRC development, see Jonsson et al. Nature 2019.

I would like to see some comparison of the signature deconvolution with other methods to ensure that the authors implementation is approximately equivalent to other widely used tools.

Minor comments

There are a number of grammatical errors that need addressing.

The ability to generate phylogenetic trees with different methods is very valuable in my opinion. In the methods the authors comment on how MesKit provides the ability to compare tree similarity using a function called treedist, this is not used in the results as far as I can see. I think including some comparison in the results would be a valuable illustration of how similar (or different) differing approaches may be.

It says in the text that the software is released under the GPL v3 License, but in the github repo, an MIT

license is shown. This should be clarified.
I was able to install the software via devtools but not bioconductor, it appears others have had similar issues - https://github.com/Niinleslie/MesKit/issues/211. Would be good to understand why this might be happening.
In the introduction, I think several commonly used ITH related tools are missing citations such as pyclone, DPclust and phylowgs.
This is not mentioned in the paper, but in the R package the authors provide the ability to perform a "neutrality test" as described in my paper from 2016: Williams et al Nature Genetics. This analysis has been superseded by a new software tool called MOBSTER (https://github.com/caravagnalab/mobster), I think this should be made clear in the vignette so that users know what the current recommended analysis tools are for this type of analysis. I would not expect a new implementation of MOBSTER, just a note in the vignette and function documentation would suffice.
Hu et al. Nature Genetic should be cited when describing Fst, this was the first (only?) study to use this measure to my knowledge and should be cited accordingly.
Marc Williams

Level of Interest

Please indicate how interesting you found the manuscript: Choose an item.

Quality of Written English

Please indicate the quality of language in the manuscript: Choose an item.

Declaration of Competing Interests

Please complete a declaration of competing interests, considering the following questions:

- Have you in the past five years received reimbursements, fees, funding, or salary from an organisation that may in any way gain or lose financially from the publication of this manuscript, either now or in the future?
- Do you hold any stocks or shares in an organisation that may in any way gain or lose financially from the publication of this manuscript, either now or in the future?
- Do you hold or are you currently applying for any patents relating to the content of the manuscript?
- Have you received reimbursements, fees, funding, or salary from an organization that holds or has applied for patents relating to the content of the manuscript?
- Do you have any other financial competing interests?
- Do you have any non-financial competing interests in relation to this paper?

If you can answer no to all of the above, write 'I declare that I have no competing interests' below. If your reply is yes to any, please give details below.

No to all.

I agree to the open peer review policy of the journal. I understand that my name will be included on my report to the authors and, if the manuscript is accepted for publication, my named report including any attachments I upload will be posted on the website along with the authors' responses. I agree for my report to be made available under an Open Access Creative Commons CC-BY license (http://creativecommons.org/licenses/by/4.0/). I understand that any comments which I do not wish to be included in my named report can be included as confidential comments to the editors, which will not be published.

Choose an item.

To further support our reviewers, we have joined with Publons, where you can gain additional credit to further highlight your hard work (see: https://publons.com/journal/530/gigascience). On publication of this paper, your review will be automatically added to Publons, you can then choose whether or not to claim your Publons credit. I understand this statement.

Yes Choose an item.
